# Unraveling the plant diversity of the Amazonian *canga* through DNA barcoding

**DOI:** 10.1002/ece3.8057

**Published:** 2021-08-31

**Authors:** Santelmo Vasconcelos, Gisele L. Nunes, Mariana C. Dias, Jamily Lorena, Renato R. M. Oliveira, Talvâne G. L. Lima, Eder S. Pires, Rafael B. S. Valadares, Ronnie Alves, Maurício T. C. Watanabe, Daniela C. Zappi, Alice L. Hiura, Mayara Pastore, Liziane V. Vasconcelos, Nara F. O. Mota, Pedro L. Viana, André S. B. Gil, André O. Simões, Vera L. Imperatriz‐Fonseca, Raymond M. Harley, Ana M. Giulietti, Guilherme Oliveira

**Affiliations:** ^1^ Instituto Tecnológico Vale Belém Brazil; ^2^ Programa Interunidades de Pós‐Graduação em Bioinformática Universidade Federal de Minas Gerais Belo Horizonte Brazil; ^3^ Instituto de Ciências Biológicas Universidade de Brasília Brasília Brazil; ^4^ Coordenação de Botânica Museu Paraense Emílio Goeldi Belém Brazil; ^5^ Programa de Pós‐Graduação em Ecologia Universidade Federal do Pará Belém Brazil; ^6^ Departamento de Biologia Vegetal Universidade Estadual de Campinas Campinas Brazil; ^7^ Instituto de Biociências Universidade de São Paulo São Paulo Brazil; ^8^ Royal Botanic Gardens, Kew Richmond UK; ^9^ Programa de Pós‐Graduação em Botânica Universidade Estadual de Feira de Santana Feira de Santana Brazil

**Keywords:** Amazon basin, Carajás, DNA barcodes, ITS2, *rbc*L, vascular plants

## Abstract

The *canga* of the Serra dos Carajás, in Eastern Amazon, is home to a unique open plant community, harboring several endemic and rare species. Although a complete flora survey has been recently published, scarce to no genetic information is available for most plant species of the ironstone outcrops of the Serra dos Carajás. In this scenario, DNA barcoding appears as a fast and effective approach to assess the genetic diversity of the Serra dos Carajás flora, considering the growing need for robust biodiversity conservation planning in such an area with industrial mining activities. Thus, after testing eight different DNA barcode markers (*mat*K, *rbc*L, *rpo*B, *rpo*C1, *atp*F‐*atp*H, *psb*K‐*psb*I, *trn*H‐*psb*A, and ITS2), we chose *rbc*L and ITS2 as the most suitable markers for a broad application in the regional flora. Here we describe DNA barcodes for 1,130 specimens of 538 species, 323 genera, and 115 families of vascular plants from a highly diverse flora in the Amazon basin, with a total of 344 species being barcoded for the first time. In addition, we assessed the potential of using DNA metabarcoding of bulk samples for surveying plant diversity in the *canga*. Upon achieving the first comprehensive DNA barcoding effort directed to a complete flora in the Brazilian Amazon, we discuss the relevance of our results to guide future conservation measures in the Serra dos Carajás.

## Graphical abstract

Here we bring genetic information for the unique flora of the *canga* of the Serra dos Carajás in Eastern Amazon. DNA barcodes were obtained for 538 species, 323 genera, and 115 families of vascular plants, with 344 species being characterized for the first time. Upon achieving the first comprehensive DNA barcoding effort directed to a complete flora in the Brazilian Amazon, we discuss the relevance of our results to guide future conservation measures in the region.

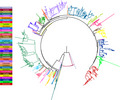

## 1 INTRODUCTION

Conservation efforts depend on a detailed knowledge of the biodiversity in the area of interest, although this is rarely available for megadiverse regions (Alroy, 2017; Hopkins, 2007; Milliken et al., 2010; Myers et al., 2000). The Amazon basin is a vast and diverse biome, being exceptionally important for the maintenance of the biodiversity in the Neotropical region over time (Antonelli et al., 2018). Although the region is undoubtedly one of the most important ecosystems in the planet, harboring an estimated one quarter of all extant plant species, there is a lack of knowledge about a huge portion of the Amazon ecosystems (BFG, 2018; Fearnside, 2002; Hopkins, 2007; Milliken et al., 2010; Morim & Lughadha, 2015). In addition, along its massive geographic area, the Amazon basin is composed of several different centers of endemism (see Silva et al., 2005 and references within), which are important for the resilience of the forests in face of the disturbing effects of direct anthropological impacts and climate change (Levine et al., 2016).

**FIGURE 1:**
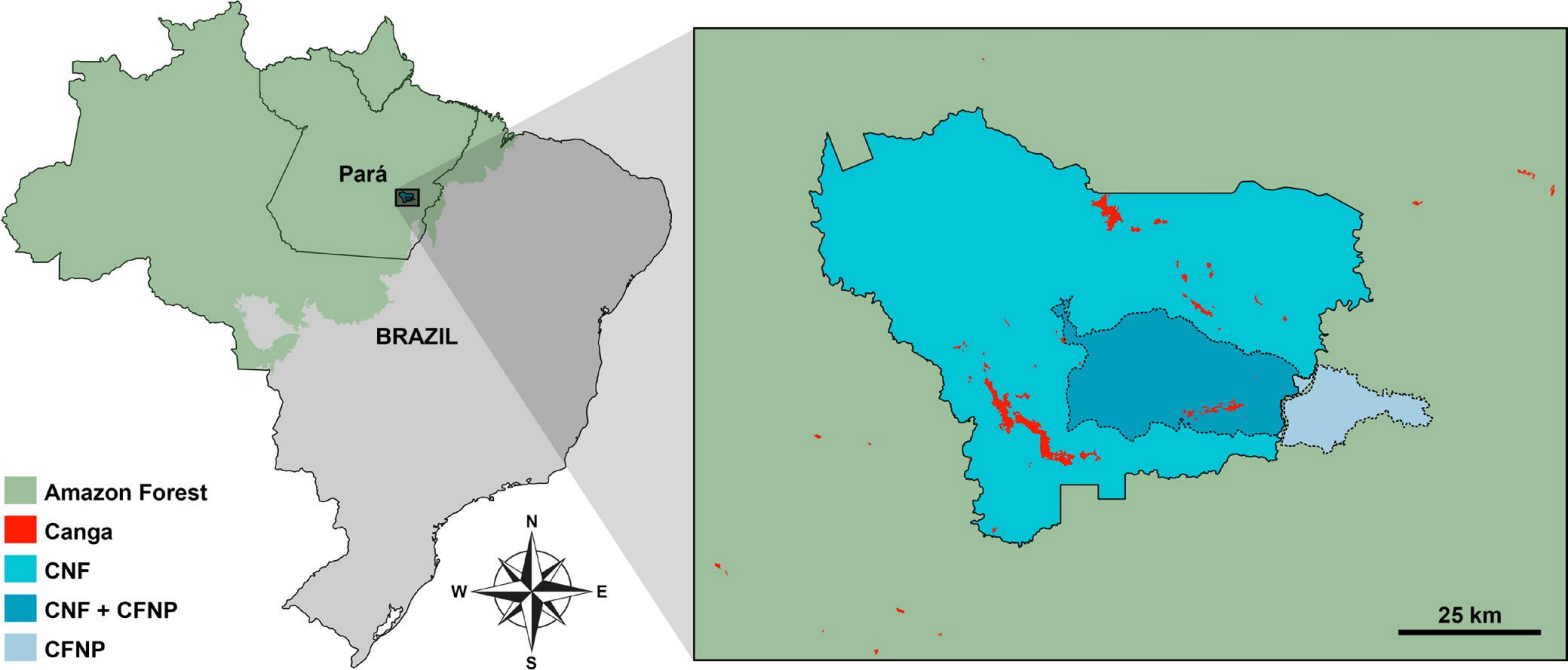
Distribution of the *canga* formation in the Serra dos Carajás, Pará, Brazil. The circumscriptions of the Carajás National Forest (CNF) and Campos Ferruginosos National Park (CFNP) are evidenced within the Amazon Forest

The Serra dos Carajás (Figure 1), Eastern Amazon, in the southeast of the Brazilian state of Pará, is formed by ironstone outcrops covered by a formation known as *campos rupestres* on *canga* (as detailed in Souza‐Filho et al., 2019; Zappi et al., 2019), surrounded by a dense forest matrix. The *canga* of the Serra dos Carajás is found mostly in the Carajás National Forest (Floresta Nacional de Carajás, or FLONA de Carajás), harboring several endemic and rare plant species, such as *Philodendron carajasense* E. G. Gonç. (Araceae) and *Carajasia cangae* R. M. Salas, E. L. Cabral & Dessein (Rubiaceae) (Giulietti et al., 2019; Skirycz et al., 2014; Viana et al., 2016), with a high floristic heterogeneity among sites (Zappi et al., 2019). Such ironstone outcrops have been explored throughout the years mainly for iron ore mining activities (Skirycz et al., 2014), and robust biodiversity surveys are necessary to ensure species protection through effective conservation efforts in the presence of industrial activities, especially in view of the climate change scenarios predicted for the region (Giannini et al., 2020; Levine et al., 2016; Miranda et al., 2019).

Plant surveys in the Serra dos Carajás started in the 1970s, as detailed by Viana et al. (2016). However, a project to publish its flora, the Flora of the *canga* of Carajás (FCC), took part in just under 4 years, being the first complete Flora for a region of the Brazilian Amazon (Mota et al., 2018). This project provided complete floristic treatments for 116 angiosperm families, comprising approximately 900 species (Mota et al., 2018), a number considerably higher than the initial estimate of around 600 species (Viana et al., 2016). Other vascular plant groups detailed in the FCC included 175 ferns in 22 families, 11 lycophytes in three families (Salino et al., 2018), and a single gymnosperm, *Gnetum nodiflorum* Brongn. (Gnetaceae), a liana widely distributed in the Brazilian Amazon (Mota & Giulietti, 2016).

The systematic collection of DNA samples was taken on board as part of the floristic initiative of the FCC project (Mota et al., 2018), as the availability of genetic and genomic data of plants were seen from the onset as extremely important. Such a measure would ensure the correct identification of the species, which had been authenticated by taxonomist specialists, and backed by a deposited voucher, thus guiding more effectively all conservation efforts for the area.

The application of DNA barcodes (Hebert et al., 2003) stands out as an efficient source of reliable and cost‐effective information for identifying and measuring the diversity status of natural populations of plant species of the *canga*, as recently demonstrated for endemic species such as the morning‐glory *Ipomoea cavalcantei* D. F. Austin (Convolvulaceae), and the quillworts *Isoetes cangae* J. B. S. Pereira, Salino & Stützel and *Isoetes serracarajensis* J. B. S. Pereira, Salino & Stützel (Isoetaceae), by Babiychuk et al. (2017) and Nunes et al. (2018), respectively. However, it is a well‐known fact that the development of DNA barcodes is not as straightforward for plants as for other eukaryotes, such as animals and fungi (Fazekas et al., 2009; Hebert et al., 2016; Hollingsworth et al., 2016). The main problems associated with DNA barcoding of plant species arise with the considerably slower pace of evolution of the organelle genomes and the universality of some chloroplast DNA (cpDNA) markers, mainly those with higher nucleotide substitution rates within the plastomes, such as the *mat*K gene (Hollingsworth et al., 2011). Also, there is a difficulty in standardizing which cpDNA regions will function as reliable plant DNA barcodes, since several authors have been reporting variable success rates using different markers (e.g., *rpo*B, *rpo*C1, *atp*F‐*atp*H, *psb*K‐*psb*I, and *trn*H‐*psb*A) (e.g., Fazekas et al., 2008), although the combination of the *rbc*L and *mat*K sequences has been recommended as the core barcoding loci (CBOL Plant Working Group, 2009; Kress, 2017). Besides organelle markers, some regions of the nuclear genome, such as the internal transcribed spacers (ITS1 and ITS2) of the 35S rRNA gene, yield useful DNA barcodes for plants (Chen et al., 2010; Hollingsworth et al., 2011).

Furthermore, the generation of DNA barcodes at the species level enables the use of composite samples for detection of species from a given environment, known as DNA metabarcoding. This approach has been regarded as a robust, fast, and cost‐effective approach for automated multispecies identification (Deiner et al., 2017; Zinger et al., 2019). For plants, ITS2 has been one of the main markers of choice for surveying multiple species at once, considering the methodological advantages of using this DNA barcode, such as the ease of standardizing PCR conditions and a smaller amplicon size (~450 bp) in comparison with other frequently used regions (Chen et al., 2010; Gous et al., 2019; Richardson et al., 2015). Thus, a curated DNA barcode library and well‐established analytical procedures can provide the basis for the successful application of DNA metabarcoding for monitoring biodiversity (Adamowicz et al., 2019; Dormontt et al., 2018; Kress, 2017).

To the best of our knowledge, there is no other DNA barcoding approach directed to the complete flora of any other region in the Amazon basin. Hence, we describe DNA barcodes for vascular plant species mainly focusing on the *canga* of the Serra dos Carajás, also including plants other from areas in the Brazilian state of Pará that are relevant to an understanding of the biodiversity composition of this mountain range as a whole. We tested the potential of eight commonly used DNA barcode regions and then chose the most suitable markers for a broader application of the DNA barcoding approach in the area, in order to provide robust tools to assess genetic diversity data of the flora of the Amazon basin. Here we followed two main premises: (a) the highest possible marker universality, considering the diversity of taxonomic groups in the *canga*; and (b) a reasonable standardization and automation of the protocols for sample processing and analyses. Moreover, we aimed to test the potential of DNA metabarcoding analyses with ITS2 for future applications in the Serra dos Carajás, taking advantage of the DNA barcode library developed here.

## 2 MATERIALS AND METHODS

### 2.1 Plant materials for the DNA barcode procedures

Preferentially, young leaf tissues were sampled for the DNA extractions, although either other vegetative or reproductive structures were employed when needed, as in the case of species of Cactaceae and Eriocaulaceae, for instance. A total of 1,179 specimens of vascular plants from 120 families, 343 genera, and 577 species were collected in the Serra dos Carajás and other relevant regions in Eastern Amazon, state of Pará, Brazil (Table A1), as part of the FCC project (Mota et al., 2018; Salino et al., 2018; Viana et al., 2016), under ICMBio/MMA permit numbers 47856‐2, 48272‐6, 53990‐1 and 63324‐1. Approximately 55% of those samples (645 specimens from 96 families, 243 genera, and 370 species) were used to test seven different cpDNA regions (the genes *mat*K, *rbc*L, *rpo*B, and *rpo*C1, and the intergenic spacers *atp*F‐*atp*H, *psb*K‐*psb*I, and *trn*H‐*psb*A) and the ITS2 intergenic region. The remaining 534 samples were barcoded only after the selection of the two best markers (*rbc*L and ITS2), as detailed below. The vouchers of all sampled specimens were deposited at the MG herbarium (Museu Paraense Emílio Goeldi, Belém, Pará, Brazil) (Table A1).

Most samples (983, ca. 87%) were collected in 2% CTAB‐NaCl saturated buffer, as described by Rogstad (1992), and then stored under refrigeration (~4°C) until the DNA extraction was carried out. The remaining collected tissues (147, ca. 13%) were dried in silica gel and then stored at room temperature (~25°C) until processing.

### 2.2 DNA extraction

For the DNA extractions, we established an efficient automated protocol for all plant materials, considering the high diversity of taxonomic groups observed in the *canga* of the Serra dos Carajás. Approximately 20 mg of fresh plant tissue (or ~10 mg for silica dried samples) was separated in 96 racked 1.2‐ml collection microtubes (Axygen) with two 3 mm tungsten carbide beads (Qiagen). The samples were frozen in a deep freezer (−80°C) for 18–20 hr and then ground in a TissueLyser II (Qiagen) for 1 min at 30 Hz. Then, 600 µl of extraction buffer (2% w/v CTAB, 0.1 mM Tris‐HCl, 20 mM EDTA, 1.4 M NaCl) was added to the ground material and the samples were incubated for 40 min at 60°C in a water bath. The collection microtubes were centrifuged for 1 min at 2,900× *g* to eliminate debris, and 300 µl of the supernatant was transferred to a 96 deep‐well U‐bottom plate. Afterward, an automated extraction was performed in a QIAcube HT (Qiagen) with the “Q protocol V1” of the QIAamp 96 DNA Kit (Qiagen), with minor modifications regarding the sample preparation step, which was carried out without the VXL buffer and including an incubation for 30 s after adding 350 µl of binding buffer ACB, mixing for six times. Also, for some difficult samples, the DNA extractions were performed using the CTAB protocol I described by Weising et al. (2005), with minor modifications (0.5–1.0 g of leaf tissue and 10 ml of the extraction buffer, with the addition of 4% w/v PVP and 0.2% v/v β‐mercaptoethanol), followed by the selective precipitation of polysaccharides described by Michaels et al. (1994).

### 2.3 DNA barcode generation and phylogenetic analyses

The PCR conditions and sequencing reactions were performed as described in Babiychuk et al. (2017), using the primers listed in the Table A2. We used PIPEBAR (Oliveira et al., 2018) to process all trace files (*.ab1 and *.phd) to generate the assembled consensus of the forward and reverse sequences. Afterward, to check initially for problematic sequences (from either mislabeled or contaminated samples) generating unusual specimen groupings, considering mainly order and family affiliations, the sequences were aligned with MAFFT 7.388 using the algorithm *Auto* (Katoh & Standley, 2013) for each marker separately. Then, phylogenetic trees based on maximum likelihood (ML) were constructed with RAxML 8.2 (Stamatakis, 2014) as implemented in the CIPRES portal (http://phylo.org), using the substitution model GTR + G and rapid bootstrapping with 1,000 replicates. Furthermore, we performed BLASTn searches in the GenBank database (http://blast.ncbi.nlm.nih.gov/Blast.cgi) for additional quality control to avoid problematic sequences, especially in the case of the intergenic regions, which were considerably more difficult to align due to the high taxonomic diversity among the sampled specimens.

Finally, we tested the phylogenetic resolution by counting monophyletic species with at least 70% of bootstrap support, considering only those with more than one sampled specimen. ML trees were constructed in RAxML as described above, using six different matrices based on the *rbc*L and ITS2 alignments, including a topology constraint for the family relationships based on Gastauer and Meira Neto (2017), with minor modifications considering Mota et al. (2018), and PPG I (2016): two concatenated matrices—(a) *rbc*L + ITS2, considering the complete sampling, including accessions with missing sequences of one of the two markers, and (b) *rbc*L + ITS2_reduced, considering only specimens with both barcodes; and four matrices from separate alignments—(c) *rbc*L and (d) ITS2, based on the complete sampling, (e) *rbc*L_reduced and (f) ITS2_reduced, based on the reduced sampling used in the second matrix. All alignments and phylogenetic trees are available in the Open Science Framework (OSF) repository (Supplementary Data and Supplementary Figures S01–S12; https://doi.org/10.17605/osf.io/5xt3u).

### 2.4 Barcode analysis

To test the barcode resolution (as the percentage of correctly assigned species) of the eight different markers as barcodes, all‐to‐all BLAST searches were performed with the sequences obtained herein (both as query and local database), as described in Burgess et al. (2011), using the BLASTn plugin in Geneious Prime 2019.2.3 (Biomatters). Thus, we considered a correct assignment whether a given query sequence presented 100% pairwise identity only with the species itself, in the cases of just one available sequence for the species (336 spp.; 61.9%), such as *Mandevilla tenuifolia* (J. C. Mikan) Woodson (Apocynaceae), or when the intraspecific pairwise identities were either similar or higher when compared with accessions of other species, in the cases of species with more than one specimen with a barcode (207 spp.; 38.1%), such as *Mandevilla scabra* (Hoffmanns. ex Roem. & Schult.) K. Schum. Additionally, we tested whether the combination of the *rbc*L and ITS2 barcodes (*rbc*L + ITS2) would significantly increase species resolution, following Burgess et al. (2011). Besides, searches were performed in the BOLD database (http://www.boldsystems.org) to check whether there was any barcode previously published for the species analyzed in this work, and all sequences produced were deposited in the referred database under the accession numbers listed in the Table A1.

### 2.5 Metabarcoding analysis

To assess the potential of using metabarcoding analysis with bulk samples for surveying plant diversity in the *canga* in future monitoring approaches, we sampled all discernible plant specimens within an approximate 10 m radius in six plots, including two markedly different vegetation types (forest groves and open rupestrian vegetation; Table A3), near the end of the dry season (27 and 28 September and 2017) that lasts from May to October (see Viana et al., 2016). Although virtually all plants were sterile, field activities are considerably safer in the ironstone fields during the dry season (e.g., Sodré et al., 2020). For each sampled locality, pieces of young leaves with approximately 1 cm^2^ were collected in a 50‐ml Falcon tube containing 30 ml of the 2% CTAB‐NaCl saturated buffer and then stored as previously described.

The procedures for DNA extraction using CTAB and selective precipitation of polysaccharides followed as mentioned above, except for the amounts of leaf tissue (8 g) and extraction buffer (15 ml) per sample. Likewise, the amplification of the ITS2 region followed the same PCR conditions as before, with minor modifications, including 1× TBT‐PAR buffer (Samarakoon et al., 2013) and using the primers ITS2‐S2F (Chen et al., 2010), with the adapters Ion A, and ITS4 (White et al., 1990), with the adapter trP1. Then, PCR products were purified with the kit Agencourt AMPure XP Beads (Beckman Coulter), following manufacturer's instructions. Each of the six different libraries (one library per collection plot) was composed by pooling four independent PCR replicates and sequenced using the Ion PGM platform (Thermo Fisher).

Raw data from the single‐end sequencing run were processed using FASTX Toolkit (http://hannonlab.cshl.edu/fastx_toolkit) and the R package DADA2 (Callahan et al., 2016) to correct sequencing errors and infer exact amplicon sequence variants (ASVs) (equivalent to OTU determination). An ASVs table was created, and representative sequences were assigned to taxa with BLASTn using our ITS2 library as a local reference database, based on minimum similarity and coverage settings (‐perc_identity 95 and ‐qcov_hsp 70). Finally, we used the LULU curation algorithm with default settings to collapse erroneous ASVs, minimum relative co‐occurrence of 0.95, and the default minimum similarity threshold of 84% (Frøslev et al., 2017). Additionally, downstream analyses were performed with the R package Phyloseq v1.26.1 (McMurdie & Holmes, 2013), with an object built from the ASVs curated version, using data from taxonomy assignments and sampling plots.

## 3 RESULTS

### 3.1 Amplification and sequencing success of barcodes

Considering only the initial test with the eight markers assessment using 645 samples and 370 species, the proportions of barcoding success (94.73% and 93.24%, respectively) were similar to the complete sampling including the specimens barcoded only with *rbc*L and ITS2. Our results clearly showed *rbc*L (503 samples; 304 species) and ITS2 (490; 286) to be the best barcode regions, with the highest species coverage (Table 1; Table A1). On the other hand, *mat*K (154; 126), *rpo*B (136; 119), *rpo*C1 (156; 126), *atp*F‐*atp*H (176; 127), *psb*K‐*psb*I (125; 95), and *trn*H‐*psb*A (109; 77) presented considerably lower numbers of generated sequences, especially these last two regions (Table 1; Table A1). Out of the eight species with neither *rbc*L nor ITS2 barcodes, five had sequences of just one of the remaining markers (*Carajasia cangae*, Rubiaceae—*rpo*B; *Sinningia minima* A. O. Araujo & Chautems, Gesneriaceae—*rpo*B; *Myrcia tenuiflora* A. R. Lourenço & E. Lucas, Myrtaceae—*rpo*C1; *Stachytarpheta glabra* Cham., Verbenaceae—*atp*F‐*atp*H; and *Hemionitis palmata* L., Pteridaceae—*trn*H‐*psb*A), while three had more than one barcode (*Justicia potamogeton* Lindau, Acanthaceae—*mat*K, *rpo*C1, and *atp*F‐*atp*H; *Picramnia ferrea* Pirani & W. W. Thomas, Picramniaceae—*rpo*B and *atp*F‐*atp*H; and *Senna latifolia* (G. Mey.) H. S. Irwin & Barneby, Fabaceae—*mat*K, *rpo*B, and *rpo*C1) (Table A1). Additionally, we obtained sequences of all eight barcode markers for only six species: *Aegiphila integrifolia* (Jacq.) Moldenke (Lamiaceae), *Ctenanthe ericae* C. L. Andersson (Marantaceae), *Eriocaulon cinereum* R. Br. (Eriocaulaceae), *Helanthium tenellum* (Mart.) Britton (Alismataceae), *Jacquemontia tamnifolia* (L.) Griseb. (Convolvulaceae), and *Pilocarpus carajaensis* Skorupa (Rutaceae) (Table A1).

**TABLE 1:** Barcode resolution based on BLAST searches, using the generated DNA barcode library as both query and local database

Marker	IS^a^	NIS^b^	NA^c^	%IS^d^	%SS^e^
*rbc*L	253	51	39	83.22	88.63
*rbc*L_reduced^f^	215	40	‐	84.31	74.34
ITS2	265	21	57	92.66	83.38
ITS2_reduced^f^	234	21	‐	91.76	74.34
*rbc*L + ITS2	310	25	8	92.54	97.67
*rbc*L + ITS2_reduced^f^	241	14	‐	94.51	74.34
*mat*K	118	8	217	93.65	36.73
*rpo*B	113	6	224	94.96	34.69
*rpo*C1	114	12	217	90.48	36.73
*atp*F‐*atp*H	119	8	216	93.70	37.03
*psb*K‐*psb*I	88	7	248	92.63	27.70
*trn*H‐*psb*A	75	2	266	97.40	22.45

^a^ IS, number of correctly identified species.

^b^ NIS, number of nonidentified species.

^c^ NA number of species with no available sequences.

^d^ %IS, percentage of correctly identified species.

^e^ %SS, percentage of species with available sequences.

^f^ Including only the species with sequences of both *rbc*L and ITS2.

Afterward, considering the additional 534 samples, we obtained sequences of *rbc*L and ITS2 for another 183 and 140 species (393 and 425 samples), respectively. Almost all barcoded species had, at least, sequences of either *rbc*L or ITS2 (527 out of 535 spp.; 98.50%), from which 399 (75.71%) presented both barcodes (Table A1).

From our complete sampling, considering the 645 specimens used in the initial test with eight markers, plus the 534 remaining samples barcoded using only *rbc*L and ITS2, we obtained valid sequences of at least one of the eight markers for 538 out of the 575 sampled species (93.56%), totaling 1,130 specimens (95.84%) and 2,729 DNA barcodes (Table A1). After searching for previous records in the BOLD database, we observed that 344 (63.94%) of those species were barcoded for the first time in the present work (Table A1). In addition, 33 out of the 323 genera with species barcoded here (10.22%) did not have any sequence available in the BOLD database, with several of them being from speciose and representative families in the *canga*, such as Asteraceae (*Cavalcantia*, *Monogereion*, *Parapiqueria,* and *Praxelis*) and Poaceae (*Actinocladum*, *Hildaea*, *Paratheria*, *Parodiolyra*, *Raddiella*, *Rhytachne,* and *Trichanthecium*) (Table A4). Considering the sampled families, we obtained sequences for 115 out of 120 (95.83%) (Table A1).

On the other hand, a total of 49 samples (4.16%) from 37 species (6.43%) could not be barcoded due to problems either with the PCRs or in generating sequencing reads with minimally required quality levels. Balanophoraceae, Begoniaceae, Dilleniaceae, Hydrocharitaceae, and Trigoniaceae were the only families without representatives with a valid barcode sequence. Samples from some taxa were markedly more challenging to process with the “universal” protocols adopted in this work, and Melastomataceae species were strikingly problematic. Considering that out of 19 specimens from 13 species and eight genera, only a total of five samples from four species (*Bellucia grossularioides* (L.) Triana, *Miconia heliotropoides* Triana, *Noterophila crassipes* (Naudin.) Kriebel & M. J. R. Rocha, and *Tibouchina* sp.) were successfully barcoded (Table A1).

It is noteworthy, however, that samples of several species that yielded good quality DNA and amplicons in their expected size ranges presented poor sequencing results for either one or both reads. This problem was more evident in the cases of the cpDNA intergenic regions, for which sequencing results commonly generated electropherograms with many superposed peaks due to the presence of mononucleotide repeats, as observed in reads of *atp*F‐*atp*H sequences of *Ipomoea cavalcantei*, for instance (Supplementary Figure S13; https://osf.io/5xt3u/).

### 3.2 Barcode resolution

Considering the initial test with the eight markers, we observed levels of barcode resolution (percentage of identified species) above 90% for almost all regions, with 97.40% for *trn*H‐*psb*A, 94.96% for *rpo*B, 93.65% for *mat*K, 93.70% for *atp*F‐*atp*H, 92.66% for ITS2, 92.63% for *psb*K‐*psb*I, and 90.48% for *rpo*C1 (Table 1). The *rbc*L marker presented the lowest barcode resolution, with 83.22% of the species successfully identified (Table 1). On the other hand, the combined resolution of the two tested markers with the best sequencing results (*rbc*L + ITS2, 92.54%) was much higher than with *rbc*L but slightly lower than the percentage obtained for ITS2. (Table 1). Excluding the species without one of these two markers, the combined markers presented a higher proportion of identified species than the resolution of both regions alone (*rbc*L + ITS2_reduced = 94.51%; ITS2_reduced = 91.76%; *rbc*L_reduced = 84.31%; Table 1).

Considering the complete sampling with *rbc*L and ITS2, that includes the 645 specimens from the test and the 534 additional samples barcoded only with these two regions, the barcode resolution levels were lower, since *rbc*L, ITS2, and *rbc*L + ITS2 presented 75.00%, 89.45%, and 86.06%, respectively (Table 2). Similar to the marker test, when excluding the species with only one of the markers, the marker combination presented a higher proportion of identified species than the regions alone (*rbc*L + ITS2_reduced = 90.59%; ITS2_reduced = 89.31%; *rbc*L_reduced = 81.17%; Table 2). Moreover, we observed a different barcode resolution pattern when comparing species with only one or with more than one accession. In the case of the species with a single barcoded specimen, the resolution values of both markers (*rbc*L with 77.60% for 317 spp.; and ITS2 with 93.64% for 283 spp.) were considerably higher than for the species with at least two accessions available (*rbc*L with 68.85% for 183 spp.; and ITS2 with 78.21% for 153 spp.) (Table 2). Likewise, for most genera represented by a single barcoded species, we also observed considerably higher levels of resolution (91.83% and 95.65% for *rbc*L and ITS2, respectively) in comparison with species from the genera with more than one sampled species (67.05% and 86.67% for *rbc*L and ITS2, respectively).

**TABLE 2:** Barcode resolution based on BLAST searches, using the generated DNA barcode library as both query and local database, and phylogenetic resolution of *rbc*L and ITS2, considering only nodes with bootstrap support (BS) ≥ 70%

Marker	IS^a^	NIS^b^	NA^c^	%IS^d^	%SS^e^	SSA^f^	SMA^g^	MS^h^	NMS^i^	%SMA^j^	%MS^k^
*rbc*L	369	123	46	75.00	91.45	309	183	114	69	37.20	62.30
*rbc*L_reduced^l^	319	74	145	81.17	73.05	259	134	91	43	34.10	67.91
ITS2	390	46	102	89.45	81.04	283	153	115	38	35.09	75.16
ITS2_reduced^l^	351	42	145	89.31	73.05	259	134	102	32	34.10	76.12
*rbc*L + ITS2	463	67	8	86.06	98.51	324	206	136	70	38.87	66.02
*rbc*L + ITS2_reduced^l^	356	37	145	90.59	73.05	259	134	107	27	34.10	79.85

Note

The analyses included specimens of all 543 species of the *canga* of Serra dos Carajás and other regions in the Brazilian state of Pará, Eastern Amazon (Table A1). The detailed data on the barcode resolution (IS^a^) and phylogenetic resolution (MS^h^) of each species are available in the Supplementary Table S3 (https://osf.io/5xt3u/).

^a^ IS, number of correctly identified species.

^b^ NIS, number of nonidentified species.

^c^ NA number of species with no available sequences.

^d^ %IS, percentage of correctly identified species.

^e^ %SS, percentage of species with available sequences.

^f^ SSA, number of species with a single accession with available sequences.

^g^ SMA, number of species with more than one accession with available sequences.

^h^ MS, number of species recovered as monophyletic, with BS ≥ 70%.

^i^ NMS, number of species recovered either as nonmonophyletic or with BS > 70%.

^j^ %SMA, percentage of species with more than one accession.

^k^ %MS, percentage of species recovered as monophyletic, with BS ≥ 70%.

^l^ Including only the species with sequences of both *rbc*L and ITS2.

### 3.3 Phylogenetic resolution

**FIGURE 2:**
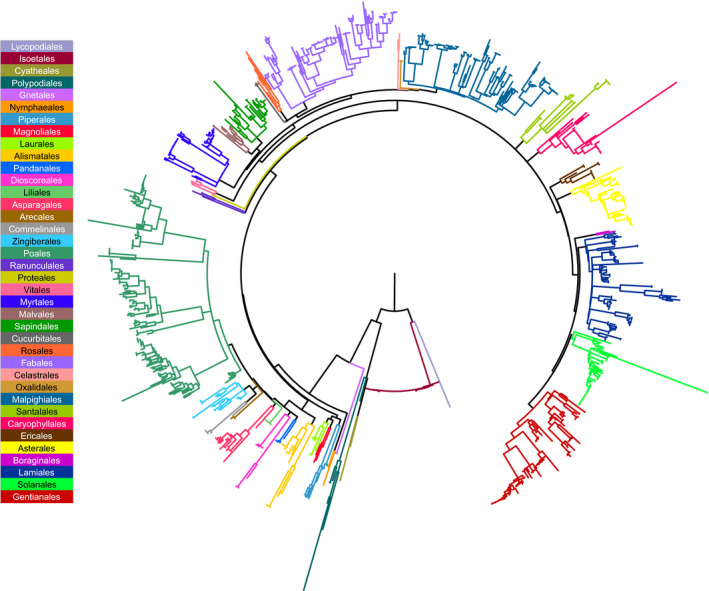
Maximum likelihood tree from the *rbc*L and ITS2 concatenated matrix of the *canga* plants of the Serra dos Carajás and related regions in the Eastern Amazon. The colored branches correspond to the listed orders. The trees bringing the detailed information on the accessions and support values using the six different matrices are available in the OSF repository (Supplementary Figures S01–S13; https://osf.io/5xt3u/)

Among the phylogenetic trees obtained from the six used matrixes (Figure 2; Supplementary Figures S01–S06, https://osf.io/5xt3u/), the proportions of species with more than one accession were close, ranging between 34.10% and 38.87% (Table 2). On the other hand, we observed a wider variation in the percentage of monophyletic species recovered by each matrix, ranging from 62.30% for *rbc*L and 79.87% for *rbc*L + ITS2_reduced (Table 2). Considering both complete and reduced matrixes, the ITS2 marker presented higher phylogenetic resolution (75.16% for ITS2 and 76.12% for ITS2_reduced) than *rbc*L (62.30% for *rbc*L and 67.91% for *rbc*L_reduced) (Table 2). Interestingly, the concatenated matrices presented contrasting patterns of phylogenetic resolution (Table 2). The *rbc*L + ITS2 matrix presented a considerably lower proportion of monophyletic species (66.02%) than the ITS2 matrix (75.16%) (Table 2). Conversely, the phylogenetic resolution of *rbc*L + ITS2_reduced (79.85%) was higher than both equivalent independent matrices (*rbc*L_reduced and ITS2_reduced, with 67.91% and 76.12%, respectively) (Table 2).

Additionally, most of the species correctly identified in the barcode resolution analysis were recovered as monophyletic. Nevertheless, some of the species correctly identified by the DNA barcodes (with barcode resolution) were not resolved in the phylogenies, such as *Clitoria falcata* Lam. (Fabaceae), which was correctly identified in the BLAST analyses with both *rbc*L and ITS2, although appearing as polyphyletic in all six trees. Correspondingly, the opposite situation, in which the species were monophyletic in all trees but without barcode resolution, was also observed, as in the case of *Lindernia brachyphylla* Pennell (Linderniaceae).

### 3.4 Metabarcoding analysis

**FIGURE 3:**
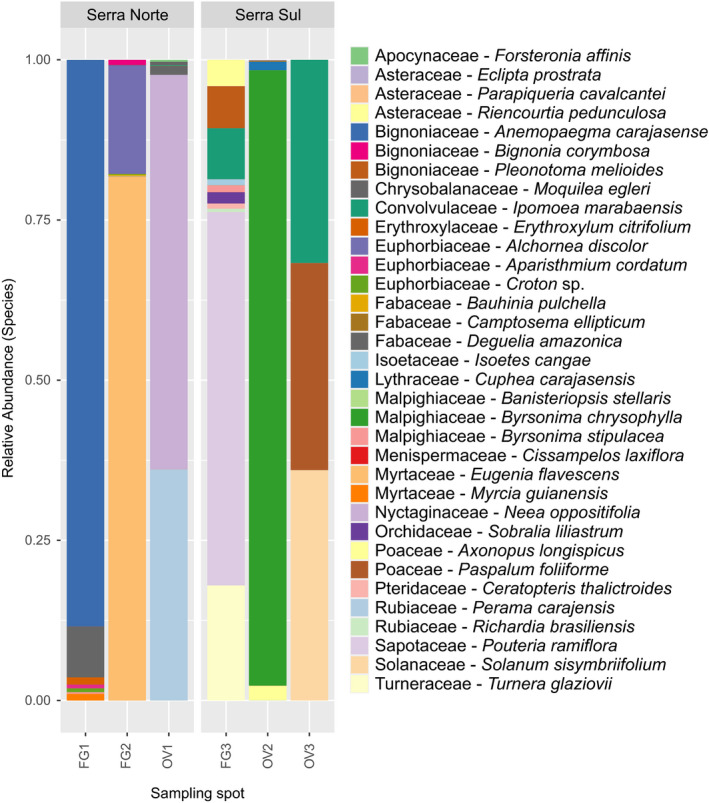
Relative abundance of the observed species in the DNA metabarcoding analysis with bulk samples collected in six different *canga* plots in the Serra dos Carajás, as detailed in the Table A3

The ITS2 high‐throughput amplicon sequencing generated 4,465,309 raw reads from the composite samples of the six plots (Table A3) in the Serra dos Carajás. After the quality control step, 2,269,135 high‐quality reads remained, yielding an average length of 314 bp. A total of 508 different ASVs were observed in the metabarcoding analysis after sequence filtering, then being grouped into 41 ASVs classified to the species level, considering 95% and 70% of sequence similarity and coverage, respectively, resulting in 34 identified species, belonging to 33 genera, 21 families, and 14 orders (Figure 3). Malpighiales was the most representative order, with nine species, followed by Asterales, Fabales, Gentianales, Lamiales, and Myrtales with three species each (Figure 3). In general, the distribution of taxa among areas was quite variable, with most observed species being associated with a single collection plot, such as the endemics *Cuphea carajasensis* Lourteig (Lythraceae) *Parapiqueria cavalcantei* R. M. King & H. Rob. (Asteraceae), and *Perama carajensis* J. H. Kirkbr. (Rubiaceae) (Figure 3). On the other hand, *Byrsonima stipulacea* A. Juss. (Malpighiaceae), *Croton* sp. (Euphorbiaceae), *Eugenia flavescens* DC. (Myrtaceae), *Forsteronia affinis* Müll. Arg. (Apocynaceae), *Ipomoea marabaensis* D. F. Austin & Secco (Convolvulaceae), *Moquilea egleri* (Prance) Sothers & Prance (Chrysobalanaceae), *Richardia brasiliensis* Gomes (Rubiaceae), and *Sobralia liliastrum* Salzm. ex Lindl. (Orchidaceae) could be identified from samples of at least two different areas.

## 4 DISCUSSION

### 4.1 Establishing a reliable DNA barcode library for the flora of the Amazonian *campo rupestre* on *canga*

The practice of identifying species using DNA sequences is quite old and became mainstream after its formalization by Hebert et al. (2003), as pointed out by DeWalt (2011). The implementation of DNA barcoding approaches for plants was slower and more complex than for animal species (see Fazekas et al., 2009). Nevertheless, the importance of DNA barcodes in surveying plant diversity has been extensively acknowledged during the last decade, despite the inherent difficulties of establishing universal and practical methodologies to be applied in a wide range of taxonomic groups from different ecosystems, considering the particularities observed in several taxa (Hollingsworth et al., 2016; Kress, 2017; Lima et al., 2018). Therefore, we initially tested eight of the most used DNA barcode regions (Fazekas et al., 2008; Lima et al., 2018). This evaluation was important to establish *rbc*L and ITS2 as the best markers for barcoding vascular plants of the Serra dos Carajás, covering the principles of standardization, minimalism, and scalability (Hollingsworth et al., 2011), considering the vastly diverse flora of the region, as recently inventoried by the FCC project (Mota et al., 2018; Salino et al., 2018; Viana et al., 2016).

Considering all vascular plants listed for the *canga* by the FCC project (Mota et al., 2018; Salino et al.; 2018; Viana et al., 2016), our DNA barcodes covered approximately one‐third of the species diversity (378 out of 1,044 spp.; 36.21%). However, it is essential to emphasize that, despite the fact that the majority of *canga* species still lack barcodes, the total number of species barcoded herein (with 344 out of 538 being barcoded for the first time) characterizes this work as the most extensive DNA barcoding effort for the Brazilian Amazon up to date. Also, it is important to note that many of the species described for the *canga* are rare and/or difficult to obtain a minimally satisfactory amount of tissue to extract DNA from some being known only from their type collections, such as the elusive orchid *Uleiorchis longipedicellata* A. Cardoso & Ilk.‐Borg. (see Giulietti et al., 2019). On the other hand, sequences for an additional 11 families and 160 species not included in the published lists of the FCC project were obtained, from which 91 were collected in the lowland forest surrounding the *canga* outcrops of the Serra dos Carajás, and 69 from other localities, focusing on the Brazilian state of Pará.

Although defined as one of the two core barcode regions alongside *rbc*L (CBOL, 2009), *mat*K performed poorly in our samples, with amplification and/or sequencing problems in approximately three‐fourths of the tested specimens. We obtained even worse results for *trn*H‐*psb*A, with less than 20% of our samples generating valid sequences, which is surprising since this intergenic region has been one of the preferred alternative barcode markers in several studies (e.g., Erickson et al., 2014; Lahaye et al., 2008). As we have related above, it is paramount to emphasize that many samples were successfully amplified, although the cpDNA intergenic regions presented unsatisfactory sequence data recovery, especially in the case of *trn*H‐*psb*A. Throughout the history of plant DNA barcoding, there have been several reports of methodological problems with most of the regions tested so far, as frequently reported for *mat*K, which depend on several PCR optimizations for different taxa (e.g., CBOL, 2009; Fazekas et al., 2008; Ghorbani et al., 2017; Liu et al., 2015). On the other hand, the almost fully universal nature of many primers designed to amplify and sequence portions of the *rbc*L gene, obviously including the primer pair we used here, makes this marker the safest choice among the known options in terms of building a comprehensive barcode library for a given flora, even taking into account its lower polymorphism levels among closely related species (Hollingsworth et al., 2011).

Nuclear rDNA‐based sequences have been successfully used as DNA barcodes for fungi, especially the ITS region, which is largely employed as the official barcode region for the group (Badotti et al., 2017; Schoch et al., 2012; Wurzbacher et al., 2019). Several authors have emphasized the enormous potential of the ITS components for plant barcoding, which are also frequently regarded as highly informative for resolving phylogenetic relationships (e.g., Liu et al., 2015; Saha et al., 2017; Vasconcelos et al., 2018). Nevertheless, reports of problems with sequence recovery of the complete ITS (including its three regions—ITS1, rDNA 5.8S, and ITS2) are not rare for plants, mainly due to issues related to paralogs and pseudogenes (Álvarez & Wendel, 2003; Feliner & Rosselló, 2007). Gonzalez et al. (2009), for instance, obtained poor sequencing results for ITS, with only 41% of the sampled Amazonian trees being successfully barcoded by the authors. On the other hand, the smaller ITS2 region has been indicated as one of the best regions for plant barcoding, presenting a high rate of sequencing success even for lower quality DNA samples (Chen et al., 2010; Kuzmina et al., 2012; Ramalho et al., 2018). Likewise, our data showed the usefulness of ITS2 as the second‐best tested marker in terms of sequence recovery, with valid barcodes for 81.04% of the species and 81.33% of the samples and performing relatively close to *rbc*L (91.45% of the species and 79.38% of the samples). Obviously, the availability of sequences of a given marker in public repositories is essential for an effective inventory of plant diversity, and ITS2 has been one of the most frequently used barcode regions for angiosperms so far, accounting for 26.7% of the ca. 340,000 sequences available in the BOLD database (up to 20 January 2021), only behind *rbc*L and *mat*K, with 35.8% and 31.6%, respectively.

### 4.2 Species resolution

Assessing the levels of species discrimination in DNA barcoding approaches is undoubtedly important, although comparing results from different analyses is not as straightforward as one may assume. The first (and perhaps the most important) considerations are related to the study area and sampling coverage. DNA barcoding‐specific local floras within a well‐delimited geographic area, such as the *campo rupestre* on *canga* of the Amazon ironstone fields, for instance, may appear to be more limited in scope than studying the plant diversity of whole countries or broader geographic regions. However, with an original area of ca. 144 km^2^ (Souza‐Filho et al., 2019), the *canga* of Carajás harbor roughly as many species of vascular plants as Wales, which in fact is a small country, but with an area 152 times larger than the *campo rupestre* on *canga* of Carajás, and with the whole catalogue of 1,143 species of seed plants already barcoded (de Vere et al., 2012). Also, there are basically two main approaches to assess the capability of correctly identifying species (species resolution) of DNA barcode markers. The first is search‐based using BLAST (barcode resolution) (e.g., Burgess et al., 2011), and the second one is tree‐based, which considers phylogenetic relationships (phylogenetic resolution, tree‐based) (e.g., Gonzalez et al., 2009), both with advantages and drawbacks (as discussed below). Therefore, we preferred to use both evaluation approaches.

At first glance, the barcode resolution may seem a more attractive approach, as noticeably higher values were obtained for the two best markers both individually and combined (*rbc*L—75.00%, ITS2—89.45%, and *rbc*L + ITS2—86.06%), when compared with the phylogenetic resolution (*rbc*L—62.30%, ITS2—75.16%, and *rbc*L + ITS2—66.02%). Moreover, using pairwise identity (or other related parameters of a BLAST search) to determine a correct sequence assignment (and consequently species identification) in DNA barcoding approaches is quite straightforward and practical, especially when handling a large volume of data. On the other hand, the importance of employing a parameter that reflects evolutionary relationships is obvious, as the inclusion of phylogenetic reconstructions with DNA barcoding data enables several other analytical inferences (Erickson et al., 2014; Kress, 2017; Kress et al., 2015; Miller et al., 2016). Therefore, besides assessing the levels of species discrimination of *rbc*L and ITS2 when barcoding the FCC, we also observed indicatives of complex evolutionary relationships either among or within populations of several endemic taxa (as described by Giulietti et al., 2019). In the cases of *Mimosa skinneri* var. *carajarum* Barneby (Fabaceae) and *Borreria elaiosulcata* E. L. Cabral & L. M. Miguel (Rubiaceae), for instance, the trees presented conflicting phylogenetic signals, as nonmonophyletic groupings and low bootstrap support, were observed (Supplementary Figures S1–S6).

Furthermore, the discrimination levels obtained for both markers (separately and combined) were in accordance with previous results for *rbc*L and ITS2 (e.g., Burgess et al., 2011; Kress et al., 2009; Parmentier et al., 2013), although relatively higher than observed for other diverse floras, as reported by Gonzalez et al. (2009) and Liu et al. (2015). The fact that both species discrimination approaches used here were overly sensitive to sampling coverage is noteworthy, as the analyses considering only specimens with both barcodes provided higher resolution values. This difference was especially strong in the case of the phylogenetic resolution of the combination *rbc*L + ITS2, with an increase of 20.95% in the proportion of resolved species in the reduced sampling in comparison with the complete sampling (from 66.02% to 79.85%; Table 2). Such difference occurred due to the exclusion of specimens from species and/or genera that present either more complex evolutionary histories or problematic taxonomy.

### 4.3 DNA barcodes and conservation

Biodiversity indexes provided by DNA barcoding data have an undeniably important role in better directing conservation efforts, as the effectiveness of maintaining ecological services of biodiversity hotspots can be greatly enhanced by including phylogenetic diversity parameters in the decision‐making process (Diniz et al., 2021; Forest et al., 2007). However, as mentioned before and pointed out by Kress (2017), properly populating the public databases with plant DNA barcodes has not been an easy task, being “one of the biggest challenges for the next decade”. The difficulties in achieving such an important goal are especially evidenced by considering the actions needed to ensure proper conservation planning in such an immense (and still poorly known) area as the Amazon basin. Hence, the data presented here are strategic as the first and only genetic data available for several plant species of the region. In addition, it is essential to pay extra attention to endemic and/or rare species of such a unique Amazon vegetation as the *campo rupestre* on *canga* of the Serra dos Carajás, as in the case of the morning‐glory *Ipomoea cavalcantei* and the quillwort *Isoetes cangae*, for instance. Both species present a very limited geographic distribution in the *canga* (Giulietti et al., 2019), with studies based on DNA barcoding data investigating their genetic diversity status for the first time (Babiychuk et al., 2017; Nunes et al., 2018), followed by further populational analyses (Babiychuk et al., 2019; Dalapicolla et al., 2021; Lanes et al., 2018).

Considering the list of endemic plants of the *canga* of the Serra dos Carajás, we obtained barcodes for 30 out of the 38 species listed by Giulietti et al. (2019). From the eight endemic species without a DNA barcode, we had access to tissue samples of only two specimens of *Pleroma carajasense* (Melastomataceae), for which we could not obtain DNA sequences of any of the tested markers, as occurred for 47 other samples of 36 species. Likewise, Burgess et al. (2011) had already observed that high‐throughput DNA isolation procedures would not always work with samples from a wide range of taxonomic groups, with some taxa frequently being more problematic than others, depending on the adopted protocols. The group with the worst performance within our sampling universe was, by far, Melastomataceae, for which we were able to generate sequences only for 26.32% of sampled specimens (and 30.77% of the species). It is important to mention that the Melastomataceae was recorded as the fifth most diverse angiosperm family in the FCC, with a total of 41 species (Mota et al., 2018; Rocha et al., 2017). Lima et al. (2018) also reported low rates of amplification success for *rbc*L and *mat*K when barcoding tree species of Melastomataceae, one of the most species‐rich families in the flora of the Brazilian state of São Paulo. Although these authors could overcome such a problem with the plastid markers by using the ITS region, the results we obtained here for the four barcoded species of Melastomataceae with ITS2 were only slightly better than for *rbc*L (one barcoded species), considering the universal protocols used. Thus, we acknowledge the crucial need for developing more directed protocols aiming at problematic taxa, which will be our next step toward accomplishing a DNA barcode library with full coverage for the flora of the Amazonian *canga*.

As mentioned above, inventorying species through DNA‐based tools has consistently gained ground along the years, achieving further importance with the development of multispecies identification approaches based on high‐throughput sequencing technologies (Deiner et al., 2017; Kress et al., 2015). Several authors have pointed out the many advantages of using DNA metabarcoding for monitoring biodiversity, especially considering robustness and efficiency of this analytical system (Bush et al., 2020; Deiner et al., 2017; Zinger et al., 2019). Certainly, the effectiveness of metabarcoding can be greatly affected depending on the completeness level of the reference DNA barcode library (Alsos et al., 2018); thus, care must be taken for its use for identification of specimens at species level until a complete barcode library is available, especially in areas with several narrowly distributed endemics. Nevertheless, the results obtained here for the bulk samples from Serra dos Carajás were very promising, as we could observe a relatively high taxonomic diversity within and among the collection sites, even with a coverage of less than one‐third (30.75%) of the *canga* species with ITS2 barcodes. Thus, the validity of DNA metabarcoding with ITS2 for monitoring plant species in Serra dos Carajás was successfully demonstrated, despite having a yet incomplete DNA barcode library.

### 4.4 Concluding remarks

Despite that DNA barcoding methods are well‐established for plant species, and thus the approach novelty is limited, our study brings a considerable amount of novel sequencing data for a unique flora within the Amazon basin, which still presents poorly characterized genetic resources. Furthermore, the value of DNA barcoding data to guide conservation efforts in the Serra dos Carajás has been demonstrated also in the ecological context by helping to identify the importance of some plant taxa acting as nutrient providers for animal communities in ferruginous caves (Ramalho et al., 2018).

While the more polymorphic nature of the marker ITS2 makes it more suitable for species identification in most cases of the genera with more than one species in the *canga* of the Serra dos Carajás, there were some cases in which *rbc*L was better for discriminating species, such as within the genus *Neea* (Nyctaginaceae). Besides, there is excellent species coverage with *rbc*L in the available DNA barcode libraries, being especially crucial in the cases of species without any genetic information available. Therefore, the importance of *rbc*L as a plant barcode marker is unquestionable, and our choice of implementing ITS2 together with *rbc*L as primary barcodes for the highly diverse flora of the Serra dos Carajás covers all three principles of DNA barcoding.

In the case of the metabarcoding analysis, our goal was to test the method's viability when studying the diverse flora of the Amazon ironstone fields, aiming to establish a starting point and basal parameters for future large‐scale studies in the region, using both bulk sampling and environmental DNA (eDNA) approaches (Oliveira et al., 2019). Hence, the ongoing development of the DNA barcode libraries for the region will be essential for the optimization of reforestation in decommissioned mining sites in the region, as well as fast and robust vegetation surveys in untouched native areas.

## CONFLICT OF INTEREST

The authors declare no conflict of interest.

## AUTHOR CONTRIBUTIONS

**Santelmo Vasconcelos:** Conceptualization (equal); Data curation (equal); Formal analysis (lead); Investigation (lead); Methodology (lead); Project administration (supporting); Resources (equal); Software (supporting); Validation (lead); Visualization (lead); Writing‐original draft (lead); Writing‐review & editing (lead). **Gisele L. Nunes:** Conceptualization (supporting); Data curation (equal); Formal analysis (equal); Investigation (supporting); Methodology (supporting); Project administration (supporting); Resources (equal); Software (equal); Validation (supporting); Visualization (supporting); Writing‐original draft (supporting); Writing‐review & editing (supporting). **Mariana C. Dias:** Data curation (supporting); Formal analysis (supporting); Investigation (supporting); Methodology (supporting); Resources (equal); Validation (equal); Writing‐review & editing (supporting). **Jamily Lorena:** Formal analysis (supporting); Investigation (supporting); Methodology (supporting); Resources (supporting); Validation (supporting); Writing‐review & editing (supporting). **Renato R. M. Oliveira:** Data curation (equal); Formal analysis (supporting); Investigation (supporting); Methodology (supporting); Resources (supporting); Software (equal); Validation (supporting); Writing‐review & editing (supporting). **Talvâne G. L. Lima:** Data curation (supporting); Formal analysis (supporting); Methodology (supporting); Software (equal). **Eder S. Pires:** Formal analysis (supporting); Investigation (supporting); Methodology (supporting); Resources (supporting). **Rafael B. S. Valadares:** Investigation (supporting); Methodology (supporting); Resources (supporting); Writing‐review & editing (supporting). **Ronnie Alves:** Data curation (supporting); Methodology (supporting); Resources (supporting); Software (supporting); Writing‐review & editing (supporting). **Maurício T. C. Watanabe:** Investigation (supporting); Methodology (supporting); Resources (supporting); Writing‐review & editing (supporting). **Daniela C. Zappi:** Data curation (supporting); Investigation (supporting); Methodology (supporting); Resources (supporting); Validation (supporting); Writing‐review & editing (supporting). **Alice L. Hiura:** Data curation (supporting); Methodology (supporting); Resources (supporting); Validation (supporting). **Mayara Pastore:** Data curation (supporting); Investigation (supporting); Methodology (supporting); Resources (supporting); Validation (supporting); Writing‐review & editing (supporting). **Liziane V. Vasconcelos:** Data curation (supporting); Investigation (supporting); Methodology (supporting); Resources (supporting); Writing‐review & editing (supporting). **Nara F. O. Mota:** Data curation (supporting); Investigation (supporting); Methodology (supporting); Resources (supporting); Validation (supporting); Writing‐review & editing (supporting). **Pedro L. Viana:** Conceptualization (equal); Data curation (supporting); Funding acquisition (supporting); Investigation (supporting); Methodology (supporting); Project administration (supporting); Resources (equal); Validation (supporting); Writing‐review & editing (supporting). **André S. B. Gil:** Data curation (supporting); Investigation (supporting); Resources (supporting); Validation (supporting); Writing‐review & editing (supporting). **Andre O. Simões:** Investigation (supporting); Methodology (supporting); Resources (supporting); Writing‐review & editing (supporting). **Vera L. Imperatriz‐Fonseca:** Conceptualization (supporting); Funding acquisition (equal); Investigation (supporting); Project administration (supporting); Resources (supporting); Supervision (supporting); Writing‐review & editing (supporting). **Raymond M. Harley:** Conceptualization (supporting); Data curation (supporting); Investigation (supporting); Methodology (supporting); Resources (supporting); Validation (supporting); Writing‐review & editing (supporting). **Ana M. Giulietti:** Conceptualization (equal); Data curation (supporting); Investigation (supporting); Methodology (supporting); Project administration (supporting); Resources (equal); Supervision (supporting); Validation (supporting); Writing‐review & editing (supporting). **Guilherme Oliveira:** Conceptualization (equal); Data curation (equal); Funding acquisition (lead); Investigation (supporting); Methodology (supporting); Project administration (lead); Resources (equal); Software (supporting); Supervision (lead); Validation (supporting); Writing‐review & editing (supporting).

### OPEN RESEARCH BADGES

This article has earned an Open Data Badge for making publicly available the digitally‐shareable data necessary to reproduce the reported results. The data is available at https://doi.org/10.17605/osf.io/5xt3u.

## Supporting information

Table A1‐A4

## Data Availability

All DNA sequences generated for this work may be accessed through the BOLD accession numbers indicated in the Table A1. In addition, all Supplementary Data (Alignments, Figures S01–S13 and Tables S1–S4) are available in the Open Science Framework repository (https://doi.org/10.17605/osf.io/5xt3u). All other data will be made available upon request.
