# Supplementary material for: Unraveling the plant diversity of the Amazonian canga through DNA barcoding
Source: Ecol Evol. 2021 Aug 31;11(19):13348–62. doi: 10.1002/ece3.8057 (PMC8495817; doi:10.1002/ece3.8057)
Supplement: Supplementary file 1 — Table A1‐A4 [file ECE3-11-13348-s001.docx]

**APPENDIX**

**Table A1.** List of all samples used in the DNA barcoding of canga plants of the Serra dos Carajás and other related regions, bringing taxonomic information, BOLD accessions, voucher numbers, presence in the species list of the Flora of the canga of Carajás (LFCC), as described in Mota et al. (2018), whether the sample was included in the test with the eight different markers (MT), presence of previously published DNA barcode for the species in the BOLD database (FDB), and sequenced markers per accession. The accessions without any generated sequence are listed in the Supplementary Table S1 (https://osf.io/5xt3u/).

| **Order** | **Family** | **Species** | **BOLD accession** | **Voucher** | **LFCC** | **MT^b^** | **FDB^c^** | **Sequenced markers** |
| --- | --- | --- | --- | --- | --- | --- | --- | --- |
| Alismatales | Alismataceae | *Helanthium tenellum* (Mart.) Britton | CANGA418-17 | Harley R.M. 57309 | Yes | Yes | Yes | rbcL, ITS2, rpoC1, atpF-atpH, psbK-psbI |
| Alismatales | Alismataceae | *Helanthium tenellum* (Mart.) Britton | CANGI009-17 | Trindade J.R. 220 | Yes | Yes | Yes | ITS2, rpoB, rpoC1, atpF-atpH, psbK-psbI |
| Alismatales | Alismataceae | *Helanthium tenellum* (Mart.) Britton | CANGA419-17 | Harley R.M. 57350 | Yes | Yes | Yes | rbcL, ITS2, matK, rpoC1, atpF-atpH, psbK-psbI, trnH-psbA |
| Alismatales | Alismataceae | *Helanthium tenellum* (Mart.) Britton | CANGI011-17 | Pastore M. 613 | Yes | No | Yes | rbcL, ITS2 |
| Alismatales | Alismataceae | *Limnocharis laforestii* Duchass. ex Griseb. | CANGI007-17 | Pastore M. 555 | No | No | No | rbcL, ITS2 |
| Alismatales | Alismataceae | *Limnocharis laforestii* Duchass. ex Griseb. | CANGI008-17 | Pastore M. 557 | No | No | No | rbcL, ITS2 |
| Alismatales | Alismataceae | *Sagittaria guayanensis* Kunth | CANGI012-17 | Pastore M. 556 | No | No | No | rbcL, ITS2 |
| Alismatales | Araceae | *Anthurium bonplandii* Bunting | CANGI038-17 | Vasconcelos L.V. 1072 | Yes | No | Yes | rbcL |
| Alismatales | Araceae | *Anthurium clavigerum* Poepp. | CANGA148-17 | Vasconcelos L.V. 888 | No | Yes | Yes | rbcL, trnH-psbA |
| Alismatales | Araceae | *Anthurium lindmanianum* Engl. | CANGA264-17 | N. Mota 3404 | Yes | Yes | Yes | rbcL, rpoB, rpoC1 |
| Alismatales | Araceae | *Anthurium lindmanianum* Engl. | CANGA265-17 | Lopes C.S.A. 7 | Yes | Yes | Yes | rbcL, rpoB, rpoC1, atpF-atpH, psbK-psbI |
| Alismatales | Araceae | *Anthurium sinuatum* Benth. ex Schott | CANGI041-17 | Vasconcelos L.V. 1077 | Yes | No | Yes | rbcL |
| Alismatales | Araceae | *Anthurium* sp.1 | CANGI037-17 | Viana P.L. 6211 | Yes | Yes | Yes | rbcL |
| Alismatales | Araceae | *Anthurium* sp.1 | CANGI039-17 | Harley R.M. 58109 | Yes | No | Yes | rbcL |
| Alismatales | Araceae | *Philodendron acutatum* Schott | CANGA369-17 | Vasconcelos L.V. 887 | No | Yes | Yes | rbcL |
| Alismatales | Araceae | *Philodendron acutatum* Schott | CGII631-20 | Zappi D.C. 4356 | No | No | Yes | rbcL |
| Alismatales | Araceae | *Philodendron blanchetianum* Schott | CANGA001-17 | Vasconcelos L.V. 764 | Yes | Yes | Yes | rbcL |
| Alismatales | Araceae | *Philodendron carajasense* E.G. Gonç. & A.J. Arruda | CANGA125-17 | Vasconcelos L.V. 906 | Yes | No | Yes | rbcL, ITS2 |
| Alismatales | Araceae | *Philodendron quinquelobum* K.Krause | CANGA368-17 | Vasconcelos L.V. 886 | No | Yes | Yes | rbcL, ITS2 |
| Alismatales | Araceae | *Philodendron wullschlaegelii* Schott | CANGA434-17 | Vasconcelos L.V. 782 | Yes | Yes | Yes | rbcL |
| Alismatales | Araceae | *Spathiphyllum gardneri* Schott | CGII651-20 | Zappi D.C. 4478 | Yes | No | Yes | ITS2 |
| Arecales | Arecaceae | *Syagrus cocoides* Mart. | CANGI042-17 | Viana P.L. 6215 | Yes | Yes | Yes | rbcL, ITS2 |
| Arecales | Arecaceae | *Syagrus cocoides* Mart. | CANGI043-17 | Nogueira M.G.C. 745 | Yes | No | Yes | rbcL |
| Arecales | Arecaceae | *Syagrus comosa* (Mart.) Mart. | CGII172-20 | Zappi D.C. 4486 | No | No | Yes | rbcL |
| Asparagales | Iridaceae | *Cipura xanthomelas* Klatt | CANGA437-17 | Vasconcelos L.V. 1084 | Yes | No | Yes | rbcL, ITS2 |
| Asparagales | Orchidaceae | *Campylocentrum fasciola* (Lindl.) Cogn. | CANGI397-17 | Gil A. 510 | Yes | Yes | No | rbcL, ITS2 |
| Asparagales | Orchidaceae | *Campylocentrum fasciola* (Lindl.) Cogn. | CANGI398-17 | Harley R.M. 57249 | Yes | Yes | No | ITS2, matK, rpoB, atpF-atpH |
| Asparagales | Orchidaceae | *Catasetum discolor* (Lindl.) Lindl. | CANGI406-17 | Meirelles J. 930 | Yes | Yes | Yes | rbcL, ITS2, matK, rpoB, rpoC1, psbK-psbI, trnH-psbA |
| Asparagales | Orchidaceae | *Catasetum discolor* (Lindl.) Lindl. | CANGI399-17 | Viana P.L. 6146 | Yes | Yes | Yes | rbcL, ITS2 |
| Asparagales | Orchidaceae | *Cyrtopodium andersonii* (Lamb. ex Andrews) R.Br. | CANGI401-17 | Harley R.M. 57924 | Yes | No | No | rbcL |
| Asparagales | Orchidaceae | *Cyrtopodium cachimboense* L.C.Menezes | CANGA046-17 | Vasconcelos L.V. 915 | No | No | Yes | rbcL |
| Asparagales | Orchidaceae | *Encyclia randii* (Barb.Rodr.) Porto & Brade | CANGA372-17 | Harley R.M. 57344 | Yes | Yes | No | rbcL, ITS2, matK, atpF-atpH |
| Asparagales | Orchidaceae | *Epidendrum nocturnum* Jacq. | CANGI407-17 | Harley R.M. 57450 | Yes | Yes | No | rbcL, ITS2 |
| Asparagales | Orchidaceae | *Epidendrum nocturnum* Jacq. | CANGA318-17 | Harley R.M. 58144 | Yes | No | No | rbcL, ITS2 |
| Asparagales | Orchidaceae | *Epidendrum purpurascens* Focke | CANGA044-17 | Dias C.S.P. 9 | Yes | Yes | Yes | rbcL, matK, rpoC1 |
| Asparagales | Orchidaceae | *Epidendrum purpurascens* Focke | CANGI408-17 | Vasconcelos L.V. 1039 | Yes | No | Yes | rbcL, ITS2 |
| Asparagales | Orchidaceae | *Habenaria* aff. *nuda* Lindl. | CANGI402-17 | Viana P.L. 6134 | Yes | Yes | No | rbcL, ITS2 |
| Asparagales | Orchidaceae | *Scaphyglottis stellata* Lodd. ex Lindl. | CANGI404-17 | Viana P.L. 6216 | Yes | Yes | No | rbcL, ITS2 |
| Asparagales | Orchidaceae | *Sobralia liliastrum* Salzm. ex Lindl. | CANGI405-17 | Reis A.S. 22 | Yes | Yes | Yes | rbcL, ITS2, trnH-psbA |
| Asparagales | Orchidaceae | *Sobralia liliastrum* Salzm. ex Lindl. | CANGA262-17 | Viana P.L. 6101 | Yes | Yes | Yes | rbcL, ITS2 |
| Asparagales | Orchidaceae | *Sobralia liliastrum* Salzm. ex Lindl. | CANGA263-17 | Pastore M. 365 | Yes | Yes | Yes | rbcL, ITS2, trnH-psbA |
| Asparagales | Orchidaceae | *Trichocentrum sprucei* (Lindl.) M.W.Chase & N.H.Williams | CANGA121-17 | Mota N.F.O. 3435 | Yes | Yes | Yes | rbcL, ITS2, matK |
| Asterales | Asteraceae | *Aspilia attenuata* (Gardner) Baker | CANGA027-17 | Vasconcelos L.V. 798 | Yes | Yes | Yes | rbcL, ITS2, trnH-psbA |
| Asterales | Asteraceae | *Bidens* sp. | CGII632-20 | Zappi D.C. 4359 | No | No | Yes | rbcL, ITS2 |
| Asterales | Asteraceae | *Cavalcantia percymosa* R.M.King & H.Rob | CANGA347-17 | Cruz A.P. 38 | Yes | Yes | Yes | rbcL, ITS2, matK, atpF-atpH, psbK-psbI, trnH-psbA |
| Asterales | Asteraceae | *Cavalcantia percymosa* R.M.King & H.Rob | CANGA348-17 | Viana P.L. 6179 | Yes | Yes | Yes | rbcL, ITS2, trnH-psbA |
| Asterales | Asteraceae | *Chromolaena maximilianii* (Schrad. ex DC.) R.M.King & H.Rob. | CANGA281-17 | Vasconcelos L.V. 1056 | Yes | No | Yes | rbcL, ITS2 |
| Asterales | Asteraceae | *Eclipta prostrata* (L.) L. | CANGA358-17 | Cruz A.P. 30 | Yes | Yes | No | rbcL, ITS2, matK, rpoB, atpF-atpH |
| Asterales | Asteraceae | *Erechtites hieraciifolius* (L.) Raf. ex DC. | CANGI059-17 | Harley R.M. 58138 | Yes | No | No | rbcL, ITS2 |
| Asterales | Asteraceae | *Ichthyothere terminalis* (Spreng.) S.F.Blake | CANGA421-17 | Cruz A.P. 35 | Yes | Yes | No | rbcL, ITS2, matK |
| Asterales | Asteraceae | *Ichthyothere terminalis* (Spreng.) S.F.Blake | CANGI056-17 | Praia T.S. 24 | Yes | Yes | No | rbcL, rpoC1, atpF-atpH |
| Asterales | Asteraceae | *Ichthyothere terminalis* (Spreng.) S.F.Blake | CANGA422-17 | Viana P.L. 6175 | Yes | Yes | No | rbcL |
| Asterales | Asteraceae | *Ichthyothere terminalis* (Spreng.) S.F.Blake | CANGA470-20 | Giulietti A.M. 2664 | Yes | No | No | ITS2 |
| Asterales | Asteraceae | *Ichthyothere terminalis* (Spreng.) S.F.Blake | CGII619-20 | Zappi D.C. 4314 | Yes | No | No | ITS2 |
| Asterales | Asteraceae | *Lepidaploa arenaria* (Mart. ex DC.) H.Rob. | CANGA023-17 | Vasconcelos L.V. 791 | Yes | Yes | Yes | rbcL, ITS2 |
| Asterales | Asteraceae | *Lepidaploa arenaria* (Mart. ex DC.) H.Rob. | CANGA024-17 | Vasconcelos L.V. 1064 | Yes | No | Yes | rbcL, ITS2 |
| Asterales | Asteraceae | *Lepidaploa paraensis* (H.Rob.) H.Rob. | CANGA331-17 | Vasconcelos L.V. 847 | Yes | Yes | Yes | rbcL, ITS2 |
| Asterales | Asteraceae | *Lepidaploa remotiflora* (Rich.) H.Rob. | CANGA374-17 | Vasconcelos L.V. 790 | Yes | Yes | Yes | rbcL, ITS2 |
| Asterales | Asteraceae | *Lepidaploa remotiflora* (Rich.) H.Rob. | CANGA375-17 | Vasconcelos L.V. 812 | Yes | Yes | Yes | rbcL, ITS2 |
| Asterales | Asteraceae | *Lepidaploa remotiflora* (Rich.) H.Rob. | CANGI055-17 | Harley R.M. 58071 | Yes | No | Yes | rbcL, ITS2 |
| Asterales | Asteraceae | *Lepidaploa remotiflora* (Rich.) H.Rob. | CANGI060-17 | Pastore M. 599 | Yes | No | Yes | rbcL, ITS2 |
| Asterales | Asteraceae | *Monogereion carajensis* G.M. Barroso & R.M. King | CANGA091-17 | Vasconcelos L.V. 766 | Yes | Yes | Yes | rbcL, ITS2, trnH-psbA |
| Asterales | Asteraceae | *Monogereion carajensis* G.M. Barroso & R.M. King | CANGA092-17 | Vasconcelos L.V. 766 | Yes | Yes | Yes | rbcL, ITS2, trnH-psbA |
| Asterales | Asteraceae | *Monogereion carajensis* G.M. Barroso & R.M. King | CANGA097-17 | Viana P.L. 6113 | Yes | Yes | Yes | rbcL, ITS2 |
| Asterales | Asteraceae | *Monogereion carajensis* G.M. Barroso & R.M. King | CANGA098-17 | Pastore M. 633 | Yes | No | Yes | rbcL, ITS2 |
| Asterales | Asteraceae | *Monogereion carajensis* G.M. Barroso & R.M. King | CANGI050-17 | Harley R.M. 57907 | Yes | Yes | Yes | rbcL, ITS2 |
| Asterales | Asteraceae | *Monogereion carajensis* G.M. Barroso & R.M. King | CANGA093-17 | Vasconcelos L.V. 1086 | Yes | No | Yes | rbcL, ITS2 |
| Asterales | Asteraceae | *Monogereion carajensis* G.M. Barroso & R.M. King | CANGA094-17 | Vasconcelos L.V. 1137 | Yes | No | Yes | rbcL, ITS2 |
| Asterales | Asteraceae | *Monogereion carajensis* G.M. Barroso & R.M. King | CANGA095-17 | Vasconcelos L.V. 1142 | Yes | No | Yes | rbcL, ITS2 |
| Asterales | Asteraceae | *Monogereion carajensis* G.M. Barroso & R.M. King | CANGI051-17 | Harley R.M. 58094 | Yes | No | Yes | rbcL, ITS2 |
| Asterales | Asteraceae | *Parapiqueria cavalcantei* R.M.King & H.Rob. | CANGA117-17 | Cruz A.P. 1 | Yes | Yes | Yes | rbcL, ITS2, matK |
| Asterales | Asteraceae | *Praxelis asperulacea* R.M.King & H.Rob. | CANGI049-17 | Pastore M. 586 | Yes | No | Yes | rbcL, ITS2 |
| Asterales | Asteraceae | *Pterocaulon alopecuroides* (Lam.) DC. | CANGI052-17 | Harley R.M. 58051 | Yes | No | Yes | rbcL, ITS2 |
| Asterales | Asteraceae | *Riencourtia pedunculosa* (Rich.) Pruski | CANGA341-17 | Vasconcelos L.V. 797 | Yes | Yes | Yes | rbcL, ITS2 |
| Asterales | Asteraceae | *Riencourtia pedunculosa* (Rich.) Pruski | CANGA342-17 | Vasconcelos L.V. 848 | Yes | Yes | Yes | rbcL, ITS2 |
| Asterales | Asteraceae | *Riencourtia pedunculosa* (Rich.) Pruski | CANGA346-17 | Vasconcelos L.V. 879 | Yes | Yes | Yes | rbcL, ITS2 |
| Asterales | Asteraceae | *Riencourtia pedunculosa* (Rich.) Pruski | CANGA343-17 | Viana P.L. 6129 | Yes | Yes | Yes | rbcL, ITS2 |
| Asterales | Asteraceae | *Riencourtia pedunculosa* (Rich.) Pruski | CANGA344-17 | Viana P.L. 6230 | Yes | Yes | Yes | rbcL, ITS2 |
| Asterales | Asteraceae | *Riencourtia pedunculosa* (Rich.) Pruski | CANGA345-17 | Pastore M. 614 | Yes | No | Yes | rbcL, ITS2 |
| Asterales | Asteraceae | *Riencourtia pedunculosa* (Rich.) Pruski | CANGI053-17 | Nogueira M.G.C. 654 | Yes | No | Yes | rbcL, ITS2 |
| Asterales | Asteraceae | *Riencourtia pedunculosa* (Rich.) Pruski | CANGI054-17 | Harley R.M. 58087 | Yes | No | Yes | rbcL, ITS2 |
| Asterales | Asteraceae | *Riencourtia pedunculosa* (Rich.) Pruski | CGII615-20 | Zappi D.C. 4297 | Yes | No | Yes | rbcL, ITS2 |
| Asterales | Asteraceae | *Rolandra fruticosa* (L.) Kuntze | CANGA205-17 | Gil A. 519 | Yes | Yes | No | ITS2, matK |
| Asterales | Asteraceae | *Tilesia baccata* (L.f.) Pruski | CANGI058-17 | Pastore M. 577 | Yes | No | No | rbcL, ITS2 |
| Asterales | Menyanthaceae | *Nymphoides humboldtiana* (Kunth) Kuntze | CANGA234-17 | Harley R.M. 57305 | Yes | Yes | No | ITS2, matK, rpoB, psbK-psbI |
| Boraginales | Cordiaceae | *Cordia exaltata* Lam. | CANGI116-17 | Harley R.M. 57953 | Yes | Yes | Yes | rbcL, ITS2 |
| Boraginales | Cordiaceae | *Varronia polycephala* Lam. | CANGI083-17 | Nogueira M.G.C. 662 | No | No | No | rbcL, ITS2 |
| Caryophyllales | Amaranthaceae | *Alternanthera dentata* (Moench) Stuchlík ex R.E.Fr. | CANGI013-17 | Vasconcelos L.V. 902 | Yes | No | Yes | rbcL |
| Caryophyllales | Amaranthaceae | *Alternanthera tenella* Colla | CGII633-20 | Zappi D.C. 4360 | Yes | No | No | rbcL, ITS2 |
| Caryophyllales | Amaranthaceae | *Gomphrena arborescens* L.f. | ITVGA002-17 | Nogueira M.G.C. 421 | No | Yes | Yes | rbcL, ITS2, matK, rpoB, atpF-atpH, psbK-psbI |
| Caryophyllales | Cactaceae | *Cereus hexagonus* (L.) Mill. | CANGI097-17 | Dias C.S.P. 14 | Yes | Yes | Yes | rbcL, rpoB, rpoC1, atpF-atpH, psbK-psbI |
| Caryophyllales | Cactaceae | *Cereus hexagonus* (L.) Mill. | CANGI094-17 | Vasconcelos L.V. 1103 | Yes | No | Yes | rbcL, ITS2 |
| Caryophyllales | Cactaceae | *Cereus hexagonus* (L.) Mill. | CGII655-20 | Zappi D.C. 4548 | Yes | No | Yes | rbcL, ITS2 |
| Caryophyllales | Cactaceae | *Epiphyllum phyllanthus* (L.) Haw. | CANGI095-17 | Vasconcelos L.V. 1108 | Yes | No | No | rbcL, ITS2 |
| Caryophyllales | Caryophyllaceae | *Polycarpaea corymbosa* (L.) Lam. | CANGI098-17 | Viana P.L. 6212 | No | Yes | Yes | rbcL, ITS2 |
| Caryophyllales | Molluginaceae | *Mollugo verticillata* L. | CANGI357-17 | Giulietti A.M. 2657 | No | No | No | rbcL, ITS2 |
| Caryophyllales | Nyctaginaceae | *Neea floribunda* Poepp. & Endl. | CANGI374-17 | Harley R.M. 57321 | Yes | Yes | No | rbcL, ITS2, matK, rpoC1, psbK-psbI |
| Caryophyllales | Nyctaginaceae | *Neea macrophylla* Poepp. & Endl. | CANGI373-17 | Mota N.F.O. 3387 | Yes | Yes | Yes | ITS2, matK, rpoB, rpoC1 |
| Caryophyllales | Nyctaginaceae | *Neea macrophylla* Poepp. & Endl. | CANGI365-17 | Harley R.M. 57336 | Yes | Yes | Yes | rbcL, ITS2, matK, rpoB, rpoC1, atpF-atpH |
| Caryophyllales | Nyctaginaceae | *Neea macrophylla* Poepp. & Endl. | CANGI367-17 | Viana P.L. 5758 | Yes | Yes | Yes | ITS2, matK, rpoC1, trnH-psbA |
| Caryophyllales | Nyctaginaceae | *Neea macrophylla* Poepp. & Endl. | CANGA270-17 | Trindade J.R. 358 | Yes | Yes | Yes | ITS2, matK, rpoB, rpoC1, atpF-atpH |
| Caryophyllales | Nyctaginaceae | *Neea macrophylla* Poepp. & Endl. | CANGI375-17 | Lopes C.S.A. 3 | Yes | Yes | Yes | ITS2, |
| Caryophyllales | Nyctaginaceae | *Neea oppositifolia* Ruiz & Pav. | CANGI366-17 | Harley R.M. 57351 | Yes | Yes | Yes | rbcL, ITS2, matK |
| Caryophyllales | Nyctaginaceae | *Neea oppositifolia* Ruiz & Pav. | CANGI368-17 | Gil A. 516 | Yes | Yes | Yes | rbcL, atpF-atpH |
| Caryophyllales | Nyctaginaceae | *Neea oppositifolia* Ruiz & Pav. | CANGI369-17 | Trindade J.R. 377 | Yes | Yes | Yes | rbcL, ITS2, matK, rpoC1 |
| Caryophyllales | Nyctaginaceae | *Neea oppositifolia* Ruiz & Pav. | CANGA134-17 | Trindade J.R. 378 | Yes | Yes | Yes | ITS2, matK, rpoB |
| Caryophyllales | Nyctaginaceae | *Neea oppositifolia* Ruiz & Pav. | CANGI376-17 | Praia T.S. 20 | Yes | Yes | Yes | rbcL, ITS2, psbK-psbI |
| Caryophyllales | Nyctaginaceae | *Neea oppositifolia* Ruiz & Pav. | CANGI371-17 | Harley R.M. 57905 | Yes | Yes | Yes | rbcL, ITS2 |
| Caryophyllales | Nyctaginaceae | *Neea oppositifolia* Ruiz & Pav. | CANGA323-17 | Vasconcelos L.V. 1025 | Yes | No | Yes | rbcL, ITS2 |
| Caryophyllales | Nyctaginaceae | *Neea oppositifolia* Ruiz & Pav. | CANGI372-17 | Vasconcelos L.V. 1031 | Yes | No | Yes | rbcL, ITS2 |
| Caryophyllales | Polygonaceae | *Polygonum acuminatum* Kunth | CANGI490-17 | Trindade J.R. 226 | Yes | Yes | Yes | rbcL, ITS2, matK, rpoB, rpoC1, atpF-atpH |
| Caryophyllales | Portulacaceae | *Portulaca sedifolia* N.E.Br. | CANGI495-17 | Cardoso A. 1952 | Yes | Yes | Yes | rbcL, ITS2, matK, rpoB, rpoC1, atpF-atpH |
| Caryophyllales | Portulacaceae | *Portulaca sedifolia* N.E.Br. | CANGI505-17 | Reis A.S. 39 | Yes | Yes | Yes | rbcL, ITS2, psbK-psbI |
| Caryophyllales | Portulacaceae | *Portulaca sedifolia* N.E.Br. | CANGI497-17 | Viana P.L. 6131 | Yes | Yes | Yes | rbcL, ITS2 |
| Caryophyllales | Portulacaceae | *Portulaca sedifolia* N.E.Br. | CANGI498-17 | Viana P.L. 6177 | Yes | Yes | Yes | rbcL, ITS2 |
| Caryophyllales | Portulacaceae | *Portulaca sedifolia* N.E.Br. | CANGI499-17 | Viana P.L. 6223 | Yes | Yes | Yes | rbcL, ITS2 |
| Caryophyllales | Portulacaceae | *Portulaca sedifolia* N.E.Br. | CANGI500-17 | Harley R.M. 58153 | Yes | No | Yes | rbcL, ITS2 |
| Caryophyllales | Portulacaceae | *Portulaca sedifolia* N.E.Br. | CANGI501-17 | Pastore M. 670 | Yes | No | Yes | rbcL, ITS2 |
| Caryophyllales | Portulacaceae | *Portulaca sedifolia* N.E.Br. | CANGI502-17 | Harley R.M. 57495 | Yes | Yes | Yes | rbcL, ITS2 |
| Caryophyllales | Portulacaceae | *Portulaca sedifolia* N.E.Br. | CANGI503-17 | Pastore M. 593 | Yes | No | Yes | rbcL, ITS2 |
| Caryophyllales | Rhabdodendraceae | *Rhabdodendron amazonicum* (Spruce ex Benth.) Huber | CANGA474-20 | Giulietti A.M. 2624 | No | No | No | ITS2 |
| Celastrales | Celastraceae | *Anthodon decussatus* Ruiz & Pav. | CANGI099-17 | Giulietti A.M. 2612 | Yes | No | Yes | rbcL, ITS2 |
| Celastrales | Celastraceae | *Anthodon decussatus* Ruiz & Pav. | CANGI100-17 | Vasconcelos L.V. 1070 | Yes | No | Yes | rbcL, ITS2 |
| Commelinales | Commelinaceae | *Dichorisandra hexandra* (Aubl.) C.B.Clarke | CANGA228-17 | Vasconcelos L.V. 1143 | Yes | No | Yes | rbcL |
| Commelinales | Commelinaceae | *Dichorisandra hexandra* (Aubl.) C.B.Clarke | CANGI111-17 | Pastore M. 545 | Yes | No | Yes | rbcL |
| Commelinales | Commelinaceae | *Dichorisandra hexandra* (Aubl.) C.B.Clarke | CANGI173-17 | Harley R.M. 58072 | Yes | No | Yes | rbcL |
| Commelinales | Commelinaceae | *Dichorisandra hexandra* Mart. ex Schult. f. | CANGI109-17 | Harley R.M. 58171 | Yes | No | Yes | rbcL |
| Commelinales | Commelinaceae | *Dichorisandra villosula* Mart. ex Schult. f. | CANGI110-17 | Pastore M. 525 | Yes | No | Yes | rbcL, ITS2 |
| Commelinales | Commelinaceae | *Murdannia nudiflora* (L.) Brenan | CANGI474-17 | Pastore M. 534 | Yes | No | No | rbcL, ITS2 |
| Cucurbitales | Cucurbitaceae | *Gurania bignoniacea* (Poepp. & Endl.) C.Jeffrey | CANGI127-17 | Trindade J.R. 230 | Yes | Yes | Yes | rbcL, ITS2, matK, rpoB, rpoC1, atpF-atpH |
| Cucurbitales | Cucurbitaceae | *Gurania insolita* Cogn. | CANGA239-17 | Harley R.M. 57991 | No | No | Yes | rbcL, ITS2 |
| Cucurbitales | Cucurbitaceae | *Gurania subumbellata* (Miq.) Cogn. | CANGI128-17 | Harley R.M. 57469 | Yes | Yes | Yes | rbcL |
| Cyatheales | Cyatheaceae | *Cyathea microdonta* (Desv.) Domin | CANGI129-17 | Meirelles J. 948 | Yes | Yes | Yes | rbcL |
| Cyatheales | Metaxyaceae | *Metaxya scalaris* Tuomisto & G.G. Cárdenas | CANGI177-17 | Harley R.M. 57943 | No | No | Yes | rbcL |
| Dioscoreales | Burmanniaceae | *Burmannia capitata* (Walter ex J.F.Gmel.) Mart. | CANGA055-17 | Harley R.M. 58123 | Yes | No | No | rbcL, ITS2 |
| Dioscoreales | Burmanniaceae | *Burmannia flava* Mart. | CANGA198-17 | Viana P.L. 6145 | Yes | Yes | Yes | rbcL, ITS2 |
| Dioscoreales | Burmanniaceae | *Burmannia flava* Mart. | CANGA199-17 | Meirelles J. 949 | Yes | Yes | Yes | rbcL, ITS2 |
| Dioscoreales | Burmanniaceae | *Burmannia flava* Mart. | CANGA197-17 | Pastore M. 581 | Yes | No | Yes | rbcL, ITS2 |
| Dioscoreales | Dioscoreaceae | *Dioscorea* cf. *melastomatifolia* Uline ex Prain | CANGI167-17 | Pastore M. 500 | No | No | Yes | rbcL, ITS2 |
| Dioscoreales | Dioscoreaceae | *Dioscorea* cf. *melastomatifolia* Uline ex Prain | CANGI168-17 | Pastore M. 501 | No | No | Yes | rbcL |
| Dioscoreales | Dioscoreaceae | *Dioscorea marginata* Griseb. | CANGI356-17 | Nogueira M.G.C. 658 | Yes | No | Yes | rbcL |
| Dioscoreales | Dioscoreaceae | *Dioscorea piperifolia* Humb. & Bonpl. ex Willd. | CANGI171-17 | Nogueira M.G.C. 660 | Yes | No | Yes | rbcL, ITS2 |
| Dioscoreales | Dioscoreaceae | *Dioscorea piperifolia* Humb. & Bonpl. ex Willd. | CANGI172-17 | Nogueira M.G.C. 661 | Yes | No | Yes | rbcL, ITS2 |
| Dioscoreales | Dioscoreaceae | *Dioscorea pohlii* Griseb. | CANGI174-17 | Harley R.M. 58081 | No | No | Yes | rbcL |
| Dioscoreales | Dioscoreaceae | *Dioscorea pohlii* Griseb. | CANGI175-17 | Harley R.M. 58082 | No | No | Yes | rbcL |
| Ericales | Marcgraviaceae | *Norantea guianensis* Aubl. | CANGA222-17 | Viana P.L. 5783 | Yes | Yes | No | rbcL, ITS2 |
| Ericales | Marcgraviaceae | *Norantea guianensis* Aubl. | CANGA223-17 | Reis A.S. 34 | Yes | Yes | No | rbcL, ITS2 |
| Ericales | Marcgraviaceae | *Norantea guianensis* Aubl. | CANGI340-17 | Giulietti A.M. ITV4119 | Yes | No | No | ITS2 |
| Ericales | Pentaphylacaceae | *Ternstroemia dentata* (Aubl.) Sw. | CGII173-20 | Zappi D.C. 4490 | No | No | No | rbcL, ITS2 |
| Ericales | Primulaceae | *Cybianthus detergens* Mart. | CANGI506-17 | Mota N.F.O. 3419 | Yes | Yes | Yes | ITS2, matK, rpoB |
| Ericales | Primulaceae | *Cybianthus detergens* Mart. | CANGA475-20 | Giulietti A.M. 2631 | Yes | No | Yes | rbcL, ITS2 |
| Ericales | Primulaceae | *Cybianthus detergens* Mart. | CANGI507-17 | Vasconcelos L.V. 1021 | Yes | No | Yes | rbcL, ITS2 |
| Ericales | Sapotaceae | *Pouteria ramiflora* (Mart.) Radlk. | CANGI572-17 | Mota N.F.O. 3388 | Yes | Yes | Yes | rbcL, matK, rpoB, rpoC1, atpF-atpH |
| Ericales | Sapotaceae | *Pouteria ramiflora* (Mart.) Radlk. | CANGI573-17 | Harley R.M. 57334 | Yes | Yes | Yes | rbcL, ITS2, matK, |
| Ericales | Sapotaceae | *Pouteria ramiflora* (Mart.) Radlk. | CANGI574-17 | Gil A. 522 | Yes | Yes | Yes | rbcL, ITS2, matK, atpF-atpH |
| Ericales | Sapotaceae | *Pouteria ramiflora* (Mart.) Radlk. | CANGI577-17 | Dias C.S.P. 7 | Yes | Yes | Yes | ITS2, rpoB |
| Ericales | Sapotaceae | *Pouteria ramiflora* (Mart.) Radlk. | CANGI578-17 | Harley R.M. 57996 | Yes | Yes | Yes | rbcL, ITS2 |
| Ericales | Sapotaceae | *Pouteria ramiflora* (Mart.) Radlk. | CANGI579-17 | Harley R.M. 57896 | Yes | No | Yes | rbcL, ITS2 |
| Ericales | Sapotaceae | *Pouteria ramiflora* (Mart.) Radlk. | CANGA136-17 | Vasconcelos L.V. 1044 | Yes | No | Yes | ITS2 |
| Ericales | Sapotaceae | *Pouteria ramiflora* (Mart.) Radlk. | CANGA137-17 | Vasconcelos L.V. 1045 | Yes | No | Yes | rbcL, ITS2 |
| Fabales | Fabaceae | *Abarema cochleata* (Willd.) Barneby & J.W.Grimes | CANGI222-17 | Harley R.M. 57917 | No | No | Yes | rbcL |
| Fabales | Fabaceae | *Abrus melanospermus* subsp. *tenuiflorus* (Spruce ex Benth.) D. Harder | CANGI304-17 | Harley R.M. 57445 | Yes | Yes | No | rbcL, ITS2, trnH-psbA |
| Fabales | Fabaceae | *Abrus melanospermus* subsp. *tenuiflorus* (Spruce ex Benth.) D. Harder | CANGI247-17 | Vasconcelos L.V. 1113 | Yes | No | No | rbcL, ITS2 |
| Fabales | Fabaceae | *Aeschynomene rudis* Benth. | ITVRT004-17 | Harley R.M. 57407 | No | Yes | No | rbcL, ITS2, rpoB, rpoC1, atpF-atpH, trnH-psbA |
| Fabales | Fabaceae | *Albizia niopoides* (Spruce ex Benth.) Burkart | CANGA479-20 | Zappi D.C. 3520 | No | No | No | rbcL |
| Fabales | Fabaceae | *Alysicarpus vaginalis* (L.) DC. | CANGI250-17 | Nogueira M.G.C. 664 | No | No | No | rbcL, ITS2 |
| Fabales | Fabaceae | *Ancistrotropis peduncularis* (Kunth) A. Delgado | CANGI244-17 | Viana P.L. 6115 | Yes | Yes | No | rbcL, ITS2 |
| Fabales | Fabaceae | *Bauhinia longicuspis* Benth. | CANGI225-17 | Pastore M. 564 | Yes | No | No | rbcL, ITS2 |
| Fabales | Fabaceae | *Bauhinia longipedicellata* Ducke | CANGI224-17 | Pastore M. 497 | Yes | No | Yes | rbcL, ITS2 |
| Fabales | Fabaceae | *Bauhinia pulchella* Benth. | CANGA360-17 | Vasconcelos L.V. 774 | Yes | Yes | No | rbcL |
| Fabales | Fabaceae | *Bauhinia pulchella* Benth. | CANGA361-17 | Viana P.L. 6187 | Yes | Yes | No | rbcL |
| Fabales | Fabaceae | *Bauhinia pulchella* Benth. | CANGA455-20 | Harley R.M. 57852 | Yes | No | No | rbcL, ITS2 |
| Fabales | Fabaceae | *Bauhinia pulchella* Benth. | CANGA362-17 | Vasconcelos L.V. 1131 | Yes | No | No | rbcL |
| Fabales | Fabaceae | *Bauhinia pulchella* Benth. | CANGA363-17 | Harley R.M. 58129 | Yes | No | No | rbcL |
| Fabales | Fabaceae | *Bauhinia pulchella* Benth. | CGII180-20 | Zappi D.C. 4451 | Yes | No | No | rbcL, ITS2 |
| Fabales | Fabaceae | *Cajanus cajan* (L.) Huth | CANGI252-17 | Pastore M. 573 | No | No | No | rbcL, ITS2 |
| Fabales | Fabaceae | *Calopogonium mucunoides* Desv. | CANGI246-17 | Vasconcelos L.V. 1081 | Yes | No | No | rbcL, ITS2 |
| Fabales | Fabaceae | *Camptosema ellipticum* (Desv.) Burkart | ITVRT010-17 | Viana P.L. 6201 | Yes | Yes | Yes | rbcL, ITS2 |
| Fabales | Fabaceae | *Camptosema ellipticum* (Desv.) Burkart | CANGA456-20 | Harley R.M. 57853 | Yes | No | Yes | rbcL, ITS2 |
| Fabales | Fabaceae | *Camptosema ellipticum* (Desv.) Burkart | CANGI249-17 | Vasconcelos L.V. 1135 | Yes | No | Yes | rbcL, ITS2 |
| Fabales | Fabaceae | *Centrosema carajasense* Cavalcante | CANGA063-17 | Vasconcelos L.V. 792 | Yes | Yes | Yes | rbcL, ITS2, trnH-psbA |
| Fabales | Fabaceae | *Centrosema carajasense* Cavalcante | CANGI226-17 | Viana P.L. 6204 | Yes | Yes | Yes | rbcL, ITS2 |
| Fabales | Fabaceae | *Centrosema carajasense* Cavalcante | CANGA062-17 | Vasconcelos L.V. 1146 | Yes | No | Yes | rbcL, ITS2 |
| Fabales | Fabaceae | *Chamaecrista desvauxii* (Collad.) Killip | CANGI227-17 | Harley R.M. 57906 | Yes | Yes | No | rbcL, ITS2 |
| Fabales | Fabaceae | *Chamaecrista nictitans* (L.) Moench | CANGI228-17 | Harley R.M. 58055 | Yes | No | No | rbcL, ITS2 |
| Fabales | Fabaceae | *Chamaecrista viscosa* (Kunth) H.S.Irwin & Barneby | CANGA478-20 | Giulietti A.M. 2616 | No | No | Yes | rbcL, ITS2 |
| Fabales | Fabaceae | *Clitoria arborea* Benth. | CANGI251-17 | Pastore M. 560 | No | No | Yes | rbcL, ITS2 |
| Fabales | Fabaceae | *Clitoria falcata* Lam. | CANGA178-17 | Meirelles J. 961 | Yes | Yes | Yes | rbcL, ITS2 |
| Fabales | Fabaceae | *Clitoria falcata* Lam. | CANGI248-17 | Vasconcelos L.V. 1128 | Yes | No | Yes | rbcL |
| Fabales | Fabaceae | *Clitoria falcata* Lam. | CANGI255-17 | Harley R.M. 58140 | Yes | No | Yes | rbcL, ITS2 |
| Fabales | Fabaceae | *Clitoria falcata* Lam. | CANGI257-17 | Harley R.M. 58145 | Yes | No | Yes | rbcL, ITS2 |
| Fabales | Fabaceae | *Clitoria leptostachya* var. *fruticosa* Fantz | CANGA255-17 | Harley R.M. 57257 | No | Yes | Yes | rbcL, matK, rpoB, atpF-atpH |
| Fabales | Fabaceae | *Copaifera martii* Hayne | CANGI229-17 | Harley R.M. 57997 | Yes | Yes | Yes | rbcL |
| Fabales | Fabaceae | *Crotalaria maypurensis* Kunth | CANGA282-17 | Viana P.L. 5570 | Yes | Yes | No | rbcL, ITS2, matK, rpoB, atpF-atpH, psbK-psbI |
| Fabales | Fabaceae | *Crotalaria maypurensis* Kunth | CANGI230-17 | Harley R.M. 58163 | Yes | No | No | rbcL, ITS2 |
| Fabales | Fabaceae | *Crotalaria maypurensis* Kunth | CANGA461-20 | Harley R.M. 57863 | Yes | No | No | ITS2 |
| Fabales | Fabaceae | *Crotalaria maypurensis* Kunth | CANGI628-17 | Vasconcelos L.V. 1141 | Yes | No | No | ITS2 |
| Fabales | Fabaceae | *Crotalaria maypurensis* Kunth | CANGI231-17 | Harley R.M. 58119 | Yes | No | No | rbcL, ITS2 |
| Fabales | Fabaceae | *Crotalaria maypurensis* Kunth | CGII176-20 | Zappi D.C. 4457 | Yes | No | No | ITS2 |
| Fabales | Fabaceae | *Deguelia amazonica* Killip | CANGI245-17 | Harley R.M. 57977 | Yes | Yes | Yes | rbcL, ITS2 |
| Fabales | Fabaceae | *Dioclea apurensis* Kunth | CANGA021-17 | Vasconcelos L.V. 779 | Yes | Yes | Yes | ITS2, trnH-psbA |
| Fabales | Fabaceae | *Dioclea apurensis* Kunth | CANGA460-20 | Harley R.M. 57862 | Yes | No | Yes | ITS2 |
| Fabales | Fabaceae | *Dioclea apurensis* Kunth | CANGA466-20 | Harley R.M. 57872 | Yes | No | Yes | ITS2 |
| Fabales | Fabaceae | *Dioclea apurensis* Kunth | CANGI253-17 | Pastore M. 592 | Yes | No | Yes | ITS2 |
| Fabales | Fabaceae | *Dioclea bicolor* Benth. | ITVRT011-17 | Vasconcelos L.V. 6206 | Yes | Yes | Yes | rbcL, ITS2 |
| Fabales | Fabaceae | *Dioclea bicolor* Benth. | CANGA452-20 | Harley R.M. 57859 | Yes | No | Yes | rbcL, ITS2 |
| Fabales | Fabaceae | *Dioclea virgata* (Rich.) Amshoff | CANGI627-17 | Vasconcelos L.V. 1138 | No | No | No | ITS2 |
| Fabales | Fabaceae | *Galactia jussiaeana* Kunth | CANGA246-17 | Harley R.M. 57431 | Yes | Yes | Yes | rbcL, ITS2, atpF-atpH |
| Fabales | Fabaceae | *Galactia jussiaeana* Kunth | CANGI256-17 | Harley R.M. 58141 | Yes | No | Yes | rbcL, ITS2 |
| Fabales | Fabaceae | *Harpalyce brasiliana* Benth. | CGII659-20 | Zappi D.C. 4527 | No | No | No | rbcL, ITS2 |
| Fabales | Fabaceae | *Hymenaea parvifolia* Huber | CANGA338-17 | Giulietti A.M. 2644 | No | No | No | rbcL, ITS2 |
| Fabales | Fabaceae | *Inga alba* (Sw.) Willd. | CANGI232-17 | Nunes C.S. 98 | Yes | Yes | No | rbcL, ITS2, rpoC1 |
| Fabales | Fabaceae | *Inga rubiginosa* (Rich.) DC. | CANGI233-17 | Harley R.M. 57987 | No | Yes | No | rbcL, ITS2 |
| Fabales | Fabaceae | *Machaerium latifolium* Rusby | CANGI234-17 | Afonso E.A.L 129 | Yes | Yes | Yes | ITS2, rpoC1 |
| Fabales | Fabaceae | *Macroptilium gracile* (Poepp. ex Benth.) Urb. | CGII624-20 | Zappi D.C. 4328 | No | No | No | rbcL, ITS2 |
| Fabales | Fabaceae | *Mimosa acutistipula* var. *ferrea* Barneby | CANGA007-17 | Harley R.M. 57126 | Yes | Yes | No | rbcL, ITS2, matK, trnH-psbA |
| Fabales | Fabaceae | *Mimosa acutistipula* var. *ferrea* Barneby | CANGA005-17 | Harley R.M. 57414 | Yes | Yes | No | ITS2 |
| Fabales | Fabaceae | *Mimosa acutistipula* var. *ferrea* Barneby | CANGA453-20 | Harley R.M. 57850 | Yes | No | No | ITS2 |
| Fabales | Fabaceae | *Mimosa dasilvae* A.S.Silva & R.Secco | CANGA153-17 | Viana P.L. 6106 | No | Yes | Yes | rbcL, ITS2 |
| Fabales | Fabaceae | *Mimosa piresii* Barneby | CANGA354-17 | Vasconcelos L.V. 857 | Yes | Yes | Yes | rbcL, ITS2 |
| Fabales | Fabaceae | *Mimosa skinneri* var. *carajarum* Barneby | CANGA398-17 | Harley R.M. 57410 | Yes | Yes | No | rbcL, ITS2, matK, rpoB, rpoC1 |
| Fabales | Fabaceae | *Mimosa skinneri* var. *carajarum* Barneby | CANGA399-17 | Harley R.M. 57464 | Yes | Yes | No | rbcL, ITS2, trnH-psbA |
| Fabales | Fabaceae | *Mimosa skinneri* var. *carajarum* Barneby | CANGA400-17 | Viana P.L. 6120 | Yes | Yes | No | rbcL, ITS2 |
| Fabales | Fabaceae | *Mimosa skinneri* var. *carajarum* Barneby | CANGI236-17 | Viana P.L. 6192 | Yes | Yes | No | rbcL, ITS2 |
| Fabales | Fabaceae | *Mimosa skinneri* var. *carajarum* Barneby | CANGI237-17 | Harley R.M. 58170 | Yes | No | No | rbcL, ITS2 |
| Fabales | Fabaceae | *Mimosa skinneri* var. *carajarum* Barneby | CANGA401-17 | Harley R.M. 57479 | Yes | Yes | No | rbcL, ITS2 |
| Fabales | Fabaceae | *Mimosa skinneri* var. *carajarum* Barneby | CANGI238-17 | Harley R.M. 57504 | Yes | Yes | No | rbcL, ITS2 |
| Fabales | Fabaceae | *Mimosa skinneri* var. *carajarum* Barneby | CANGA395-17 | Vasconcelos L.V. 1123 | Yes | No | No | rbcL, ITS2 |
| Fabales | Fabaceae | *Mimosa skinneri* var. *carajarum* Barneby | CANGA396-17 | Pastore M. 589 | Yes | No | No | rbcL |
| Fabales | Fabaceae | *Mimosa skinneri* var. *carajarum* Barneby | CANGA397-17 | Harley R.M. 58126 | Yes | No | No | rbcL |
| Fabales | Fabaceae | *Mimosa somnians* var. *viscida* (Willd.) Barneby | CANGA403-17 | Harley R.M. 57449 | Yes | Yes | Yes | rbcL, trnH-psbA |
| Fabales | Fabaceae | *Mimosa somnians* var. *viscida* (Willd.) Barneby | CANGA402-17 | Viana P.L. 6237 | Yes | Yes | Yes | rbcL, ITS2 |
| Fabales | Fabaceae | *Mimosa somnians* var. *viscida* (Willd.) Barneby | CANGA404-17 | Harley R.M. 57480 | Yes | Yes | Yes | rbcL, ITS2 |
| Fabales | Fabaceae | *Mimosa somnians* var. *viscida* (Willd.) Barneby | CANGI239-17 | Vasconcelos L.V. 1069 | Yes | No | Yes | rbcL, ITS2 |
| Fabales | Fabaceae | *Mimosa xanthocentra* var. *mansii* (Mart.) Barneby | CANGA436-17 | Viana P.L. 6158 | Yes | Yes | No | rbcL, ITS2 |
| Fabales | Fabaceae | *Mimosa xanthocentra* var. *mansii* (Mart.) Barneby | CANGI240-17 | Pastore M. 499 | Yes | No | No | rbcL, ITS2 |
| Fabales | Fabaceae | *Mimosa xanthocentra* var. *mansii* (Mart.) Barneby | CANGI241-17 | Pastore M. 576 | Yes | No | No | rbcL, ITS2 |
| Fabales | Fabaceae | *Mimosa xanthocentra* var. *mansii* (Mart.) Barneby | CANGI242-17 | Harley R.M. 58124 | Yes | No | No | rbcL, ITS2 |
| Fabales | Fabaceae | *Parkia platycephala* Benth. | CANGA355-17 | Mota N.F.O. 3418 | Yes | Yes | Yes | rbcL, ITS2, matK, rpoB, rpoC1, atpF-atpH, psbK-psbI |
| Fabales | Fabaceae | *Parkia platycephala* Benth. | ITVRT009-17 | Vasconcelos L.V. 866 | Yes | Yes | Yes | rbcL, ITS2 |
| Fabales | Fabaceae | *Parkia platycephala* Benth. | ITVRT020-17 | Vasconcelos L.V. 939 | Yes | No | Yes | rbcL, ITS2 |
| Fabales | Fabaceae | *Periandra mediterranea* (Vell.) Taub. | CANGA283-17 | Vasconcelos L.V. 767 | Yes | Yes | Yes | rbcL, ITS2 |
| Fabales | Fabaceae | *Periandra mediterranea* (Vell.) Taub. | CANGA451-20 | Harley R.M. 57871 | Yes | Yes | Yes | rbcL, ITS2 |
| Fabales | Fabaceae | *Periandra mediterranea* (Vell.) Taub. | CANGA284-17 | Vasconcelos L.V. 1149 | Yes | No | Yes | rbcL, ITS2 |
| Fabales | Fabaceae | *Periandra mediterranea* (Vell.) Taub. | ITVGA008-17 | Gastauer M. s.n. | Yes | No | Yes | rbcL, ITS2 |
| Fabales | Fabaceae | *Senegalia polyphylla* (DC.) Britton & Rose | CANGI223-17 | Pastore M. 552 | No | No | Yes | rbcL, ITS2 |
| Fabales | Fabaceae | *Senna latifolia* (G.Mey.) H.S.Irwin & Barneby | CANGI243-17 | Nunes C.S. 99 | No | Yes | Yes | matK, rpoB, rpoC1 |
| Fabales | Fabaceae | *Stylosanthes capitata* Vogel | CANGA056-17 | Viana P.L. 6193 | Yes | Yes | No | rbcL, ITS2 |
| Fabales | Fabaceae | *Swartzia arumateuana* (R. S. Cowan) Torke & Mansano | CANGI071-17 | Harley R.M. 58058 | No | No | Yes | rbcL, ITS2 |
| Fabales | Polygalaceae | *Bredemeyera floribunda* Willd. | CANGA201-17 | Vasconcelos L.V. 838 | Yes | Yes | No | rbcL, ITS2 |
| Fabales | Polygalaceae | *Bredemeyera floribunda* Willd. | CANGI482-17 | Harley R.M. 57903 | Yes | Yes | No | rbcL |
| Fabales | Polygalaceae | *Bredemeyera floribunda* Willd. | CANGA459-20 | Harley R.M. 57861 | Yes | No | No | rbcL, ITS2 |
| Fabales | Polygalaceae | *Caamembeca spectabilis* (DC.) J.F.B.Pastore | CANGA405-17 | Trindade J.R. 338 | Yes | Yes | Yes | rbcL, ITS2, psbK-psbI |
| Fabales | Polygalaceae | *Caamembeca spectabilis* (DC.) J.F.B.Pastore | CANGI483-17 | Praia T.S. 17 | Yes | Yes | Yes | rbcL, rpoC1, atpF-atpH, psbK-psbI |
| Fabales | Polygalaceae | *Caamembeca spectabilis* (DC.) J.F.B.Pastore | CANGI488-17 | Pastore M. 520 | Yes | No | Yes | rbcL, ITS2 |
| Fabales | Polygalaceae | *Polygala adenophora* DC. | CANGA008-17 | Vasconcelos L.V. 815 | Yes | Yes | No | rbcL, ITS2 |
| Fabales | Polygalaceae | *Polygala adenophora* DC. | CANGA009-17 | Viana P.L. 6143 | Yes | Yes | No | rbcL, ITS2 |
| Fabales | Polygalaceae | *Polygala adenophora* DC. | CANGI485-17 | Harley R.M. 58116 | Yes | No | No | rbcL, ITS2 |
| Fabales | Polygalaceae | *Polygala adenophora* DC. | CGII638-20 | Zappi D.C. 4380 | Yes | No | No | rbcL, ITS2 |
| Fabales | Polygalaceae | *Polygala celosioides* Mart. ex A.W.Benn. | CANGI489-17 | Harley R.M. 58143 | No | No | No | rbcL, ITS2 |
| Fabales | Polygalaceae | *Polygala paniculata* L. | CANGI484-17 | Prado M.L 378 | No | Yes | No | rbcL, ITS2, matK, rpoC1, atpF-atpH |
| Fabales | Polygalaceae | *Polygala timoutou* Aubl. | CGII637-20 | Zappi D.C. 4379 | No | No | Yes | rbcL, ITS2 |
| Fabales | Polygalaceae | *Securidaca diversifolia* (L.) S.F.Blake | CANGI486-17 | Harley R.M. 57904 | Yes | Yes | No | rbcL, ITS2 |
| Fabales | Polygalaceae | *Securidaca rivinifolia* A.St.-Hil. & Moq. | CANGA377-17 | Gil A. 525 | Yes | Yes | Yes | rbcL, ITS2, matK, rpoC1 |
| Fabales | Polygalaceae | *Securidaca rivinifolia* A.St.-Hil. & Moq. | CANGA378-17 | Gil A. 509 | Yes | Yes | Yes | rbcL, ITS2, matK, rpoC1 |
| Fabales | Polygalaceae | *Securidaca rivinifolia* A.St.-Hil. & Moq. | CANGI487-17 | Harley R.M. 57975 | Yes | Yes | Yes | rbcL |
| Fabales | Polygalaceae | *Securidaca rivinifolia* A.St.-Hil. & Moq. | CGII666-20 | Zappi D.C. 4504 | Yes | No | Yes | rbcL |
| Gentianales | Apocynaceae | *Asclepias curassavica* L. | CANGI019-17 | Nunes C.S. 86 | Yes | Yes | No | rbcL, atpF-atpH |
| Gentianales | Apocynaceae | *Blepharodon pictum* (Vahl) W.D.Stevens | CANGI018-17 | Vasconcelos L.V. 1024 | Yes | No | No | rbcL, ITS2 |
| Gentianales | Apocynaceae | *Forsteronia affinis* Müll.Arg. | ITVRT007-17 | Simoes A.O. 1809 | Yes | Yes | Yes | ITS2, matK, rpoB, rpoC1, atpF-atpH |
| Gentianales | Apocynaceae | *Hemipogon sprucei* E.Fourn. | CANGI029-17 | Trindade J.R. 371 | Yes | Yes | No | ITS2, rpoB, atpF-atpH, psbK-psbI |
| Gentianales | Apocynaceae | *Hemipogon sprucei* E.Fourn. | CANGI020-17 | Harley R.M. 57498 | Yes | Yes | No | rbcL |
| Gentianales | Apocynaceae | *Mandevilla scabra* (Hoffmanns. ex Roem. & Schult.) K.Schum. | CANGI022-17 | Harley R.M. 57417 | Yes | Yes | No | rbcL, ITS2, matK, rpoB, rpoC1 |
| Gentianales | Apocynaceae | *Mandevilla scabra* (Hoffmanns. ex Roem. & Schult.) K.Schum. | CANGA381-17 | Vasconcelos L.V. 780 | Yes | Yes | No | rbcL, ITS2 |
| Gentianales | Apocynaceae | *Mandevilla scabra* (Hoffmanns. ex Roem. & Schult.) K.Schum. | CANGA382-17 | Viana P.L. 6196 | Yes | Yes | No | rbcL, ITS2 |
| Gentianales | Apocynaceae | *Mandevilla scabra* (Hoffmanns. ex Roem. & Schult.) K.Schum. | CANGA383-17 | Vasconcelos L.V. 1126 | Yes | No | No | rbcL, ITS2 |
| Gentianales | Apocynaceae | *Mandevilla scabra* (Hoffmanns. ex Roem. & Schult.) K.Schum. | CANGI034-17 | Harley R.M. 58135 | Yes | No | No | rbcL, ITS2 |
| Gentianales | Apocynaceae | *Mandevilla tenuifolia* (J.C.Mikan) Woodson | CANGA420-17 | Harley R.M. 58096 | Yes | No | No | rbcL, ITS2 |
| Gentianales | Apocynaceae | *Marsdenia bergii* Morillo | CANGA032-17 | Vasconcelos L.V. 1109 | Yes | No | Yes | rbcL, ITS2 |
| Gentianales | Apocynaceae | *Marsdenia bergii* Morillo | CANGA033-17 | Vasconcelos L.V. 1119 | Yes | No | Yes | rbcL, ITS2 |
| Gentianales | Apocynaceae | *Minaria cordata* (Turcz.) T.U.P.Konno & Rapini | CANGI030-17 | Viana P.L. 6226 | No | Yes | No | rbcL |
| Gentianales | Apocynaceae | *Prestonia* cf. *annularis* (L.f.) G.Don | CANGI025-17 | Nogueira M.G.C. 656 | No | No | Yes | rbcL, ITS2 |
| Gentianales | Apocynaceae | *Secondatia densiflora* A.DC. | CANGI027-17 | Gil A. 511 | Yes | Yes | No | rbcL, ITS2, matK, rpoC1 |
| Gentianales | Apocynaceae | *Secondatia densiflora* A.DC. | ITVRT008-17 | Praia T.S. 23 | Yes | Yes | No | rbcL, ITS2, rpoC1, atpF-atpH |
| Gentianales | Apocynaceae | *Secondatia densiflora* A.DC. | ITVRT024-17 | Vasconcelos L.V. 1029 | Yes | No | No | rbcL, ITS2 |
| Gentianales | Apocynaceae | *Tabernaemontana* cf. *heterophylla* Vahl | CANGI033-17 | Harley R.M. 57933 | Yes | No | No | rbcL |
| Gentianales | Apocynaceae | *Tabernaemontana flavicans* Willd. ex Roem. & Schult. | ITVRT005-17 | Harley R.M. 57427 | Yes | Yes | No | rbcL, ITS2, matK, rpoB, rpoC1, atpF-atpH, trnH-psbA |
| Gentianales | Apocynaceae | *Tabernaemontana flavicans* Willd. ex Roem. & Schult. | CANGI028-17 | Harley R.M. 57457 | Yes | Yes | No | rbcL, ITS2, trnH-psbA |
| Gentianales | Apocynaceae | *Tabernaemontana macrocalyx* Müll. Arg. | CANGI026-17 | Simoes A.O. 1807 | Yes | Yes | No | ITS2, matK, rpoC1, atpF-atpH |
| Gentianales | Apocynaceae | *Tabernaemontana macrocalyx* Müll. Arg. | CANGI031-17 | Harley R.M. 57985 | Yes | Yes | No | rbcL, ITS2 |
| Gentianales | Gentianaceae | *Chelonanthus purpurascens* (Aubl.) Struwe, S. Nilsson & V.A. Albert | CANGI258-17 | Harley R.M. 57437 | Yes | Yes | No | rbcL, ITS2 |
| Gentianales | Gentianaceae | *Chelonanthus purpurascens* (Aubl.) Struwe, S. Nilsson & V.A. Albert | CANGI516-17 | Vasconcelos L.V. 786 | Yes | Yes | No | rbcL, ITS2, trnH-psbA |
| Gentianales | Gentianaceae | *Coutoubea ramosa* Aubl. | CANGI262-17 | Nogueira M.G.C. 666 | No | No | No | rbcL, ITS2 |
| Gentianales | Gentianaceae | *Schultesia benthamiana* Klotzsch ex Griseb. | CANGA366-17 | Vasconcelos L.V. 788 | Yes | Yes | Yes | rbcL, ITS2, trnH-psbA |
| Gentianales | Gentianaceae | *Schultesia benthamiana* Klotzsch ex Griseb. | CANGI260-17 | Viana P.L. 6144 | Yes | Yes | Yes | rbcL, ITS2 |
| Gentianales | Gentianaceae | *Schultesia benthamiana* Klotzsch ex Griseb. | CANGI261-17 | Pastore M. 582 | Yes | No | Yes | rbcL |
| Gentianales | Gentianaceae | *Schultesia benthamiana* Klotzsch ex Griseb. | CANGI263-17 | Harley R.M. 58148 | Yes | No | Yes | rbcL |
| Gentianales | Loganiaceae | *Antonia ovata* Pohl | CANGA469-20 | Giulietti A.M. 2671 | No | No | No | rbcL, ITS2 |
| Gentianales | Loganiaceae | *Antonia ovata* Pohl | CGII663-20 | Zappi D.C. 4521 | No | No | No | rbcL, ITS2 |
| Gentianales | Loganiaceae | *Spigelia anthelmia* L. | CANGA019-17 | Nogueira M.G.C. 694 | Yes | No | No | rbcL, ITS2 |
| Gentianales | Loganiaceae | *Strychnos cogens* Benth. | CANGA468-20 | Harley R.M. 57882 | Yes | No | No | rbcL, ITS2 |
| Gentianales | Rubiaceae | *Borreria alata* (Aubl.) DC. | CANGI544-17 | Vasconcelos L.V. 763 | Yes | Yes | Yes | rbcL, ITS2 |
| Gentianales | Rubiaceae | *Borreria carajasensis* E.L. Cabral & L.M. Miguel | CANGI515-17 | Vasconcelos L.V. 772 | Yes | Yes | Yes | rbcL, ITS2 |
| Gentianales | Rubiaceae | *Borreria carajasensis* E.L. Cabral & L.M. Miguel | CANGI532-17 | Vasconcelos L.V. 861 | Yes | Yes | Yes | ITS2 |
| Gentianales | Rubiaceae | *Borreria carajasensis* E.L. Cabral & L.M. Miguel | CANGA103-17 | Viana P.L. 6112 | Yes | Yes | Yes | rbcL, ITS2 |
| Gentianales | Rubiaceae | *Borreria carajasensis* E.L. Cabral & L.M. Miguel | CANGI517-17 | Viana P.L. 6184 | Yes | Yes | Yes | rbcL, ITS2 |
| Gentianales | Rubiaceae | *Borreria carajasensis* E.L. Cabral & L.M. Miguel | CANGI545-17 | Pastore M. 658 | Yes | No | Yes | rbcL, ITS2 |
| Gentianales | Rubiaceae | *Borreria carajasensis* E.L. Cabral & L.M. Miguel | CANGI546-17 | Pastore M. 634 | Yes | No | Yes | rbcL, ITS2 |
| Gentianales | Rubiaceae | *Borreria carajasensis* E.L. Cabral & L.M. Miguel | CANGI519-17 | Vasconcelos L.V. 1080 | Yes | No | Yes | rbcL, ITS2 |
| Gentianales | Rubiaceae | *Borreria elaiosulcata* E.L. Cabral & L.M. Miguel | CANGA168-17 | Mota N.F.O. 3417 | Yes | Yes | Yes | rbcL, ITS2, matK, rpoB, atpF-atpH |
| Gentianales | Rubiaceae | *Borreria elaiosulcata* E.L. Cabral & L.M. Miguel | CANGA172-17 | Pastore M. 350 | Yes | Yes | Yes | rbcL, ITS2 |
| Gentianales | Rubiaceae | *Borreria elaiosulcata* E.L. Cabral & L.M. Miguel | CANGA173-17 | Viana P.L. 6222 | Yes | Yes | Yes | rbcL, ITS2 |
| Gentianales | Rubiaceae | *Borreria elaiosulcata* E.L. Cabral & L.M. Miguel | CANGA128-17 | Vasconcelos L.V. 1026 | Yes | No | Yes | rbcL, ITS2 |
| Gentianales | Rubiaceae | *Borreria heteranthera* E.L. Cabral & Sobrado | CANGI518-17 | Viana P.L. 6221 | Yes | Yes | Yes | rbcL, ITS2 |
| Gentianales | Rubiaceae | *Borreria hispida* Spruce ex K.Schum. | CANGA389-17 | Vasconcelos L.V. 794 | Yes | Yes | Yes | rbcL, ITS2, trnH-psbA |
| Gentianales | Rubiaceae | *Borreria hispida* Spruce ex K.Schum. | CANGA390-17 | Vasconcelos L.V. 821 | Yes | Yes | Yes | rbcL, ITS2 |
| Gentianales | Rubiaceae | *Borreria hispida* Spruce ex K.Schum. | CANGI525-17 | Pastore M. 578 | Yes | No | Yes | rbcL, ITS2 |
| Gentianales | Rubiaceae | *Borreria ocymifolia* (Roem. & Schult.) Bacigalupo & E.L.Cabral | CANGI514-17 | Rocha K. 57 | Yes | Yes | Yes | ITS2 |
| Gentianales | Rubiaceae | *Borreria paraensis* E.L.Cabral & Bacigalupo | CANGA336-17 | Vasconcelos L.V. 1053 | Yes | No | Yes | rbcL, ITS2 |
| Gentianales | Rubiaceae | *Borreria paraensis* E.L.Cabral & Bacigalupo | CANGI520-17 | Vasconcelos L.V. 1129 | Yes | No | Yes | rbcL, ITS2 |
| Gentianales | Rubiaceae | *Borreria paraensis* E.L.Cabral & Bacigalupo | CANGI526-17 | Harley R.M. 58086 | Yes | No | Yes | rbcL, ITS2 |
| Gentianales | Rubiaceae | *Borreria paraensis* E.L.Cabral & Bacigalupo | CANGI527-17 | Harley R.M. 58103 | Yes | No | Yes | rbcL, ITS2 |
| Gentianales | Rubiaceae | *Borreria semiamplexicaulis* E.L.Cabral | CANGA438-17 | Viana P.L. 5566 | Yes | Yes | Yes | rpoB |
| Gentianales | Rubiaceae | *Borreria semiamplexicaulis* E.L.Cabral | CANGA388-17 | Pastore M. 313 | Yes | Yes | Yes | rbcL, ITS2 |
| Gentianales | Rubiaceae | *Borreria semiamplexicaulis* E.L.Cabral | CANGI533-17 | Viana P.L. 6180 | Yes | Yes | Yes | rbcL, ITS2 |
| Gentianales | Rubiaceae | *Borreria semiamplexicaulis* E.L.Cabral | CANGI528-17 | Harley R.M. 58104 | Yes | No | Yes | rbcL, ITS2 |
| Gentianales | Rubiaceae | *Borreria tenella* (Kunth) Cham. & Schltdl. | CANGI523-17 | Pastore M. 506 | No | No | Yes | rbcL, ITS2 |
| Gentianales | Rubiaceae | *Borreria verticillata* (L.) G.Mey. | CANGA431-17 | Trindade J.R. 342 | Yes | Yes | Yes | ITS2, matK, rpoB, rpoC1, psbK-psbI |
| Gentianales | Rubiaceae | *Borreria verticillata* (L.) G.Mey. | CANGA432-17 | Lopes C.S.A. 5 | Yes | Yes | Yes | rbcL |
| Gentianales | Rubiaceae | *Borreria verticillata* (L.) G.Mey. | CANGI521-17 | Vasconcelos L.V. 1140 | Yes | No | Yes | rbcL, ITS2 |
| Gentianales | Rubiaceae | *Carajasia cangae* R.M. Salas, E.L. Cabral & Dessein | CANGA053-17 | Cardoso A. 2015 | Yes | Yes | Yes | rpoB |
| Gentianales | Rubiaceae | *Chomelia ribesioides* Benth. ex A.Gray | CANGA376-17 | Vasconcelos L.V. 1051 | Yes | No | Yes | rbcL, ITS2 |
| Gentianales | Rubiaceae | *Chomelia ribesioides* Benth. ex A.Gray | CANGI538-17 | Harley R.M. 58091 | Yes | No | Yes | rbcL, ITS2 |
| Gentianales | Rubiaceae | *Cordiera myrciifolia* (K.Schum.) C.H.Perss. & Delprete | CANGA306-17 | Harley R.M. 57333 | Yes | Yes | Yes | rbcL, ITS2, matK, rpoC1 |
| Gentianales | Rubiaceae | *Cordiera myrciifolia* (K.Schum.) C.H.Perss. & Delprete | CANGI530-17 | Gil A. 523 | Yes | Yes | Yes | rbcL, ITS2, matK, rpoC1 |
| Gentianales | Rubiaceae | *Cordiera myrciifolia* (K.Schum.) C.H.Perss. & Delprete | CANGI541-17 | Trindade J.R. 370 | Yes | Yes | Yes | ITS2, matK, rpoB, rpoC1, atpF-atpH, psbK-psbI |
| Gentianales | Rubiaceae | *Cordiera myrciifolia* (K.Schum.) C.H.Perss. & Delprete | CANGA166-17 | Vasconcelos L.V. 909 | Yes | No | Yes | rbcL |
| Gentianales | Rubiaceae | *Cordiera sessilis* (Vell.) Kuntze | CGII653-20 | Zappi D.C. 4553 | No | No | Yes | rbcL |
| Gentianales | Rubiaceae | *Faramea multiflora* A.Rich. ex DC. | NA | Pastore M. 307 | Yes | Yes | NA |  |
| Gentianales | Rubiaceae | *Limnosipanea spruceana* Hook.f. | CANGI443-17 | Mota N.F.O. 3406 | Yes | Yes | No | rbcL, ITS2, rpoB, rpoC1, atpF-atpH |
| Gentianales | Rubiaceae | *Mitracarpus carajasensis* E.L.Cabral, Sobrado & E.B.Souza | CANGA080-17 | Cardoso A. 1958 | Yes | Yes | Yes | rbcL, ITS2, matK, psbK-psbI |
| Gentianales | Rubiaceae | *Mitracarpus carajasensis* E.L.Cabral, Sobrado & E.B.Souza | CANGI531-17 | Praia T.S. 12 | Yes | Yes | Yes | rbcL, rpoB |
| Gentianales | Rubiaceae | *Palicourea hoffmannseggiana* (Willd. ex Schult.) Borhidi | CANGA230-17 | Trindade J.R. 354 | Yes | Yes | Yes | ITS2, rpoB, rpoC1, atpF-atpH, psbK-psbI |
| Gentianales | Rubiaceae | *Palicourea hoffmannseggiana* (Willd. ex Schult.) Borhidi | CANGA231-17 | Vasconcelos L.V. 1050 | Yes | No | Yes | rbcL, ITS2 |
| Gentianales | Rubiaceae | *Palicourea hoffmannseggiana* (Willd. ex Schult.) Borhidi | CANGA233-17 | Vasconcelos L.V. 1115 | Yes | No | Yes | rbcL, ITS2 |
| Gentianales | Rubiaceae | *Palicourea hoffmannseggiana* (Willd. ex Schult.) Borhidi | CANGA232-17 | Vasconcelos L.V. 1134 | Yes | No | Yes | ITS2 |
| Gentianales | Rubiaceae | *Palicourea marcgravii* A.St.-Hil. | CANGA279-17 | Rocha K. 65 | Yes | Yes | Yes | rbcL, rpoB, rpoC1, atpF-atpH |
| Gentianales | Rubiaceae | *Palicourea marcgravii* A.St.-Hil. | CANGI534-17 | Praia T.S. 14 | Yes | Yes | Yes | rbcL, atpF-atpH |
| Gentianales | Rubiaceae | *Palicourea marcgravii* A.St.-Hil. | CANGA278-17 | Viana P.L. 6198 | Yes | Yes | Yes | rbcL, ITS2 |
| Gentianales | Rubiaceae | *Palicourea racemosa* (Aubl.) G.Nicholson | CANGA370-17 | Afonso E.A.L 137 | Yes | Yes | Yes | ITS2, rpoB, rpoC1, atpF-atpH, psbK-psbI |
| Gentianales | Rubiaceae | *Palicourea tomentosa* Müll.Arg. | CANGI537-17 | Harley R.M. 57936 | No | No | No | rbcL |
| Gentianales | Rubiaceae | *Perama carajensis* (Aubl.) Borhidi | CANGA105-17 | Cardoso A. 1944 | Yes | Yes | Yes | rbcL, ITS2, matK, rpoB, rpoC1, atpF-atpH, psbK-psbI |
| Gentianales | Rubiaceae | *Perama carajensis* (Aubl.) Borhidi | CANGA106-17 | Viana P.L. 6214 | Yes | Yes | Yes | rbcL, ITS2 |
| Gentianales | Rubiaceae | *Perama carajensis* (Aubl.) Borhidi | CANGA107-17 | Viana P.L. 6227 | Yes | Yes | Yes | rbcL, ITS2 |
| Gentianales | Rubiaceae | *Perama carajensis* (Aubl.) Borhidi | CANGI535-17 | Harley R.M. 58158 | Yes | No | Yes | rbcL, ITS2 |
| Gentianales | Rubiaceae | *Perama carajensis* (Aubl.) Borhidi | CANGI536-17 | Pastore M. 635 | Yes | No | Yes | rbcL, ITS2 |
| Gentianales | Rubiaceae | *Perama carajensis* (Aubl.) Borhidi | CANGA108-17 | Viana P.L. 6103 | Yes | Yes | Yes | rbcL, ITS2 |
| Gentianales | Rubiaceae | *Perama carajensis* (Aubl.) Borhidi | CANGA109-17 | Vasconcelos L.V. 768 | Yes | No | Yes | rbcL, ITS2 |
| Gentianales | Rubiaceae | *Perama carajensis* (Aubl.) Borhidi | CANGA110-17 | Vasconcelos L.V. 1122 | Yes | No | Yes | rbcL, ITS2 |
| Gentianales | Rubiaceae | *Perama carajensis* (Aubl.) Borhidi | CANGA111-17 | Pastore M. 591 | Yes | No | Yes | rbcL, ITS2 |
| Gentianales | Rubiaceae | *Perama carajensis* (Aubl.) Borhidi | CANGA104-17 | Harley R.M. 58150 | Yes | No | Yes | rbcL, ITS2 |
| Gentianales | Rubiaceae | *Perama hirsuta* J.H.Kirkbr. | CGII636-20 | Zappi D.C. 4371 | No | No | Yes | rbcL, ITS2 |
| Gentianales | Rubiaceae | *Psychotria iodotricha* Aubl. | CANGI543-17 | Nunes C.S. 105 | Yes | Yes | Yes | rbcL, ITS2 |
| Gentianales | Rubiaceae | *Psychotria variegata* Steyerm. | CANGI542-17 | Nunes C.S. 102 | Yes | Yes | Yes | rbcL, trnH-psbA |
| Gentianales | Rubiaceae | *Richardia brasiliensis* Gomes | CANGI524-17 | Nogueira M.G.C. 669 | No | No | No | rbcL, ITS2 |
| Gentianales | Rubiaceae | *Sabicea grisea* Cham. & Schltdl. | CANGI539-17 | Vasconcelos L.V. 1055 | Yes | No | Yes | ITS2 |
| Gentianales | Rubiaceae | *Tocoyena formosa* (Cham. & Schltdl.) K.Schum. | CANGI540-17 | Vasconcelos L.V. 1062 | Yes | No | Yes | rbcL, ITS2 |
| Gentianales | Rubiaceae | *Tocoyena formosa* (Cham. & Schltdl.) K.Schum. | CANGA204-17 | Vasconcelos L.V. 1098 | Yes | No | Yes | rbcL, ITS2 |
| Gnetales | Gnetaceae | *Gnetum nodiflorum* Brongn. | CANGA319-17 | Mota N.F.O. 3430 | Yes | Yes | No | rbcL, ITS2, rpoC1 |
| Gnetales | Gnetaceae | *Gnetum nodiflorum* Brongn. | CANGA320-17 | Mota N.F.O. 3386 | Yes | Yes | No | rbcL, ITS2, atpF-atpH |
| Gnetales | Gnetaceae | *Gnetum nodiflorum* Brongn. | CANGA321-17 | Harley R.M. 57883 | Yes | No | No | rbcL, ITS2 |
| Isoetales | Isoetaceae | *Isoetes cangae* J.B.S.Pereira, Salino & Stützel | ISO024-17 | Santos F. 319 | Yes | Yes | Yes | ITS2, atpF-atpH, psbK-psbI, trnH-psbA |
| Isoetales | Isoetaceae | *Isoetes cangae* J.B.S.Pereira, Salino & Stützel | ISO025-17 | Santos F. s.n. | Yes | Yes | Yes | ITS2, atpF-atpH, psbK-psbI, trnH-psbA |
| Isoetales | Isoetaceae | *Isoetes cangae* J.B.S.Pereira, Salino & Stützel | ISO026-17 | Santos F. s.n. | Yes | Yes | Yes | ITS2, atpF-atpH, psbK-psbI, trnH-psbA |
| Isoetales | Isoetaceae | *Isoetes cangae* J.B.S.Pereira, Salino & Stützel | ISO120-17 | Santos F. s.n. | Yes | No | Yes | ITS2, trnH-psbA |
| Isoetales | Isoetaceae | *Isoetes cangae* J.B.S.Pereira, Salino & Stützel | ISO121-17 | Santos F. s.n. | Yes | No | Yes | ITS2, trnH-psbA |
| Isoetales | Isoetaceae | *Isoetes cangae* J.B.S.Pereira, Salino & Stützel | ISO122-17 | Santos F. s.n. | Yes | No | Yes | ITS2, trnH-psbA |
| Isoetales | Isoetaceae | *Isoetes cangae* J.B.S.Pereira, Salino & Stützel | ISO123-17 | Santos F. s.n. | Yes | No | Yes | ITS2, trnH-psbA |
| Isoetales | Isoetaceae | *Isoetes cangae* J.B.S.Pereira, Salino & Stützel | ISO124-17 | Santos F. s.n. | Yes | No | Yes | ITS2, trnH-psbA |
| Isoetales | Isoetaceae | *Isoetes cangae* J.B.S.Pereira, Salino & Stützel | ISO125-17 | Santos F. s.n. | Yes | No | Yes | ITS2, trnH-psbA |
| Isoetales | Isoetaceae | *Isoetes cangae* J.B.S.Pereira, Salino & Stützel | ISO126-17 | Santos F. s.n. | Yes | No | Yes | ITS2, trnH-psbA |
| Isoetales | Isoetaceae | *Isoetes serracarajensis* J.B.S.Pereira, Salino & Stützel | ISO001-17 | Falcão B.F. 201 | Yes | Yes | Yes | ITS2, atpF-atpH, psbK-psbI, trnH-psbA |
| Isoetales | Isoetaceae | *Isoetes serracarajensis* J.B.S.Pereira, Salino & Stützel | ISO002-17 | Santos F. E1N301FEV | Yes | Yes | Yes | ITS2, atpF-atpH, psbK-psbI, trnH-psbA |
| Isoetales | Isoetaceae | *Isoetes serracarajensis* J.B.S.Pereira, Salino & Stützel | ISO003-17 | Santos F. s.n. | Yes | Yes | Yes | ITS2, atpF-atpH, psbK-psbI, trnH-psbA |
| Isoetales | Isoetaceae | *Isoetes serracarajensis* J.B.S.Pereira, Salino & Stützel | ISO004-17 | Santos F. s.n. | Yes | Yes | Yes | ITS2, atpF-atpH, trnH-psbA |
| Isoetales | Isoetaceae | *Isoetes serracarajensis* J.B.S.Pereira, Salino & Stützel | ISO005-17 | Santos F. s.n. | Yes | Yes | Yes | ITS2, atpF-atpH, trnH-psbA |
| Isoetales | Isoetaceae | *Isoetes serracarajensis* J.B.S.Pereira, Salino & Stützel | ISO006-17 | Santos F. s.n. | Yes | Yes | Yes | ITS2, atpF-atpH, trnH-psbA |
| Isoetales | Isoetaceae | *Isoetes serracarajensis* J.B.S.Pereira, Salino & Stützel | ISO007-17 | Santos F. s.n. | Yes | Yes | Yes | ITS2, atpF-atpH, psbK-psbI, trnH-psbA |
| Isoetales | Isoetaceae | *Isoetes serracarajensis* J.B.S.Pereira, Salino & Stützel | ISO008-17 | Santos F. s.n. | Yes | Yes | Yes | ITS2, atpF-atpH, psbK-psbI, trnH-psbA |
| Isoetales | Isoetaceae | *Isoetes serracarajensis* J.B.S.Pereira, Salino & Stützel | ISO009-17 | Santos F. s.n. | Yes | Yes | Yes | ITS2, atpF-atpH, trnH-psbA |
| Isoetales | Isoetaceae | *Isoetes serracarajensis* J.B.S.Pereira, Salino & Stützel | ISO010-17 | Santos F. s.n. | Yes | Yes | Yes | ITS2, atpF-atpH, trnH-psbA |
| Isoetales | Isoetaceae | *Isoetes serracarajensis* J.B.S.Pereira, Salino & Stützel | ISO011-17 | Falcão B.F. 240 | Yes | Yes | Yes | ITS2, atpF-atpH |
| Isoetales | Isoetaceae | *Isoetes serracarajensis* J.B.S.Pereira, Salino & Stützel | ISO012-17 | Santos F. E2FLO.04 | Yes | Yes | Yes | ITS2, atpF-atpH |
| Isoetales | Isoetaceae | *Isoetes serracarajensis* J.B.S.Pereira, Salino & Stützel | ISO013-17 | Santos F. s.n. | Yes | Yes | Yes | atpF-atpH, trnH-psbA |
| Isoetales | Isoetaceae | *Isoetes serracarajensis* J.B.S.Pereira, Salino & Stützel | ISO014-17 | Santos F. s.n. | Yes | Yes | Yes | ITS2, atpF-atpH, psbK-psbI |
| Isoetales | Isoetaceae | *Isoetes serracarajensis* J.B.S.Pereira, Salino & Stützel | ISO015-17 | Falcão B.F. 241 | Yes | Yes | Yes | ITS2, atpF-atpH, psbK-psbI, trnH-psbA |
| Isoetales | Isoetaceae | *Isoetes serracarajensis* J.B.S.Pereira, Salino & Stützel | ISO016-17 | Falcão B.F. 242 | Yes | Yes | Yes | ITS2, trnH-psbA |
| Isoetales | Isoetaceae | *Isoetes serracarajensis* J.B.S.Pereira, Salino & Stützel | ISO017-17 | Santos F. E2FLO.10 | Yes | Yes | Yes | ITS2, atpF-atpH |
| Isoetales | Isoetaceae | *Isoetes serracarajensis* J.B.S.Pereira, Salino & Stützel | ISO018-17 | Falcão B.F. 243 | Yes | Yes | Yes | ITS2, psbK-psbI |
| Isoetales | Isoetaceae | *Isoetes serracarajensis* J.B.S.Pereira, Salino & Stützel | ISO019-17 | Santos F. E1TZ03MAR | Yes | Yes | Yes | ITS2, atpF-atpH, psbK-psbI, trnH-psbA |
| Isoetales | Isoetaceae | *Isoetes serracarajensis* J.B.S.Pereira, Salino & Stützel | ISO020-17 | Santos F. s.n. | Yes | Yes | Yes | ITS2 |
| Isoetales | Isoetaceae | *Isoetes serracarajensis* J.B.S.Pereira, Salino & Stützel | ISO021-17 | Santos F. E3BOC04MAR | Yes | Yes | Yes | ITS2, atpF-atpH, psbK-psbI, trnH-psbA |
| Isoetales | Isoetaceae | *Isoetes serracarajensis* J.B.S.Pereira, Salino & Stützel | ISO022-17 | Santos F. s.n. | Yes | Yes | Yes | ITS2, atpF-atpH |
| Isoetales | Isoetaceae | *Isoetes serracarajensis* J.B.S.Pereira, Salino & Stützel | ISO023-17 | Santos F. s.n. | Yes | Yes | Yes | ITS2, atpF-atpH, psbK-psbI, trnH-psbA |
| Isoetales | Isoetaceae | *Isoetes serracarajensis* J.B.S.Pereira, Salino & Stützel | ISO027-17 | Falcão B.F. 458 | Yes | No | Yes | ITS2, trnH-psbA |
| Isoetales | Isoetaceae | *Isoetes serracarajensis* J.B.S.Pereira, Salino & Stützel | ISO028-17 | Santos F. E3BOC19ABR | Yes | No | Yes | ITS2, trnH-psbA |
| Isoetales | Isoetaceae | *Isoetes serracarajensis* J.B.S.Pereira, Salino & Stützel | ISO029-17 | Falcão B.F. 460 | Yes | No | Yes | ITS2, trnH-psbA |
| Isoetales | Isoetaceae | *Isoetes serracarajensis* J.B.S.Pereira, Salino & Stützel | ISO030-17 | Santos F. s.n. | Yes | No | Yes | ITS2 |
| Isoetales | Isoetaceae | *Isoetes serracarajensis* J.B.S.Pereira, Salino & Stützel | ISO031-17 | Santos F. s.n. | Yes | No | Yes | ITS2 |
| Isoetales | Isoetaceae | *Isoetes serracarajensis* J.B.S.Pereira, Salino & Stützel | ISO032-17 | Santos F. s.n. | Yes | No | Yes | ITS2, trnH-psbA |
| Isoetales | Isoetaceae | *Isoetes serracarajensis* J.B.S.Pereira, Salino & Stützel | ISO033-17 | Santos F. s.n. | Yes | No | Yes | ITS2, trnH-psbA |
| Isoetales | Isoetaceae | *Isoetes serracarajensis* J.B.S.Pereira, Salino & Stützel | ISO034-17 | Santos F. s.n. | Yes | No | Yes | ITS2, trnH-psbA |
| Isoetales | Isoetaceae | *Isoetes serracarajensis* J.B.S.Pereira, Salino & Stützel | ISO035-17 | Santos F. s.n. | Yes | No | Yes | ITS2, trnH-psbA |
| Isoetales | Isoetaceae | *Isoetes serracarajensis* J.B.S.Pereira, Salino & Stützel | ISO036-17 | Santos F. s.n. | Yes | No | Yes | ITS2, trnH-psbA |
| Isoetales | Isoetaceae | *Isoetes serracarajensis* J.B.S.Pereira, Salino & Stützel | ISO037-17 | Falcão B.F. 459 | Yes | No | Yes | ITS2, trnH-psbA |
| Isoetales | Isoetaceae | *Isoetes serracarajensis* J.B.S.Pereira, Salino & Stützel | ISO038-17 | Santos F. s.n. | Yes | No | Yes | ITS2, trnH-psbA |
| Isoetales | Isoetaceae | *Isoetes serracarajensis* J.B.S.Pereira, Salino & Stützel | ISO039-17 | Santos F. s.n. | Yes | No | Yes | ITS2, trnH-psbA |
| Isoetales | Isoetaceae | *Isoetes serracarajensis* J.B.S.Pereira, Salino & Stützel | ISO040-17 | Santos F. s.n. | Yes | No | Yes | ITS2, trnH-psbA |
| Isoetales | Isoetaceae | *Isoetes serracarajensis* J.B.S.Pereira, Salino & Stützel | ISO041-17 | Santos F. s.n. | Yes | No | Yes | trnH-psbA |
| Isoetales | Isoetaceae | *Isoetes serracarajensis* J.B.S.Pereira, Salino & Stützel | ISO042-17 | Santos F. s.n. | Yes | No | Yes | ITS2, trnH-psbA |
| Isoetales | Isoetaceae | *Isoetes serracarajensis* J.B.S.Pereira, Salino & Stützel | ISO043-17 | Santos F. s.n. | Yes | No | Yes | ITS2, trnH-psbA |
| Isoetales | Isoetaceae | *Isoetes serracarajensis* J.B.S.Pereira, Salino & Stützel | ISO044-17 | Santos F. s.n. | Yes | No | Yes | ITS2, trnH-psbA |
| Isoetales | Isoetaceae | *Isoetes serracarajensis* J.B.S.Pereira, Salino & Stützel | ISO045-17 | Santos F. s.n. | Yes | No | Yes | ITS2, trnH-psbA |
| Isoetales | Isoetaceae | *Isoetes serracarajensis* J.B.S.Pereira, Salino & Stützel | ISO046-17 | Santos F. s.n. | Yes | No | Yes | ITS2 |
| Isoetales | Isoetaceae | *Isoetes serracarajensis* J.B.S.Pereira, Salino & Stützel | ISO047-17 | Vasconcelos L.V. 844 | Yes | No | Yes | ITS2 |
| Isoetales | Isoetaceae | *Isoetes serracarajensis* J.B.S.Pereira, Salino & Stützel | ISO081-17 | Harley R.M. 57506 | Yes | Yes | Yes | rbcL, ITS2 |
| Isoetales | Isoetaceae | *Isoetes serracarajensis* J.B.S.Pereira, Salino & Stützel | ISO084-17 | Santos F. E3BOC20ABR | Yes | No | Yes | ITS2, trnH-psbA |
| Isoetales | Isoetaceae | *Isoetes serracarajensis* J.B.S.Pereira, Salino & Stützel | ISO085-17 | Santos F. s.n. | Yes | No | Yes | ITS2 |
| Isoetales | Isoetaceae | *Isoetes serracarajensis* J.B.S.Pereira, Salino & Stützel | ISO086-17 | Santos F. s.n. | Yes | No | Yes | ITS2, trnH-psbA |
| Isoetales | Isoetaceae | *Isoetes serracarajensis* J.B.S.Pereira, Salino & Stützel | ISO087-17 | Santos F. s.n. | Yes | No | Yes | ITS2 |
| Isoetales | Isoetaceae | *Isoetes serracarajensis* J.B.S.Pereira, Salino & Stützel | ISO088-17 | Falcão B.F. 477 | Yes | No | Yes | ITS2, trnH-psbA |
| Isoetales | Isoetaceae | *Isoetes serracarajensis* J.B.S.Pereira, Salino & Stützel | ISO089-17 | Santos F. E1N401MAI | Yes | No | Yes | ITS2, trnH-psbA |
| Isoetales | Isoetaceae | *Isoetes serracarajensis* J.B.S.Pereira, Salino & Stützel | ISO090-17 | Santos F. s.n. | Yes | No | Yes | ITS2, trnH-psbA |
| Isoetales | Isoetaceae | *Isoetes serracarajensis* J.B.S.Pereira, Salino & Stützel | ISO091-17 | Santos F. s.n. | Yes | No | Yes | ITS2, trnH-psbA |
| Isoetales | Isoetaceae | *Isoetes serracarajensis* J.B.S.Pereira, Salino & Stützel | ISO092-17 | Santos F. s.n. | Yes | No | Yes | ITS2, trnH-psbA |
| Isoetales | Isoetaceae | *Isoetes serracarajensis* J.B.S.Pereira, Salino & Stützel | ISO093-17 | Santos F. s.n. | Yes | No | Yes | ITS2, trnH-psbA |
| Isoetales | Isoetaceae | *Isoetes serracarajensis* J.B.S.Pereira, Salino & Stützel | ISO094-17 | Santos F. s.n. | Yes | No | Yes | ITS2, trnH-psbA |
| Isoetales | Isoetaceae | *Isoetes serracarajensis* J.B.S.Pereira, Salino & Stützel | ISO095-17 | Santos F. s.n. | Yes | No | Yes | ITS2, trnH-psbA |
| Isoetales | Isoetaceae | *Isoetes serracarajensis* J.B.S.Pereira, Salino & Stützel | ISO096-17 | Santos F. s.n. | Yes | No | Yes | ITS2, trnH-psbA |
| Isoetales | Isoetaceae | *Isoetes serracarajensis* J.B.S.Pereira, Salino & Stützel | ISO097-17 | Santos F. s.n. | Yes | No | Yes | ITS2, trnH-psbA |
| Isoetales | Isoetaceae | *Isoetes serracarajensis* J.B.S.Pereira, Salino & Stützel | ISO098-17 | Falcão B.F. 479 | Yes | No | Yes | ITS2, trnH-psbA |
| Isoetales | Isoetaceae | *Isoetes serracarajensis* J.B.S.Pereira, Salino & Stützel | ISO099-17 | Santos F. E1N601MAI | Yes | No | Yes | ITS2 |
| Isoetales | Isoetaceae | *Isoetes serracarajensis* J.B.S.Pereira, Salino & Stützel | ISO100-17 | Santos F. s.n. | Yes | No | Yes | ITS2, trnH-psbA |
| Isoetales | Isoetaceae | *Isoetes serracarajensis* J.B.S.Pereira, Salino & Stützel | ISO101-17 | Santos F. s.n. | Yes | No | Yes | ITS2, trnH-psbA |
| Isoetales | Isoetaceae | *Isoetes serracarajensis* J.B.S.Pereira, Salino & Stützel | ISO102-17 | Santos F. s.n. | Yes | No | Yes | ITS2 |
| Isoetales | Isoetaceae | *Isoetes serracarajensis* J.B.S.Pereira, Salino & Stützel | ISO103-17 | Santos F. s.n. | Yes | No | Yes | ITS2, trnH-psbA |
| Isoetales | Isoetaceae | *Isoetes serracarajensis* J.B.S.Pereira, Salino & Stützel | ISO104-17 | Santos F. s.n. | Yes | No | Yes | ITS2, trnH-psbA |
| Isoetales | Isoetaceae | *Isoetes serracarajensis* J.B.S.Pereira, Salino & Stützel | ISO105-17 | Santos F. s.n. | Yes | No | Yes | ITS2, trnH-psbA |
| Isoetales | Isoetaceae | *Isoetes serracarajensis* J.B.S.Pereira, Salino & Stützel | ISO106-17 | Santos F. s.n. | Yes | No | Yes | ITS2, trnH-psbA |
| Isoetales | Isoetaceae | *Isoetes serracarajensis* J.B.S.Pereira, Salino & Stützel | ISO107-17 | Santos F. s.n. | Yes | No | Yes | ITS2 |
| Isoetales | Isoetaceae | *Isoetes serracarajensis* J.B.S.Pereira, Salino & Stützel | ISO108-17 | Santos F. s.n. | Yes | No | Yes | ITS2, trnH-psbA |
| Isoetales | Isoetaceae | *Isoetes serracarajensis* J.B.S.Pereira, Salino & Stützel | ISO109-17 | Santos F. s.n. | Yes | No | Yes | ITS2, trnH-psbA |
| Isoetales | Isoetaceae | *Isoetes serracarajensis* J.B.S.Pereira, Salino & Stützel | ISO110-17 | Santos F. s.n. | Yes | No | Yes | ITS2, trnH-psbA |
| Isoetales | Isoetaceae | *Isoetes serracarajensis* J.B.S.Pereira, Salino & Stützel | ISO111-17 | Santos F. s.n. | Yes | No | Yes | ITS2 |
| Isoetales | Isoetaceae | *Isoetes serracarajensis* J.B.S.Pereira, Salino & Stützel | ISO112-17 | Santos F. s.n. | Yes | No | Yes | ITS2, trnH-psbA |
| Isoetales | Isoetaceae | *Isoetes serracarajensis* J.B.S.Pereira, Salino & Stützel | ISO113-17 | Santos F. s.n. | Yes | No | Yes | ITS2, trnH-psbA |
| Isoetales | Isoetaceae | *Isoetes serracarajensis* J.B.S.Pereira, Salino & Stützel | ISO114-17 | Santos F. s.n. | Yes | No | Yes | ITS2 |
| Isoetales | Isoetaceae | *Isoetes serracarajensis* J.B.S.Pereira, Salino & Stützel | ISO115-17 | Falcão B.F. 480 | Yes | No | Yes | ITS2, trnH-psbA |
| Isoetales | Isoetaceae | *Isoetes serracarajensis* J.B.S.Pereira, Salino & Stützel | ISO116-17 | Santos F. E3N701MAI | Yes | No | Yes | ITS2, trnH-psbA |
| Isoetales | Isoetaceae | *Isoetes serracarajensis* J.B.S.Pereira, Salino & Stützel | ISO117-17 | Santos F. s.n. | Yes | No | Yes | ITS2, trnH-psbA |
| Isoetales | Isoetaceae | *Isoetes serracarajensis* J.B.S.Pereira, Salino & Stützel | ISO118-17 | Santos F. s.n. | Yes | No | Yes | ITS2 |
| Isoetales | Isoetaceae | *Isoetes serracarajensis* J.B.S.Pereira, Salino & Stützel | ISO119-17 | Santos F. s.n. | Yes | No | Yes | ITS2, trnH-psbA |
| Isoetales | Isoetaceae | *Isoetes serracarajensis* J.B.S.Pereira, Salino & Stützel | ISO127-17 | Falcão B.F. 518 | Yes | No | Yes | ITS2, trnH-psbA |
| Isoetales | Isoetaceae | *Isoetes serracarajensis* J.B.S.Pereira, Salino & Stützel | ISO128-17 | Santos F. E2S11B01MAI | Yes | No | Yes | ITS2, trnH-psbA |
| Isoetales | Isoetaceae | *Isoetes serracarajensis* J.B.S.Pereira, Salino & Stützel | ISO129-17 | Santos F. s.n. | Yes | No | Yes | ITS2, trnH-psbA |
| Isoetales | Isoetaceae | *Isoetes serracarajensis* J.B.S.Pereira, Salino & Stützel | ISO130-17 | Santos F. s.n. | Yes | No | Yes | ITS2, trnH-psbA |
| Isoetales | Isoetaceae | *Isoetes serracarajensis J.B.S.Pereira, Salino & Stützel* | ISO131-17 | Santos F. s.n. | Yes | No | Yes | ITS2 |
| Isoetales | Isoetaceae | *Isoetes serracarajensis* J.B.S.Pereira, Salino & Stützel | ISO132-17 | Santos F. s.n. | Yes | No | Yes | ITS2, trnH-psbA |
| Isoetales | Isoetaceae | *Isoetes serracarajensis* J.B.S.Pereira, Salino & Stützel | ISO133-17 | Santos F. s.n. | Yes | No | Yes | ITS2, trnH-psbA |
| Isoetales | Isoetaceae | *Isoetes serracarajensis* J.B.S.Pereira, Salino & Stützel | ISO134-17 | Falcão B.F. 532 | Yes | No | Yes | ITS2, trnH-psbA |
| Isoetales | Isoetaceae | *Isoetes serracarajensis* J.B.S.Pereira, Salino & Stützel | ISO135-17 | Santos F. E2TZ01MAI | Yes | No | Yes | ITS2, trnH-psbA |
| Isoetales | Isoetaceae | *Isoetes serracarajensis* J.B.S.Pereira, Salino & Stützel | ISO136-17 | Santos F. s.n. | Yes | No | Yes | ITS2, trnH-psbA |
| Isoetales | Isoetaceae | *Isoetes serracarajensis* J.B.S.Pereira, Salino & Stützel | ISO137-17 | Santos F. s.n. | Yes | No | Yes | ITS2, trnH-psbA |
| Isoetales | Isoetaceae | *Isoetes serracarajensis* J.B.S.Pereira, Salino & Stützel | ISO138-17 | Santos F. s.n. | Yes | No | Yes | ITS2, trnH-psbA |
| Isoetales | Isoetaceae | *Isoetes serracarajensis* J.B.S.Pereira, Salino & Stützel | ISO139-17 | Santos F. s.n. | Yes | No | Yes | ITS2, trnH-psbA |
| Isoetales | Isoetaceae | *Isoetes serracarajensis* J.B.S.Pereira, Salino & Stützel | ISO140-17 | Santos F. s.n. | Yes | No | Yes | ITS2, trnH-psbA |
| Isoetales | Isoetaceae | *Isoetes serracarajensis* J.B.S.Pereira, Salino & Stützel | ISO141-17 | Falcão B.F. 571 | Yes | No | Yes | ITS2, trnH-psbA |
| Isoetales | Isoetaceae | *Isoetes serracarajensis* J.B.S.Pereira, Salino & Stützel | ISO142-17 | Santos F. E3CRI13MAI | Yes | No | Yes | ITS2, trnH-psbA |
| Isoetales | Isoetaceae | *Isoetes serracarajensis* J.B.S.Pereira, Salino & Stützel | ISO143-17 | Santos F. s.n. | Yes | No | Yes | ITS2, trnH-psbA |
| Isoetales | Isoetaceae | *Isoetes serracarajensis* J.B.S.Pereira, Salino & Stützel | ISO144-17 | Santos F. s.n. | Yes | No | Yes | ITS2, trnH-psbA |
| Isoetales | Isoetaceae | *Isoetes serracarajensis* J.B.S.Pereira, Salino & Stützel | ISO145-17 | Santos F. s.n. | Yes | No | Yes | ITS2, trnH-psbA |
| Isoetales | Isoetaceae | *Isoetes serracarajensis* J.B.S.Pereira, Salino & Stützel | ISO146-17 | Santos F. s.n. | Yes | No | Yes | ITS2, trnH-psbA |
| Isoetales | Isoetaceae | *Isoetes serracarajensis* J.B.S.Pereira, Salino & Stützel | ISO147-17 | Santos F. s.n. | Yes | No | Yes | ITS2 |
| Isoetales | Isoetaceae | *Isoetes serracarajensis* J.B.S.Pereira, Salino & Stützel | ISO148-17 | Santos F. s.n. | Yes | No | Yes | ITS2, trnH-psbA |
| Isoetales | Isoetaceae | *Isoetes serracarajensis* J.B.S.Pereira, Salino & Stützel | ISO149-17 | Santos F. s.n. | Yes | No | Yes | ITS2, trnH-psbA |
| Isoetales | Isoetaceae | *Isoetes serracarajensis* J.B.S.Pereira, Salino & Stützel | ISO150-17 | Santos F. s.n. | Yes | No | Yes | ITS2, trnH-psbA |
| Lamiales | Acanthaceae | *Justicia distichophylla* F.A.Silva, A.Gil & Kameyama | CANGI005-17 | Harley R.M. 57454 | No | Yes | Yes | rbcL, trnH-psbA |
| Lamiales | Acanthaceae | *Justicia distichophylla* F.A.Silva, A.Gil & Kameyama | CANGI001-17 | Vasconcelos L.V. 778 | No | Yes | Yes | rbcL, trnH-psbA |
| Lamiales | Acanthaceae | *Justicia potamogeton* Lindau | CANGA356-17 | Harley R.M. 57245 | Yes | Yes | Yes | matK, rpoC1, atpF-atpH |
| Lamiales | Acanthaceae | *Justicia* sp.3 | CANGI006-17 | Harley R.M. 58067 | Yes | No | Yes | rbcL, ITS2 |
| Lamiales | Acanthaceae | *Justicia* sp.5 | CANGI004-17 | Harley R.M. 57319 | Yes | Yes | Yes | rbcL, ITS2, matK, rpoC1, atpF-atpH |
| Lamiales | Acanthaceae | *Justicia sprucei* V.A.W.Graham | CANGA408-17 | Cardoso A. 1956 | Yes | Yes | Yes | rbcL, matK |
| Lamiales | Acanthaceae | *Ruellia anamariae* A.S.Reis, A.Gil & C. Kameyama | CANGI003-17 | Trindade J.R. 221 | Yes | Yes | Yes | rbcL, ITS2, matK, rpoC1, atpF-atpH, psbK-psbI |
| Lamiales | Acanthaceae | *Ruellia anamariae* A.S.Reis, A.Gil & C. Kameyama | CANGA458-20 | Harley R.M. 57860 | Yes | No | Yes | rbcL, ITS2 |
| Lamiales | Acanthaceae | *Ruellia anamariae* A.S.Reis, A.Gil & C. Kameyama | CGII668-20 | Zappi D.C. 4511 | Yes | No | Yes | rbcL, ITS2 |
| Lamiales | Acanthaceae | *Ruellia inflata* Rich. | CANGI002-17 | Harley R.M. 54243 | Yes | Yes | No | rbcL, ITS2, matK, rpoB, rpoC1, atpF-atpH |
| Lamiales | Acanthaceae | *Ruellia inflata* Rich. | CANGA237-17 | Gil A. 504 | Yes | Yes | No | rbcL, ITS2, matK, rpoB, atpF-atpH |
| Lamiales | Acanthaceae | *Ruellia wurdackii* Wasshausen | CANGA435-17 | Harley R.M. 57322 | Yes | Yes | Yes | ITS2 |
| Lamiales | Bignoniaceae | *Adenocalymma bracteosum* (DC.) L.G.Lohmann | CANGI066-17 | Harley R.M. 57935 | No | No | Yes | rbcL |
| Lamiales | Bignoniaceae | *Adenocalymma magnificum* Mart. ex DC. | CANGI342-17 | Mota N.F.O. 3438 | No | Yes | Yes | rbcL, rpoB, rpoC1, atpF-atpH |
| Lamiales | Bignoniaceae | *Amphilophium mansoanum* (DC.) L.G.Lohmann | CANGI067-17 | Harley R.M. 57520 | Yes | Yes | Yes | rbcL, ITS2 |
| Lamiales | Bignoniaceae | *Anemopaegma carajasense* A.H. Gentry ex Firetti-Leggieri & L.G. Lohmann | CANGI068-17 | Lopes C.S.A. 9 | Yes | Yes | Yes | ITS2, matK, rpoC1, atpF-atpH, psbK-psbI |
| Lamiales | Bignoniaceae | *Anemopaegma carajasense* A.H. Gentry ex Firetti-Leggieri & L.G. Lohmann | CANGI080-17 | Vasconcelos L.V. 1147 | Yes | No | Yes | rbcL, ITS2 |
| Lamiales | Bignoniaceae | *Anemopaegma carajasense* A.H. Gentry ex Firetti-Leggieri & L.G. Lohmann | CANGI082-17 | Harley R.M. 58133 | Yes | No | Yes | rbcL, ITS2 |
| Lamiales | Bignoniaceae | *Bignonia corymbosa* (Vent.) L.G.Lohmann | CANGI077-17 | Praia T.S. 18 | Yes | Yes | Yes | ITS2, rpoB, rpoC1, psbK-psbI |
| Lamiales | Bignoniaceae | *Bignonia corymbosa* (Vent.) L.G.Lohmann | CANGI079-17 | Vasconcelos L.V. 1106 | Yes | No | Yes | rbcL, ITS2 |
| Lamiales | Bignoniaceae | *Bignonia corymbosa* (Vent.) L.G.Lohmann | CANGI081-17 | Harley R.M. 58130 | Yes | No | Yes | rbcL, ITS2 |
| Lamiales | Bignoniaceae | *Bignonia prieurii* DC. | CANGI072-17 | Vasconcelos L.V. 932 | No | No | Yes | rbcL, ITS2 |
| Lamiales | Bignoniaceae | *Cuspidaria lateriflora* (Mart.) DC. | CANGA251-17 | Harley R.M. 57924A | No | No | Yes | rbcL, ITS2 |
| Lamiales | Bignoniaceae | *Fridericia cinnamomea* (DC.) L.G.Lohmann | CANGI069-17 | Harley R.M. 57300 | Yes | Yes | Yes | rbcL, ITS2, matK, rpoB, rpoC1, atpF-atpH, psbK-psbI |
| Lamiales | Bignoniaceae | *Fridericia cinnamomea* (DC.) L.G.Lohmann | CANGI075-17 | Trindade J.R. 361 | Yes | Yes | Yes | matK |
| Lamiales | Bignoniaceae | *Lundia corymbifera* (Vahl) Sandwith | CANGI629-17 | Harley R.M. 58159 | No | No | Yes | rbcL, ITS2 |
| Lamiales | Bignoniaceae | *Pachyptera incarnata* (Aubl.) Francisco & L.G.Lohmann | CANGI074-17 | Viana P.L. 5761 | Yes | Yes | Yes | rbcL, ITS2, matK, atpF-atpH, psbK-psbI |
| Lamiales | Bignoniaceae | *Pleonotoma melioides* (S.Moore) A.H.Gentry | CANGA285-17 | Reis A.S. 20 | Yes | Yes | Yes | rbcL, ITS2, rpoB, rpoC1, atpF-atpH, psbK-psbI |
| Lamiales | Bignoniaceae | *Pleonotoma orientalis* Sandwith | CANGI076-17 | Praia T.S. 25 | Yes | Yes | Yes | rbcL, rpoC1, atpF-atpH |
| Lamiales | Gesneriaceae | *Codonanthopsis calcarata* (Miq.) Chautems & Mat. Perret | CANGI202-17 | Mota N.F.O. 3441 | No | Yes | Yes | rbcL, ITS2, atpF-atpH, trnH-psbA |
| Lamiales | Gesneriaceae | *Codonanthopsis calcarata* (Miq.) Chautems & Mat. Perret | CANGA444-20 | Mota N.F.O. 3447 | No | Yes | Yes | rbcL, ITS2, atpF-atpH, psbK-psbI |
| Lamiales | Gesneriaceae | *Gloxinia erinoides* (DC.) Roalson & Boggan | CGII613-20 | Zappi D.C. 4293 | No | No | No | ITS2 |
| Lamiales | Gesneriaceae | *Phinaea albolineata* (Hook.) Benth. ex Hemsl. | CANGI264-17 | Pastore M. 539 | Yes | No | No | rbcL, ITS2 |
| Lamiales | Gesneriaceae | *Sinningia minima* A.O.Araujo & Chautems | CANGA296-17 | Viana P.L. 5572 | Yes | Yes | Yes | rpoB |
| Lamiales | Gesneriaceae | *Sinningia rupicola* (Mart.) Wiehler | ITVGA005-17 | Nogueira M.G.C. 457 | No | Yes | No | rbcL, ITS2, matK, rpoB |
| Lamiales | Lamiaceae | *Aegiphila integrifolia* (Jacq.) Moldenke | CANGI281-17 | Cardoso A. 1978 | Yes | Yes | Yes | rbcL, ITS2, matK, rpoC1, atpF-atpH, trnH-psbA |
| Lamiales | Lamiaceae | *Aegiphila integrifolia* (Jacq.) Moldenke | CANGA241-17 | Mota N.F.O. 3405 | Yes | Yes | Yes | rbcL, ITS2, matK, rpoC1, atpF-atpH |
| Lamiales | Lamiaceae | *Aegiphila integrifolia* (Jacq.) Moldenke | CANGA242-17 | Harley R.M. 57415 | Yes | Yes | Yes | rbcL, ITS2, matK, rpoB, rpoC1, atpF-atpH, psbK-psbI |
| Lamiales | Lamiaceae | *Aegiphila integrifolia* (Jacq.) Moldenke | CANGI280-17 | Harley R.M. 58060 | Yes | No | Yes | rbcL, ITS2 |
| Lamiales | Lamiaceae | *Amasonia campestris* (Aubl.) Moldenke | CANGA250-17 | Harley R.M. 57481 | Yes | Yes | Yes | rbcL, ITS2 |
| Lamiales | Lamiaceae | *Amasonia lasiocaulos* Mart. & Schauer ex Schauer | CANGI282-17 | Pastore M. 370 | Yes | Yes | Yes | rbcL, ITS2, trnH-psbA |
| Lamiales | Lamiaceae | *Cantinoa mutabilis* (Rich.) Harley & J.F.B.Pastore | CANGA297-17 | Harley R.M. 57320 | Yes | Yes | Yes | rbcL, ITS2 |
| Lamiales | Lamiaceae | *Hyptis atrorubens* Poit. | CANGI283-17 | Vasconcelos L.V. 789 | Yes | Yes | No | rbcL, ITS2 |
| Lamiales | Lamiaceae | *Hyptis atrorubens* Poit. | CANGI285-17 | Harley R.M. 58075 | Yes | No | No | rbcL, ITS2 |
| Lamiales | Lamiaceae | *Hyptis parkeri* Benth. | CANGA337-17 | Harley R.M. 57956 | Yes | Yes | Yes | rbcL, ITS2 |
| Lamiales | Lamiaceae | *Hyptis recurvata* Poit. | CANGA373-17 | Harley R.M. 58068 | Yes | No | No | rbcL, ITS2 |
| Lamiales | Lamiaceae | *Marsypianthes chamaedrys* (Vahl) Kuntze | CANGI305-17 | Praia T.S. 8 | Yes | Yes | No | rbcL, rpoC1 |
| Lamiales | Lamiaceae | *Mesosphaerum pectinatum* (L.) Kuntze | CANGI284-17 | Harley R.M. 57311 | Yes | Yes | Yes | ITS2 |
| Lamiales | Lamiaceae | *Vitex triflora* Vahl | ITVRT001-17 | Harley R.M. 57265 | Yes | Yes | No | ITS2, rpoB, rpoC1 |
| Lamiales | Lamiaceae | *Vitex triflora* Vahl | CANGA425-17 | Harley R.M. 57983 | Yes | Yes | No | rbcL, ITS2 |
| Lamiales | Lamiaceae | *Vitex triflora* Vahl | CANGA426-17 | Harley R.M. 57915 | Yes | No | No | rbcL |
| Lamiales | Lentibulariaceae | *Utricularia calycifida* Benj. | CANGA048-17 | Harley R.M. 57251 | Yes | Yes | No | rbcL, ITS2, matK, rpoC1 |
| Lamiales | Lentibulariaceae | *Utricularia calycifida* Benj. | CANGA049-17 | Meirelles J. 964 | Yes | Yes | No | rbcL, ITS2 |
| Lamiales | Lentibulariaceae | *Utricularia neottioides* A.St.-Hil. & Girard | CANGA316-17 | Vasconcelos L.V. 799 | Yes | Yes | Yes | rbcL, ITS2 |
| Lamiales | Lentibulariaceae | *Utricularia physoceras* P.Taylor | CANGA349-17 | Harley R.M. 57304 | Yes | Yes | Yes | rbcL, ITS2, matK, rpoB, rpoC1, atpF-atpH |
| Lamiales | Lentibulariaceae | *Utricularia physoceras* P.Taylor | CANGA352-17 | Vasconcelos L.V. 804 | Yes | Yes | Yes | rbcL, ITS2 |
| Lamiales | Lentibulariaceae | *Utricularia physoceras* P.Taylor | CANGA353-17 | Harley R.M. 57496 | Yes | Yes | Yes | rbcL, ITS2 |
| Lamiales | Lentibulariaceae | *Utricularia physoceras* P.Taylor | CANGI153-17 | Harley R.M. 58106 | Yes | No | Yes | rbcL |
| Lamiales | Lentibulariaceae | *Utricularia pusilla* Vahl | CANGI293-17 | Vasconcelos L.V. 759 | Yes | Yes | Yes | rbcL, ITS2 |
| Lamiales | Lentibulariaceae | *Utricularia pusilla* Vahl | CANGI294-17 | Vasconcelos L.V. 805 | Yes | Yes | Yes | rbcL, ITS2 |
| Lamiales | Lentibulariaceae | *Utricularia subulata* L. | CANGI296-17 | Harley R.M. 58112 | Yes | No | No | rbcL, ITS2 |
| Lamiales | Linderniaceae | *Lindernia brachyphylla* Pennell | CANGA035-17 | Mota N.F.O. 3433 | Yes | Yes | Yes | rbcL, ITS2, matK, rpoB, atpF-atpH |
| Lamiales | Linderniaceae | *Lindernia brachyphylla* Pennell | CANGI300-17 | Vasconcelos L.V. 807 | Yes | Yes | Yes | rbcL, ITS2, trnH-psbA |
| Lamiales | Linderniaceae | *Lindernia diffusa* (L.) Wettst. | CANGA124-17 | Mota N.F.O. 3431 | Yes | Yes | No | rbcL, ITS2, matK, rpoB, rpoC1 |
| Lamiales | Orobanchaceae | *Agalinis hispidula* (Mart.) D'Arcy | CGII616-20 | Zappi D.C. 4307 | No | No | Yes | rbcL, ITS2 |
| Lamiales | Orobanchaceae | *Buchnera carajasensis* Scatigna & N.Mota | CANGI409-17 | Viana P.L. 5625 | Yes | Yes | Yes | rbcL, ITS2, matK, rpoC1, atpF-atpH, psbK-psbI, trnH-psbA |
| Lamiales | Orobanchaceae | *Buchnera carajasensis* Scatigna & N.Mota | CANGI411-17 | Harley R.M. 57438 | Yes | Yes | Yes | rbcL, ITS2 |
| Lamiales | Orobanchaceae | *Buchnera carajasensis* Scatigna & N.Mota | CANGI412-17 | Vasconcelos L.V. 816 | Yes | Yes | Yes | rbcL, ITS2 |
| Lamiales | Orobanchaceae | *Buchnera carajasensis* Scatigna & N.Mota | CANGI413-17 | Pastore M. 352 | Yes | Yes | Yes | rbcL, ITS2 |
| Lamiales | Orobanchaceae | *Buchnera carajasensis* Scatigna & N.Mota | CANGI624-17 | Pastore M. 610 | Yes | No | Yes | ITS2 |
| Lamiales | Orobanchaceae | *Buchnera carajasensis* Scatigna & N.Mota | CANGI414-17 | Pastore M. 646 | Yes | No | Yes | rbcL, ITS2 |
| Lamiales | Orobanchaceae | *Buchnera carajasensis* Scatigna & N.Mota | CANGI415-17 | Rocha K. 85 | Yes | Yes | Yes | rbcL, ITS2 |
| Lamiales | Orobanchaceae | *Buchnera palustris* (Aubl.) Spreng. | CGII614-20 | Zappi D.C. 4294 | No | No | Yes | rbcL, ITS2 |
| Lamiales | Plantaginaceae | *Bacopa monnierioides* (Cham.) B.L.Rob. | CANGA298-17 | Mota N.F.O. 3432 | Yes | Yes | No | rbcL, ITS2, matK, rpoC1, atpF-atpH, psbK-psbI |
| Lamiales | Plantaginaceae | *Bacopa monnierioides* (Cham.) B.L.Rob. | CANGA299-17 | Mota N.F.O. 3412 | Yes | Yes | No | rbcL, ITS2, matK, rpoB, rpoC1, atpF-atpH, psbK-psbI |
| Lamiales | Plantaginaceae | *Bacopa myriophylloides* (Benth.) Wettst. | CANGA307-17 | Mota N.F.O. 3407 | Yes | Yes | Yes | rbcL, ITS2, atpF-atpH, psbK-psbI |
| Lamiales | Plantaginaceae | *Bacopa reflexa* (Benth.) Edwall | CANGI297-17 | Harley R.M. 57458 | Yes | Yes | Yes | rbcL, ITS2, trnH-psbA |
| Lamiales | Plantaginaceae | *Bacopa reflexa* (Benth.) Edwall | CANGI299-17 | Vasconcelos L.V. 784 | Yes | Yes | Yes | rbcL, ITS2 |
| Lamiales | Plantaginaceae | *Bacopa reflexa* (Benth.) Edwall | CANGI301-17 | Vasconcelos L.V. 827 | Yes | Yes | Yes | rbcL, ITS2 |
| Lamiales | Verbenaceae | *Lantana paraensis* (Moldenke) R.W.Sanders. | CANGI611-17 | Nogueira M.G.C. 680 | Yes | No | Yes | rbcL, ITS2 |
| Lamiales | Verbenaceae | *Lantana paraensis* (Moldenke) R.W.Sanders. | CANGI604-17 | Pastore M. 567 | Yes | No | Yes | rbcL, ITS2 |
| Lamiales | Verbenaceae | *Lantana paraensis* (Moldenke) R.W.Sanders. | CANGI605-17 | Pastore M. 575 | Yes | No | Yes | rbcL, ITS2 |
| Lamiales | Verbenaceae | *Lippia grata* Schauer | CANGI606-17 | Viana P.L. 5569 | Yes | Yes | Yes | rbcL, ITS2, matK, rpoC1, atpF-atpH, psbK-psbI, trnH-psbA |
| Lamiales | Verbenaceae | *Lippia grata* Schauer | CANGI603-17 | Vasconcelos L.V. 773 | Yes | Yes | Yes | rbcL, ITS2 |
| Lamiales | Verbenaceae | *Lippia grata* Schauer | CANGA324-17 | Viana P.L. 6114 | Yes | Yes | Yes | rbcL, ITS2 |
| Lamiales | Verbenaceae | *Lippia grata* Schauer | CANGA325-17 | Viana P.L. 6207 | Yes | Yes | Yes | rbcL, ITS2 |
| Lamiales | Verbenaceae | *Lippia grata* Schauer | CANGI607-17 | Harley R.M. 58120 | Yes | No | Yes | rbcL, ITS2 |
| Lamiales | Verbenaceae | *Priva lappulacea* (L.) Pers. | CANGA249-17 | Praia T.S. 9 | No | Yes | No | ITS2, matK, rpoB, rpoC1, atpF-atpH, trnH-psbA |
| Lamiales | Verbenaceae | *Stachytarpheta cayennensis* (Rich.) Vahl | CANGI609-17 | Prado M.L 367 | Yes | Yes | No | rbcL, ITS2, rpoB, rpoC1, atpF-atpH, psbK-psbI |
| Lamiales | Verbenaceae | *Stachytarpheta cayennensis* (Rich.) Vahl | CANGI608-17 | Rocha K. 55 | Yes | Yes | No | rbcL, rpoC1, atpF-atpH |
| Lamiales | Verbenaceae | *Stachytarpheta cayennensis* (Rich.) Vahl | CANGI610-17 | Nogueira M.G.C. 679 | Yes | No | No | rbcL, ITS2 |
| Lamiales | Verbenaceae | *Stachytarpheta glabra* Cham. | ITVGA001-17 | Harley R.M. 57063 | No | Yes | Yes | atpF-atpH |
| Laurales | Lauraceae | *Aiouea myristicoides* Mez | CANGI286-17 | Harley R.M. 57911 | Yes | No | Yes | rbcL |
| Laurales | Lauraceae | *Cassytha filiformis* L. | CANGI287-17 | Lopes C.S.A. 1 | Yes | Yes | No | rbcL, matK, rpoB, rpoC1, trnH-psbA |
| Laurales | Lauraceae | *Cassytha filiformis* L. | CANGI288-17 | Viana P.L. 6102 | Yes | Yes | No | rbcL |
| Laurales | Lauraceae | *Cassytha filiformis* L. | CANGI289-17 | Pastore M. 360 | Yes | Yes | No | rbcL, ITS2 |
| Laurales | Lauraceae | *Dicypellium caryophyllaceum* (Mart.) Nees | CANGI291-17 | Viana P.L. 6100 | No | Yes | No | rbcL, ITS2 |
| Laurales | Lauraceae | *Mezilaurus ita-uba* (Meisn.) Taub. ex Mez | CANGI290-17 | Harley R.M. 57423 | Yes | Yes | Yes | rbcL, ITS2, matK, psbK-psbI, trnH-psbA |
| Laurales | Lauraceae | *Nectandra cuspidata* Nees | CANGI292-17 | Nogueira M.G.C. 672 | Yes | No | No | rbcL |
| Laurales | Lauraceae | *Nectandra cuspidata* Nees | CANGI626-17 | Harley R.M. 58077 | Yes | No | No | ITS2 |
| Laurales | Siparunaceae | *Siparuna ficoides* Renner & Hausner | CANGI581-17 | Harley R.M. 57426 | Yes | Yes | Yes | rbcL, matK, rpoB, atpF-atpH, psbK-psbI |
| Laurales | Siparunaceae | *Siparuna ficoides* Renner & Hausner | CANGI582-17 | Harley R.M. 57940 | Yes | No | Yes | rbcL |
| Liliales | Smilacaceae | *Smilax fluminensis* Steud. | CANGI592-17 | Pastore M. 509 | No | No | No | rbcL |
| Liliales | Smilacaceae | *Smilax irrorata* Mart. ex Griseb. | CANGI583-17 | Harley R.M. 57330 | Yes | Yes | Yes | rbcL |
| Liliales | Smilacaceae | *Smilax irrorata* Mart. ex Griseb. | CANGI584-17 | Viana P.L. 5765 | Yes | Yes | Yes | rbcL, ITS2, matK, rpoC1 |
| Liliales | Smilacaceae | *Smilax irrorata* Mart. ex Griseb. | CANGI586-17 | Lopes C.S.A. 10 | Yes | Yes | Yes | rbcL, rpoC1, trnH-psbA |
| Liliales | Smilacaceae | *Smilax irrorata* Mart. ex Griseb. | CANGI587-17 | Dias C.S.P. 12 | Yes | Yes | Yes | matK, rpoB, rpoC1 |
| Liliales | Smilacaceae | *Smilax irrorata* Mart. ex Griseb. | CANGI588-17 | Vasconcelos L.V. 877 | Yes | Yes | Yes | rbcL |
| Liliales | Smilacaceae | *Smilax irrorata* Mart. ex Griseb. | CANGI589-17 | Viana P.L. 6122 | Yes | Yes | Yes | rbcL |
| Liliales | Smilacaceae | *Smilax irrorata* Mart. ex Griseb. | CANGI590-17 | Harley R.M. 58000 | Yes | Yes | Yes | rbcL |
| Liliales | Smilacaceae | *Smilax irrorata* Mart. ex Griseb. | CANGI591-17 | Harley R.M. 57885 | Yes | No | Yes | rbcL |
| Lycopodiales | Lycopodiaceae | *Palhinhaea cernua* (L.) Vasc. & Franco | CANGI395-17 | Harley R.M. 57891 | Yes | No | Yes | rbcL |
| Magnoliales | Annonaceae | *Cymbopetalum brasiliense* (Vell.) Benth. ex Baill. | CANGI016-17 | Harley R.M. 57259 | No | Yes | No | rbcL, ITS2, matK, rpoC1, atpF-atpH, psbK-psbI |
| Magnoliales | Annonaceae | *Guatteria punctata* (Aubl.) R.A.Howard | CANGI015-17 | Harley R.M. 58074 | Yes | No | No | rbcL |
| Magnoliales | Annonaceae | *Xylopia aromatica* (Lam.) Mart. | CANGA025-17 | Costa J.L.C. 10 | Yes | Yes | No | rbcL, matK, rpoB, rpoC1, atpF-atpH, psbK-psbI, trnH-psbA |
| Magnoliales | Myristicaceae | *Virola sebifera* Aubl. | CANGA471-20 | Giulietti A.M. 2677 | No | No | No | rbcL |
| Malpighiales | Calophyllaceae | *Calophyllum brasiliense* Cambess. | CANGA042-17 | Harley R.M. 57888 | Yes | No | No | rbcL |
| Malpighiales | Calophyllaceae | *Calophyllum brasiliense* Cambess. | CANGA041-17 | Vasconcelos L.V. 916 | Yes | No | No | ITS2 |
| Malpighiales | Chrysobalanaceae | *Hirtella pilosissima* Mart. & Zucc. | CANGI101-17 | Harley R.M. 57242 | Yes | Yes | Yes | rbcL, ITS2, rpoB, rpoC1, atpF-atpH, psbK-psbI |
| Malpighiales | Chrysobalanaceae | *Hirtella racemosa* Lam. | CANGI102-17 | Harley R.M. 58172 | Yes | No | No | rbcL |
| Malpighiales | Chrysobalanaceae | *Moquilea egleri* (Prance) Sothers & Prance | CANGA167-17 | Viana P.L. 5780 | Yes | Yes | Yes | rbcL, ITS2, matK, rpoC1 |
| Malpighiales | Clusiaceae | *Clusia* aff. *weddelliana* Planch. & Triana | CANGI105-17 | Gil A. 515 | Yes | Yes | No | rbcL, ITS2 |
| Malpighiales | Clusiaceae | *Clusia* aff. *weddelliana* Planch. & Triana | CANGA326-17 | Harley R.M. 57897 | Yes | No | No | rbcL, ITS2 |
| Malpighiales | Clusiaceae | *Clusia panapanari* (Aubl.) Choisy | CANGI104-17 | Gil A. 512 | Yes | Yes | No | rbcL, ITS2 |
| Malpighiales | Clusiaceae | *Clusia panapanari* (Aubl.) Choisy | CANGA327-17 | Harley R.M. 57879 | Yes | No | No | rbcL |
| Malpighiales | Erythroxylaceae | *Erythroxylum carajasense* (Plowman) Costa-Lima | CANGA260-17 | Vasconcelos L.V. 820 | Yes | Yes | Yes | rbcL, ITS2 |
| Malpighiales | Erythroxylaceae | *Erythroxylum carajasense* (Plowman) Costa-Lima | CANGA261-17 | Vasconcelos L.V. 1061 | Yes | No | Yes | ITS2 |
| Malpighiales | Erythroxylaceae | *Erythroxylum carajasense* (Plowman) Costa-Lima | CANGA259-17 | Vasconcelos L.V. 1101 | Yes | No | Yes | rbcL, ITS2 |
| Malpighiales | Erythroxylaceae | *Erythroxylum citrifolium* A.St.-Hil. | CANGI190-17 | Harley R.M. 57135 | Yes | Yes | No | rbcL, ITS2, rpoB, atpF-atpH |
| Malpighiales | Erythroxylaceae | *Erythroxylum citrifolium* A.St.-Hil. | CANGI198-17 | Trindade J.R. 352 | Yes | Yes | No | ITS2, rpoC1 |
| Malpighiales | Erythroxylaceae | *Erythroxylum citrifolium* A.St.-Hil. | CANGI192-17 | Dias C.S.P. 11 | Yes | Yes | No | rbcL |
| Malpighiales | Erythroxylaceae | *Erythroxylum citrifolium* A.St.-Hil. | CANGI195-17 | Harley R.M. 57981 | Yes | Yes | No | rbcL, ITS2 |
| Malpighiales | Erythroxylaceae | *Erythroxylum citrifolium* A.St.-Hil. | CANGA127-17 | Vasconcelos L.V. 1034 | Yes | No | No | ITS2 |
| Malpighiales | Erythroxylaceae | *Erythroxylum nelson-rosae* Plowman | CANGI191-17 | Harley R.M. 57339 | Yes | Yes | Yes | rbcL, rpoC1 |
| Malpighiales | Erythroxylaceae | *Erythroxylum nelson-rosae* Plowman | CANGA308-17 | Harley R.M. 57453 | Yes | Yes | Yes | rbcL, ITS2 |
| Malpighiales | Erythroxylaceae | *Erythroxylum nelson-rosae* Plowman | CANGA309-17 | Vasconcelos L.V. 829 | Yes | Yes | Yes | rbcL, ITS2 |
| Malpighiales | Erythroxylaceae | *Erythroxylum nelson-rosae* Plowman | CANGA310-17 | Vasconcelos L.V. 1047 | Yes | No | Yes | rbcL, ITS2 |
| Malpighiales | Erythroxylaceae | *Erythroxylum nelson-rosae* Plowman | CANGA311-17 | Vasconcelos L.V. 1048 | Yes | No | Yes | rbcL, ITS2 |
| Malpighiales | Erythroxylaceae | *Erythroxylum nelson-rosae* Plowman | CANGA312-17 | Vasconcelos L.V. 1066 | Yes | No | Yes | rbcL, ITS2 |
| Malpighiales | Erythroxylaceae | *Erythroxylum nelson-rosae* Plowman | CANGA313-17 | Vasconcelos L.V. 1071 | Yes | No | Yes | rbcL, ITS2 |
| Malpighiales | Erythroxylaceae | *Erythroxylum nelson-rosae* Plowman | CANGA314-17 | Vasconcelos L.V. 1093 | Yes | No | Yes | rbcL, ITS2 |
| Malpighiales | Erythroxylaceae | *Erythroxylum rufum* Cav. | CANGI197-17 | Vasconcelos L.V. 1035 | Yes | No | No | rbcL, ITS2 |
| Malpighiales | Erythroxylaceae | *Erythroxylum squamatum* Sw. | CANGA409-17 | Harley R.M. 57253 | Yes | Yes | Yes | rbcL, ITS2, rpoB, rpoC1 |
| Malpighiales | Erythroxylaceae | *Erythroxylum squamatum* Sw. | CANGA410-17 | Vasconcelos L.V. 760 | Yes | Yes | Yes | rbcL, ITS2 |
| Malpighiales | Euphorbiaceae | *Alchornea discolor* Poepp. | CANGA157-17 | Harley R.M. 52327 | Yes | Yes | Yes | rbcL, ITS2, trnH-psbA |
| Malpighiales | Euphorbiaceae | *Alchornea discolor* Poepp. | CANGA158-17 | Costa J.L.C. 8 | Yes | Yes | Yes | rbcL, ITS2, psbK-psbI |
| Malpighiales | Euphorbiaceae | *Alchornea discolor* Poepp. | CANGI205-17 | Costa J.L.C. 31 | Yes | Yes | Yes | matK, rpoB, rpoC1, psbK-psbI |
| Malpighiales | Euphorbiaceae | *Alchornea discolor* Poepp. | ITVRT019-17 | Vasconcelos L.V. 931 | Yes | No | Yes | rbcL, ITS2 |
| Malpighiales | Euphorbiaceae | *Aparisthmium cordatum* (A.Juss.) Baill. | ITVRT002-17 | Costa J.L.C. 1 | Yes | Yes | No | rbcL, ITS2 |
| Malpighiales | Euphorbiaceae | *Aparisthmium cordatum* (A.Juss.) Baill. | CANGI221-17 | Harley R.M. 58069 | Yes | No | No | rbcL, ITS2 |
| Malpighiales | Euphorbiaceae | *Astraea lobata* (L.) Klotzsch | CANGI199-17 | Costa J.L.C. 16 | Yes | Yes | No | rbcL, psbK-psbI |
| Malpighiales | Euphorbiaceae | *Astraea lobata* (L.) Klotzsch | CANGI200-17 | Costa J.L.C. 16 | Yes | Yes | No | rbcL |
| Malpighiales | Euphorbiaceae | *Croton* aff. *subferrugineus* Müll.Arg. | CANGI207-17 | Costa J.L.C. 24 | Yes | Yes | Yes | rbcL |
| Malpighiales | Euphorbiaceae | *Croton* sp. | CANGI201-17 | Harley R.M. 57331 | Yes | Yes | Yes | rbcL |
| Malpighiales | Euphorbiaceae | *Croton* sp. | CANGI209-17 | Vasconcelos L.V. 1036 | Yes | No | Yes | rbcL, ITS2 |
| Malpighiales | Euphorbiaceae | *Croton urucurana* Baill. | CANGA430-17 | Harley R.M. 57916 | No | No | No | rbcL |
| Malpighiales | Euphorbiaceae | *Dalechampia tiliifolia* Lam. | CANGI210-17 | Vasconcelos L.V. 1124 | No | No | No | rbcL, ITS2 |
| Malpighiales | Euphorbiaceae | *Mabea angustifolia* Spruce ex Benth. | CANGI211-17 | Harley R.M. 57256 | Yes | Yes | Yes | rbcL, ITS2, matK, rpoB |
| Malpighiales | Euphorbiaceae | *Mabea angustifolia* Spruce ex Benth. | CANGI218-17 | Costa J.L.C. 17 | Yes | Yes | Yes | rbcL, ITS2, matK, psbK-psbI |
| Malpighiales | Euphorbiaceae | *Mabea angustifolia* Spruce ex Benth. | CANGI219-17 | Costa J.L.C. 17 | Yes | Yes | Yes | rbcL, ITS2, rpoC1 |
| Malpighiales | Euphorbiaceae | *Mabea angustifolia* Spruce ex Benth. | CANGI212-17 | Pastore M. 308 | Yes | Yes | Yes | ITS2 |
| Malpighiales | Euphorbiaceae | *Manihot caerulescens* Pohl | CANGI215-17 | Pastore M. 417 | No | Yes | Yes | rbcL, ITS2 |
| Malpighiales | Euphorbiaceae | *Manihot quinquepartita* Huber ex D.J.Rogers & Appan | CANGI216-17 | Harley R.M. 58059 | Yes | No | Yes | rbcL, ITS2 |
| Malpighiales | Euphorbiaceae | *Manihot tristis* subsp. *surumuensis* (Ule) D.J.Rogers & Appan | CANGI214-17 | Harley R.M. 57456 | Yes | Yes | Yes | rbcL, ITS2, trnH-psbA |
| Malpighiales | Euphorbiaceae | *Plukenetia brachybotrya* Müll.Arg. | CANGI213-17 | Harley R.M. 57418 | No | Yes | Yes | rbcL, ITS2, matK, rpoB, rpoC1, psbK-psbI |
| Malpighiales | Euphorbiaceae | *Plukenetia brachybotrya* Müll.Arg. | CANGI217-17 | Harley R.M. 57955 | No | Yes | Yes | rbcL, ITS2 |
| Malpighiales | Euphorbiaceae | *Sapium glandulosum* (L.) Morong | CANGA209-17 | Vasconcelos L.V. 1023 | No | No | No | rbcL |
| Malpighiales | Euphorbiaceae | *Sapium glandulosum* (L.) Morong | CANGA210-17 | Vasconcelos L.V. 1068 | No | No | No | rbcL |
| Malpighiales | Humiriaceae | *Humiria balsamifera* var. *balsamifera* (Aubl.) A.St.-Hil. | CANGI273-17 | Pastore M. 448 | No | Yes | No | rbcL, ITS2 |
| Malpighiales | Humiriaceae | *Sacoglottis matogrossensis* Malme | CANGA280-17 | Vasconcelos L.V. 913 | Yes | No | Yes | rbcL |
| Malpighiales | Hypericaceae | *Vismia bemerguii* M.E.Berg | ITVRT023-17 | Harley R.M. 58183 | Yes | No | Yes | rbcL, ITS2 |
| Malpighiales | Hypericaceae | *Vismia gracilis* Hieron. | ITVRT015-17 | Harley R.M. 57914 | Yes | No | Yes | rbcL, ITS2 |
| Malpighiales | Hypericaceae | *Vismia gracilis* Hieron. | CANGA473-20 | Giulietti A.M. 2683 | Yes | No | Yes | ITS2 |
| Malpighiales | Hypericaceae | *Vismia gracilis* Hieron. | CANGA476-20 | Giulietti A.M. 2614 | Yes | No | Yes | ITS2 |
| Malpighiales | Hypericaceae | *Vismia schultesii* N.Robson | CANGI274-17 | Costa J.L.C. 5 | Yes | Yes | Yes | ITS2 |
| Malpighiales | Hypericaceae | *Vismia schultesii* N.Robson | CGII656-20 | Zappi D.C. 4539 | Yes | No | Yes | ITS2 |
| Malpighiales | Lacistemataceae | *Lacistema aggregatum* (P.J.Bergius) Rusby | CANGI279-17 | Harley R.M. 57241 | Yes | Yes | No | rbcL, ITS2, matK, rpoB, rpoC1, atpF-atpH |
| Malpighiales | Malpighiaceae | *Banisteriopsis malifolia* var. *appressa* B.Gates | CANGI317-17 | Vasconcelos L.V. 776 | Yes | Yes | Yes | rbcL, ITS2 |
| Malpighiales | Malpighiaceae | *Banisteriopsis malifolia* var. *appressa* B.Gates | CANGI321-17 | Vasconcelos L.V. 1054 | Yes | No | Yes | rbcL |
| Malpighiales | Malpighiaceae | *Banisteriopsis muricata* (Cav.) Cuatrec. | CANGI323-17 | Pastore M. 505 | Yes | No | No | rbcL, ITS2 |
| Malpighiales | Malpighiaceae | *Banisteriopsis stellaris* (Griseb.) B.Gates | CANGI315-17 | Praia T.S. 1 | Yes | Yes | Yes | ITS2, matK, rpoB |
| Malpighiales | Malpighiaceae | *Banisteriopsis stellaris* (Griseb.) B.Gates | CANGI307-17 | Viana P.L. 6107 | Yes | Yes | Yes | rbcL, ITS2 |
| Malpighiales | Malpighiaceae | *Banisteriopsis stellaris* (Griseb.) B.Gates | CANGI322-17 | Vasconcelos L.V. 1151 | Yes | No | Yes | rbcL, ITS2 |
| Malpighiales | Malpighiaceae | *Banisteriopsis stellaris* (Griseb.) B.Gates | CANGI309-17 | Harley R.M. 58102 | Yes | No | Yes | rbcL, ITS2 |
| Malpighiales | Malpighiaceae | *Byrsonima chrysophylla* Kunth | CANGA045-17 | Dias C.S.P. 1 | Yes | Yes | Yes | rbcL, ITS2, matK, rpoB, rpoC1, atpF-atpH, psbK-psbI |
| Malpighiales | Malpighiaceae | *Byrsonima chrysophylla* Kunth | CANGI311-17 | Viana P.L. 6191 | Yes | Yes | Yes | rbcL, ITS2 |
| Malpighiales | Malpighiaceae | *Byrsonima chrysophylla* Kunth | CANGA462-20 | Harley R.M. 57864 | Yes | No | Yes | rbcL, ITS2 |
| Malpighiales | Malpighiaceae | *Byrsonima chrysophylla* Kunth | CANGA406-17 | Vasconcelos L.V. 1067 | Yes | No | Yes | rbcL, ITS2 |
| Malpighiales | Malpighiaceae | *Byrsonima chrysophylla* Kunth | CANGI312-17 | Vasconcelos L.V. 1136 | Yes | No | Yes | rbcL, ITS2 |
| Malpighiales | Malpighiaceae | *Byrsonima chrysophylla* Kunth | CANGI313-17 | Harley R.M. 58085 | Yes | No | Yes | rbcL, ITS2 |
| Malpighiales | Malpighiaceae | *Byrsonima stipulacea* A.Juss. | CANGI310-17 | Harley R.M. 57420 | Yes | Yes | Yes | ITS2, matK, trnH-psbA |
| Malpighiales | Malpighiaceae | *Diplopterys pubipetala* (A.Juss.) W.R.Anderson & C.C.Davis | CANGI308-17 | Harley R.M. 57998 | Yes | Yes | No | rbcL, ITS2 |
| Malpighiales | Malpighiaceae | *Diplopterys pubipetala* (A.Juss.) W.R.Anderson & C.C.Davis | CANGI318-17 | Harley R.M. 57969 | Yes | Yes | No | rbcL, ITS2 |
| Malpighiales | Malpighiaceae | *Diplopterys pubipetala* (A.Juss.) W.R.Anderson & C.C.Davis | CANGI319-17 | Vasconcelos L.V. 1032 | Yes | No | No | rbcL, ITS2 |
| Malpighiales | Malpighiaceae | *Heteropterys nervosa* A.Juss. | CANGI316-17 | Dias C.S.P. 5 | Yes | Yes | Yes | rbcL, matK, atpF-atpH, psbK-psbI |
| Malpighiales | Malpighiaceae | *Heteropterys nervosa* A.Juss. | CANGI320-17 | Vasconcelos L.V. 1033 | Yes | No | Yes | rbcL, ITS2 |
| Malpighiales | Malpighiaceae | *Niedenzuella acutifolia* (Cav.) W.R.Anderson | CANGI314-17 | Reis A.S. 29 | Yes | Yes | No | rbcL, ITS2, psbK-psbI |
| Malpighiales | Ochnaceae | *Ouratea castaneifolia* (DC.) Engl. | CANGI379-17 | Viana P.L. 5782 | Yes | Yes | Yes | rbcL, ITS2, rpoB |
| Malpighiales | Ochnaceae | *Ouratea castaneifolia* (DC.) Engl. | CANGI385-17 | Vasconcelos L.V. 1043 | Yes | No | Yes | rbcL, ITS2 |
| Malpighiales | Ochnaceae | *Ouratea racemiformis* Ule | CANGI378-17 | Gil A. 521 | Yes | Yes | Yes | rbcL |
| Malpighiales | Ochnaceae | *Sauvagesia longifolia* Eichler | CGII650-20 | Zappi D.C. 4558 | No | No | Yes | rbcL |
| Malpighiales | Ochnaceae | *Sauvagesia tenella* Lam. | CANGI388-17 | Pastore M. 387 | Yes | Yes | Yes | rbcL, ITS2, trnH-psbA |
| Malpighiales | Ochnaceae | *Sauvagesia tenella* Lam. | CANGI390-17 | Vasconcelos L.V. 1090 | Yes | No | Yes | rbcL, ITS2 |
| Malpighiales | Ochnaceae | *Sauvagesia tenella* Lam. | CANGI389-17 | Pastore M. 590 | Yes | No | Yes | rbcL, ITS2 |
| Malpighiales | Passifloraceae | *Passiflora araujoi* Sacco | CANGA022-17 | Vasconcelos L.V. 905 | No | No | Yes | rbcL |
| Malpighiales | Passifloraceae | *Passiflora auriculata* Kunth | CANGI417-17 | Harley R.M. 57240 | No | Yes | No | rbcL, ITS2, matK, rpoB, psbK-psbI |
| Malpighiales | Passifloraceae | *Passiflora glandulosa* Cav. | CANGA131-17 | Harley R.M. 57238 | Yes | Yes | Yes | rbcL, ITS2, matK |
| Malpighiales | Passifloraceae | *Passiflora glandulosa* Cav. | CANGA207-17 | Harley R.M. 57317 | Yes | Yes | Yes | rbcL, ITS2, matK, rpoB |
| Malpighiales | Passifloraceae | *Passiflora glandulosa* Cav. | CANGA208-17 | Harley R.M. 57326 | Yes | Yes | Yes | rbcL, matK, rpoB, rpoC1, atpF-atpH |
| Malpighiales | Passifloraceae | *Passiflora longifilamentosa* A.K.Koch, A.Cardoso & Ilk.-Borg. | CANGA447-20 | Mota N.F.O. 3446 | No | Yes | Yes | rbcL, ITS2, matK, rpoB |
| Malpighiales | Passifloraceae | *Passiflora* sp. | CANGI416-17 | Harley R.M. 57239 | Yes | Yes | Yes | rbcL, ITS2, matK |
| Malpighiales | Passifloraceae | *Passiflora tholozanii* Sacco | CANGA423-17 | Gil A. 503 | Yes | Yes | Yes | rbcL, ITS2 |
| Malpighiales | Passifloraceae | *Passiflora vespertilio* L. | CANGI418-17 | Lopes C.S.A. 11 | No | Yes | No | rbcL, ITS2, rpoC1 |
| Malpighiales | Phyllanthaceae | *Phyllanthus hyssopifolioides* Kunth | CANGA235-17 | Santos F. E1TZ03MAR | Yes | Yes | Yes | rbcL, rpoB, rpoC1, atpF-atpH, psbK-psbI |
| Malpighiales | Phyllanthaceae | *Phyllanthus hyssopifolioides* Kunth | CANGA236-17 | Santos F. E3BOC04MAR | Yes | Yes | Yes | rbcL, ITS2, rpoC1, psbK-psbI, trnH-psbA |
| Malpighiales | Phyllanthaceae | *Phyllanthus minutulus* Müll.Arg. | CANGI436-17 | Harley R.M. 58113 | Yes | No | Yes | rbcL, ITS2 |
| Malpighiales | Phyllanthaceae | *Phyllanthus myrsinites* Kunth | CANGI435-17 | Pastore M. 467 | No | Yes | Yes | rbcL, ITS2 |
| Malpighiales | Salicaceae | *Banara guianensis* Aubl. | CANGA480-20 | Nogueira M.G.C. 667 | No | No | No | ITS2 |
| Malpighiales | Salicaceae | *Casearia javitensis* Kunth | CANGA243-17 | Mota N.F.O. 3389 | Yes | Yes | No | rbcL, ITS2, rpoB, atpF-atpH, psbK-psbI |
| Malpighiales | Salicaceae | *Casearia javitensis* Kunth | CANGA244-17 | Harley R.M. 57463 | Yes | Yes | No | ITS2 |
| Malpighiales | Salicaceae | *Casearia javitensis* Kunth | CANGA464-20 | Harley R.M. 57867 | Yes | No | No | rbcL, ITS2 |
| Malpighiales | Salicaceae | *Casearia javitensis* Kunth | CANGA245-17 | Vasconcelos L.V. 920 | Yes | No | No | ITS2 |
| Malpighiales | Salicaceae | *Ryania speciosa* Vahl | CANGI017-17 | Harley R.M. 57416 | Yes | Yes | No | rbcL, ITS2, rpoB, rpoC1, psbK-psbI, trnH-psbA |
| Malpighiales | Turneraceae | *Turnera brasiliensis* Willd. ex Schult. | CANGA477-20 | Giulietti A.M. 2615 | No | No | Yes | rbcL, ITS2 |
| Malpighiales | Turneraceae | *Turnera coerulea* DC. | CANGI427-17 | Viana P.L. 6182 | Yes | Yes | No | rbcL, ITS2 |
| Malpighiales | Turneraceae | *Turnera glaziovii* Urb. | CANGI420-17 | Meirelles J. 928 | Yes | Yes | Yes | rbcL, rpoB, rpoC1 |
| Malpighiales | Turneraceae | *Turnera glaziovii* Urb. | CANGA211-17 | Trindade J.R. 348 | Yes | Yes | Yes | ITS2, rpoB |
| Malpighiales | Turneraceae | *Turnera glaziovii* Urb. | CANGI432-17 | Reis A.S. 33 | Yes | Yes | Yes | rbcL, ITS2, rpoB, rpoC1 |
| Malpighiales | Turneraceae | *Turnera glaziovii* Urb. | CANGI424-17 | Dias C.S.P. 13 | Yes | Yes | Yes | rbcL, rpoB, rpoC1 |
| Malpighiales | Turneraceae | *Turnera glaziovii* Urb. | CANGI561-17 | Pastore M. 362 | Yes | Yes | Yes | rbcL, ITS2, trnH-psbA |
| Malpighiales | Turneraceae | *Turnera melochioides* Cambess. | CANGI429-17 | Vasconcelos L.V. 1130 | Yes | No | No | rbcL, ITS2 |
| Malpighiales | Turneraceae | *Turnera melochioides* Cambess. | CANGI434-17 | Harley R.M. 58137 | Yes | No | No | rbcL |
| Malpighiales | Turneraceae | *Turnera melochioides* var. *arenaria* Cambess. | CANGI433-17 | Viana P.L. 6235 | Yes | Yes | No | rbcL |
| Malpighiales | Turneraceae | *Turnera melochioides* var. *arenaria* Cambess. | CANGI428-17 | Harley R.M. 57967 | Yes | Yes | No | rbcL, ITS2 |
| Malpighiales | Turneraceae | *Turnera melochioides* var. *latifolia* Cambess. | CANGI431-17 | Costa J.L.C. 3 | Yes | Yes | No | rbcL, ITS2, rpoB |
| Malpighiales | Turneraceae | *Turnera melochioides* var. *latifolia* Cambess. | CANGI425-17 | Praia T.S. 4 | Yes | Yes | No | rbcL, ITS2, matK, rpoB, rpoC1, psbK-psbI |
| Malpighiales | Turneraceae | *Turnera melochioides* var. *latifolia* Cambess. | CANGI421-17 | Pastore M. 325 | Yes | Yes | No | rbcL, ITS2 |
| Malpighiales | Turneraceae | *Turnera melochioides* var. *latifolia* Cambess. | CANGI426-17 | Viana P.L. 6160 | Yes | Yes | No | rbcL, ITS2 |
| Malpighiales | Turneraceae | *Turnera melochioides* var. *latifolia* Cambess. | CANGI422-17 | Harley R.M. 57512 | Yes | Yes | No | rbcL, ITS2 |
| Malpighiales | Violaceae | *Rinorea pubiflora* (Benth.) Sprague & Sandwith | CANGI612-17 | Harley R.M. 57318 | No | Yes | No | rbcL, matK, rpoB, atpF-atpH, psbK-psbI |
| Malpighiales | Violaceae | *Rinoreocarpus ulei* (Melch.) Ducke | CANGI554-17 | Harley R.M. 57951 | No | Yes | No | rbcL, ITS2 |
| Malvales | Malvaceae | *Apeiba tibourbou* Aubl. | CANGI324-17 | Harley R.M. 58052 | No | No | No | rbcL, ITS2 |
| Malvales | Malvaceae | *Melochia arenosa* Benth. | CANGI352-17 | Harley R.M. 57349 | Yes | Yes | Yes | rbcL, ITS2, matK, rpoC1 |
| Malvales | Malvaceae | *Melochia arenosa* Benth. | CANGI332-17 | Viana P.L. 6162 | Yes | Yes | Yes | rbcL, ITS2 |
| Malvales | Malvaceae | *Melochia arenosa* Benth. | CANGI328-17 | Harley R.M. 57982 | Yes | Yes | Yes | rbcL, ITS2 |
| Malvales | Malvaceae | *Melochia splendens* A.St.-Hil. & Naudin | CANGI329-17 | Pastore M. 584 | Yes | No | Yes | rbcL, ITS2 |
| Malvales | Malvaceae | *Peltaea riedelii* (Gürke) Standl. | CANGI334-17 | Nogueira M.G.C. 678 | No | No | Yes | rbcL, ITS2 |
| Malvales | Malvaceae | *Pseudobombax longiflorum* (Mart.) A.Robyns | CANGI330-17 | Harley R.M. 57925 | Yes | No | No | rbcL |
| Malvales | Malvaceae | *Urena lobata* L. | CANGI333-17 | Pastore M. 393 | Yes | Yes | No | rbcL, ITS2, trnH-psbA |
| Malvales | Thymelaeaceae | *Daphnopsis filipedunculata* Nevling & Barringer | CANGA192-17 | Harley R.M. 57246 | Yes | Yes | Yes | rbcL, ITS2, matK, rpoC1, atpF-atpH |
| Malvales | Thymelaeaceae | *Daphnopsis filipedunculata* Nevling & Barringer | CANGA195-17 | Vasconcelos L.V. 933 | Yes | No | Yes | rbcL, ITS2 |
| Myrtales | Combretaceae | *Combretum duarteanum* Cambess. | CANGI108-17 | Nogueira M.G.C. 657 | No | No | Yes | rbcL |
| Myrtales | Combretaceae | *Combretum rotundifolium* Rich. | CANGI107-17 | Harley R.M. 57930 | Yes | No | Yes | rbcL |
| Myrtales | Lythraceae | *Cuphea annulata* Koehne | CANGA017-17 | Harley R.M. 57310 | Yes | Yes | Yes | rbcL, ITS2, matK, rpoB, rpoC1, atpF-atpH |
| Myrtales | Lythraceae | *Cuphea annulata* Koehne | CANGA018-17 | Viana P.L. 6105 | Yes | Yes | Yes | ITS2 |
| Myrtales | Lythraceae | *Cuphea carajasensis* Lourteig | CANGA064-17 | Viana P.L. 5568 | Yes | Yes | Yes | rbcL, ITS2, matK, rpoC1 |
| Myrtales | Lythraceae | *Cuphea carajasensis* Lourteig | CANGA067-17 | Harley R.M. 57468 | Yes | Yes | Yes | rbcL, ITS2 |
| Myrtales | Lythraceae | *Cuphea carajasensis* Lourteig | CANGA068-17 | Harley R.M. 57470 | Yes | Yes | Yes | ITS2 |
| Myrtales | Lythraceae | *Cuphea carajasensis* Lourteig | CANGA069-17 | Vasconcelos L.V. 859 | Yes | Yes | Yes | ITS2 |
| Myrtales | Lythraceae | *Cuphea carajasensis* Lourteig | CANGA070-17 | Vasconcelos L.V. 846 | Yes | Yes | Yes | ITS2 |
| Myrtales | Lythraceae | *Cuphea carajasensis* Lourteig | CANGA071-17 | Viana P.L. 6116 | Yes | Yes | Yes | ITS2 |
| Myrtales | Lythraceae | *Cuphea carajasensis* Lourteig | CANGA072-17 | Viana P.L. 6231 | Yes | Yes | Yes | rbcL, ITS2 |
| Myrtales | Lythraceae | *Cuphea carajasensis* Lourteig | CANGA074-17 | Harley R.M. 57497 | Yes | Yes | Yes | rbcL, ITS2 |
| Myrtales | Lythraceae | *Cuphea carajasensis* Lourteig | CANGA075-17 | Harley R.M. 57511 | Yes | Yes | Yes | rbcL, ITS2 |
| Myrtales | Lythraceae | *Cuphea carajasensis* Lourteig | CANGA076-17 | Harley R.M. 57507 | Yes | Yes | Yes | rbcL, ITS2 |
| Myrtales | Lythraceae | *Cuphea carajasensis* Lourteig | CANGA077-17 | Vasconcelos L.V. 1089 | Yes | No | Yes | rbcL, ITS2 |
| Myrtales | Lythraceae | *Cuphea carajasensis* Lourteig | CANGA078-17 | Vasconcelos L.V. 1133 | Yes | No | Yes | ITS2 |
| Myrtales | Lythraceae | *Cuphea carajasensis* Lourteig | CANGI306-17 | Harley R.M. 58111 | Yes | No | Yes | rbcL, ITS2 |
| Myrtales | Lythraceae | *Cuphea tenuissima* Koehne | CGII620-20 | Zappi D.C. 4318 | No | No | Yes | rbcL, ITS2 |
| Myrtales | Melastomataceae | *Bellucia grossularioides* (L.) Triana | CANGI347-17 | Nogueira M.G.C. 691 | No | No | No | rbcL, ITS2 |
| Myrtales | Melastomataceae | *Miconia heliotropoides* Triana | CANGI350-17 | Gil A. 507 | Yes | Yes | Yes | ITS2 |
| Myrtales | Melastomataceae | *Miconia heliotropoides* Triana | CANGA448-20 | Meirelles J. 941 | Yes | Yes | Yes | ITS2 |
| Myrtales | Melastomataceae | *Noterophila crassipes* (Naudin.) Kriebel & M.J.R.Rocha | CANGA151-17 | Harley R.M. 57270 | Yes | Yes | Yes | ITS2, rpoB |
| Myrtales | Melastomataceae | *Tibouchina* sp. | CANGI351-17 | Vasconcelos L.V. 1105 | Yes | No | Yes | ITS2 |
| Myrtales | Myrtaceae | *Eugenia anastomosans* DC. | CANGA015-17 | Vasconcelos L.V. 922 | Yes | No | Yes | rbcL, ITS2 |
| Myrtales | Myrtaceae | *Eugenia flavescens* DC. | CANGA200-17 | Vasconcelos L.V. 921 | Yes | No | Yes | rbcL, ITS2 |
| Myrtales | Myrtaceae | *Eugenia flavescens* DC. | CANGI363-17 | Vasconcelos L.V. 1100 | Yes | No | Yes | rbcL, ITS2 |
| Myrtales | Myrtaceae | *Eugenia punicifolia* (Kunth) DC. | CANGA364-17 | Viana P.L. 6111 | Yes | Yes | No | rbcL, ITS2 |
| Myrtales | Myrtaceae | *Eugenia punicifolia* (Kunth) DC. | CANGA365-17 | Viana P.L. 6209 | Yes | Yes | No | rbcL, ITS2 |
| Myrtales | Myrtaceae | *Eugenia punicifolia* (Kunth) DC. | CANGI361-17 | Vasconcelos L.V. 1094 | Yes | No | No | rbcL, ITS2 |
| Myrtales | Myrtaceae | *Eugenia punicifolia* (Kunth) DC. | CANGI364-17 | Harley R.M. 58134 | Yes | No | No | rbcL, ITS2 |
| Myrtales | Myrtaceae | *Myrcia bracteata* (Rich.) DC. | ITVRT013-17 | Pastore M. 397 | Yes | Yes | Yes | rbcL, ITS2, trnH-psbA |
| Myrtales | Myrtaceae | *Myrcia grandis* McVaugh | CANGA155-17 | Trindade J.R. 253 | Yes | Yes | Yes | ITS2, matK, rpoB, atpF-atpH |
| Myrtales | Myrtaceae | *Myrcia guianensis* (Aubl.) DC. | ITVRT025-17 | Vasconcelos L.V. 1041 | Yes | No | No | rbcL, ITS2 |
| Myrtales | Myrtaceae | *Myrcia multiflora* (Lam.) DC. | CANGA300-17 | Gil A. 508 | Yes | Yes | No | ITS2 |
| Myrtales | Myrtaceae | *Myrcia multiflora* (Lam.) DC. | CANGA301-17 | Vasconcelos L.V. 1042 | Yes | No | No | ITS2 |
| Myrtales | Myrtaceae | *Myrcia sylvatica* (G.Mey.) DC. | CANGI360-17 | Praia T.S. 16 | Yes | Yes | Yes | ITS2 |
| Myrtales | Myrtaceae | *Myrcia sylvatica* (G.Mey.) DC. | ITVRT012-17 | Viana P.L. 6219 | Yes | Yes | Yes | rbcL, ITS2 |
| Myrtales | Myrtaceae | *Myrcia sylvatica* (G.Mey.) DC. | ITVRT026-17 | Vasconcelos L.V. 1058 | Yes | No | Yes | rbcL, ITS2 |
| Myrtales | Myrtaceae | *Myrcia tenuiflora* A.R.Lourenço & E.Lucas | CANGA034-17 | Dias C.S.P. 4 | Yes | Yes | Yes | rpoC1 |
| Myrtales | Onagraceae | *Ludwigia nervosa* (Poir.) H.Hara | CANGI396-17 | Pastore M. 559 | Yes | No | No | rbcL, ITS2 |
| Myrtales | Onagraceae | *Ludwigia octovalvis* (Jacq.) P.H.Raven | CANGI394-17 | Harley R.M. 57876 | Yes | No | No | rbcL, ITS2 |
| Myrtales | Vochysiaceae | *Vochysia haenkeana* Mart. | CANGA224-17 | Pastore M. 380 | Yes | Yes | No | rbcL |
| Nymphaeales | Cabombaceae | *Cabomba haynesii* Wiersema | CANGI093-17 | Viana P.L. 6178 | Yes | Yes | Yes | rbcL, ITS2 |
| Nymphaeales | Nymphaeaceae | *Nymphaea conardii* Wiersema | CANGI377-17 | Pastore M. 554 | No | No | Yes | rbcL, ITS2 |
| Oxalidales | Connaraceae | *Rourea doniana* Baker | CANGA457-20 | Harley R.M. 57858 | Yes | No | Yes | rbcL, ITS2 |
| Oxalidales | Connaraceae | *Rourea doniana* Baker | CANGA164-17 | Vasconcelos L.V. 1148 | Yes | No | Yes | rbcL, ITS2 |
| Oxalidales | Connaraceae | *Rourea ligulata* Baker | CANGA258-17 | Viana P.L. 6186 | Yes | Yes | Yes | rbcL, ITS2 |
| Pandanales | Cyclanthaceae | *Asplundia latifrons* (Drude) Harling | CANGI132-17 | Vasconcelos L.V. 1076 | No | No | Yes | rbcL, ITS2 |
| Pandanales | Velloziaceae | *Vellozia glauca* Pohl | CANGI391-17 | Harley R.M. 57428 | Yes | Yes | No | rbcL, ITS2, matK, rpoC1, psbK-psbI, trnH-psbA |
| Pandanales | Velloziaceae | *Vellozia glauca* Pohl | CANGA213-17 | Viana P.L. 6202 | Yes | Yes | No | rbcL, ITS2 |
| Pandanales | Velloziaceae | *Vellozia glauca* Pohl | CANGI602-17 | Vasconcelos L.V. 1107 | Yes | No | No | rbcL, ITS2 |
| Pandanales | Velloziaceae | *Vellozia glochidea* Pohl | CANGA214-17 | Vasconcelos L.V. 1095 | No | No | Yes | rbcL, ITS2 |
| Pandanales | Velloziaceae | *Vellozia graminea* Pohl | ITVGA003-17 | Nogueira M.G.C. 414 | No | Yes | Yes | rbcL, matK, rpoB, rpoC1, atpF-atpH, psbK-psbI |
| Picramniales | Picramniaceae | *Picramnia ferrea* Pirani & W.W.Thomas | CANGA191-17 | Harley R.M. 57278 | Yes | Yes | Yes | rpoB, atpF-atpH |
| Piperales | Aristolochiaceae | *Aristolochia disticha* Mast. | CANGI600-17 | Pastore M. 513 | Yes | No | Yes | rbcL, ITS2 |
| Piperales | Aristolochiaceae | *Aristolochia rugosa* Lam. | CANGI045-17 | Trindade J.R. 380 | Yes | Yes | Yes | rbcL, ITS2, rpoB |
| Piperales | Aristolochiaceae | *Aristolochia* sp. | CANGI044-17 | Viana P.L. 5667 | No | Yes | Yes | rbcL, ITS2, matK, rpoB, rpoC1, atpF-atpH, trnH-psbA |
| Piperales | Aristolochiaceae | *Aristolochia stomachoidis* Hoehne | CANGI047-17 | Nogueira M.G.C. 689 | No | No | Yes | rbcL |
| Piperales | Piperaceae | *Peperomia albopilosa* D.Monteiro | CANGI438-17 | Viana P.L. 6169 | Yes | Yes | Yes | rbcL, ITS2 |
| Piperales | Piperaceae | *Peperomia macrostachya* (Vahl) A.Dietr. | CANGI035-17 | Mota N.F.O. 3440 | Yes | Yes | No | rbcL, ITS2, rpoB, psbK-psbI, trnH-psbA |
| Piperales | Piperaceae | *Peperomia obtusifolia* (L.) A.Dietr. | CANGI437-17 | Dias C.S.P. 10 | Yes | Yes | Yes | rbcL, rpoC1, psbK-psbI |
| Piperales | Piperaceae | *Peperomia pellucida* (L.) Kunth | CANGI441-17 | Pastore M. 536 | No | No | No | rbcL, ITS2 |
| Piperales | Piperaceae | *Peperomia sulcata* C.DC. | CANGI442-17 | Pastore M. 540 | No | No | Yes | rbcL, ITS2 |
| Piperales | Piperaceae | *Piper arboreum* Aubl. | CANGI440-17 | Harley R.M. 57941 | Yes | No | No | rbcL |
| Poales | Bromeliaceae | *Aechmea mertensii* (G.Mey.) Schult. & Schult.f. | CANGA286-17 | Pastore M. 381 | Yes | Yes | No | rbcL, trnH-psbA |
| Poales | Bromeliaceae | *Dyckia duckei* L.B.Sm. | CANGA465-20 | Harley R.M. 57869 | Yes | No | Yes | rbcL |
| Poales | Bromeliaceae | *Dyckia duckei* L.B.Sm. | CANGA165-17 | Harley R.M. 58118 | Yes | No | Yes | rbcL |
| Poales | Bromeliaceae | *Pitcairnia lanuginosa* Ruiz & Pav. | CANGI089-17 | Reis A.S. 42 | No | Yes | Yes | rbcL, matK, rpoB, rpoC1, atpF-atpH, psbK-psbI |
| Poales | Bromeliaceae | *Tillandsia adpressiflora* Mez | CANGI088-17 | Viana P.L. 6109 | No | Yes | Yes | rbcL |
| Poales | Bromeliaceae | *Tillandsia streptocarpa* Baker | CANGA411-17 | Cardoso A. 1998 | Yes | Yes | Yes | rbcL, ITS2, matK |
| Poales | Bromeliaceae | *Tillandsia streptocarpa* Baker | CANGI087-17 | Dias C.S.P. 17 | Yes | Yes | Yes | matK |
| Poales | Bromeliaceae | *Tillandsia streptocarpa* Baker | CANGA412-17 | Harley R.M. 57900 | Yes | Yes | Yes | rbcL |
| Poales | Cyperaceae | *Bulbostylis cangae* C.S. Nunes & A. Gil | CANGI133-17 | Harley R.M. 57325 | Yes | Yes | Yes | rbcL, ITS2 |
| Poales | Cyperaceae | *Bulbostylis capillaris* (L.) C.B.Clarke | CANGI134-17 | Viana P.L. 6185 | No | Yes | No | rbcL, ITS2 |
| Poales | Cyperaceae | *Bulbostylis conifera* (Kunth) C.B.Clarke | CANGA058-17 | Falcão B.F. 210 | Yes | Yes | Yes | rbcL, ITS2, rpoB, rpoC1, atpF-atpH |
| Poales | Cyperaceae | *Bulbostylis conifera* (Kunth) C.B.Clarke | CANGA059-17 | Santos F. E2FLO.08 | Yes | Yes | Yes | rbcL, ITS2, rpoB, rpoC1, atpF-atpH |
| Poales | Cyperaceae | *Bulbostylis conifera* (Kunth) C.B.Clarke | CANGA060-17 | Santos F. E1TZ01MAR | Yes | Yes | Yes | rbcL, ITS2 |
| Poales | Cyperaceae | *Bulbostylis conifera* (Kunth) C.B.Clarke | CANGA149-17 | Praia T.S. 27 | Yes | Yes | Yes | rbcL, ITS2, atpF-atpH |
| Poales | Cyperaceae | *Bulbostylis conifera* (Kunth) C.B.Clarke | CANGA040-17 | Vasconcelos L.V. 813 | Yes | Yes | Yes | rbcL, ITS2 |
| Poales | Cyperaceae | *Bulbostylis conifera* (Kunth) C.B.Clarke | CANGA150-17 | Viana P.L. 6147 | Yes | Yes | Yes | rbcL, ITS2 |
| Poales | Cyperaceae | *Bulbostylis conifera* (Kunth) C.B.Clarke | CANGI135-17 | Pastore M. 650 | Yes | No | Yes | rbcL, ITS2 |
| Poales | Cyperaceae | *Bulbostylis conifera* (Kunth) C.B.Clarke | CANGI136-17 | Harley R.M. 58088 | Yes | No | Yes | rbcL, ITS2 |
| Poales | Cyperaceae | *Bulbostylis junciformis* (Kunth) C.B.Clarke | CGII608-20 | Zappi D.C. 4278 | No | No | Yes | rbcL, ITS2 |
| Poales | Cyperaceae | *Bulbostylis junciformis* (Kunth) C.B.Clarke | CGII626-20 | Zappi D.C. 4341 | No | No | Yes | rbcL |
| Poales | Cyperaceae | *Calyptrocarya poeppigiana* Kunth | CANGA252-17 | Harley R.M. 57934 | No | No | Yes | rbcL |
| Poales | Cyperaceae | *Cyperus aggregatus* (Willd.) Endl. | CANGI162-17 | Harley R.M. 58101 | Yes | No | Yes | rbcL, ITS2 |
| Poales | Cyperaceae | *Cyperus haspan* L. | CANGA225-17 | Harley R.M. 57889 | Yes | No | No | rbcL |
| Poales | Cyperaceae | *Cyperus luzulae* (L.) Retz. | CANGA267-17 | Dias C.S.P. 15 | Yes | Yes | No | rbcL, ITS2, rpoC1 |
| Poales | Cyperaceae | *Cyperus sesquiflorus* (Torr.) Mattf. & Kük. | CANGI158-17 | Reis A.S. 24 | Yes | Yes | Yes | rbcL |
| Poales | Cyperaceae | *Cyperus sesquiflorus* (Torr.) Mattf. & Kük. | CANGA322-17 | Dias C.S.P. 16 | Yes | Yes | Yes | rbcL, matK, rpoB, rpoC1, atpF-atpH, trnH-psbA |
| Poales | Cyperaceae | *Cyperus simplex* Kunth | CANGI160-17 | Pastore M. 538 | No | No | Yes | ITS2 |
| Poales | Cyperaceae | *Eleocharis ayacuchensis* S. González & Reznicek | CANGI142-17 | Harley R.M. 58115 | Yes | No | Yes | rbcL, ITS2 |
| Poales | Cyperaceae | *Eleocharis flavescens* (Poir.) Urb. | CANGI137-17 | Viana P.L. 5682 | Yes | Yes | No | rbcL, ITS2, rpoC1, atpF-atpH, psbK-psbI |
| Poales | Cyperaceae | *Eleocharis flavescens* (Poir.) Urb. | CANGI141-17 | Pastore M. 627 | Yes | No | No | rbcL, ITS2 |
| Poales | Cyperaceae | *Eleocharis geniculata* (L.) Roem. & Schult. | CANGA206-17 | Santos F. E1S11B02MAR | Yes | Yes | No | rbcL, rpoC1, atpF-atpH, psbK-psbI |
| Poales | Cyperaceae | *Eleocharis minima* Kunth | CANGA295-17 | Santos F. E1TZ03MAR | No | Yes | No | rbcL, ITS2, rpoC1, atpF-atpH |
| Poales | Cyperaceae | *Eleocharis pedrovianae* C.S. Nunes, R. Trevis. & A. Gil | CANGI138-17 | Mota N.F.O. 3414 | Yes | Yes | Yes | rbcL, atpF-atpH, psbK-psbI |
| Poales | Cyperaceae | *Eleocharis pedrovianae* C.S. Nunes, R. Trevis. & A. Gil | CANGA339-17 | Viana P.L. 6174 | Yes | Yes | Yes | rbcL, ITS2 |
| Poales | Cyperaceae | *Fimbristylis dichotoma* (L.) Vahl | CANGI161-17 | Nogueira M.G.C. 695 | Yes | No | No | rbcL, ITS2 |
| Poales | Cyperaceae | *Hypolytrum paraense* M.Alves & W.W.Thomas | CANGA329-17 | Mota N.F.O. 3384 | Yes | Yes | Yes | rbcL, ITS2, matK, rpoC1 |
| Poales | Cyperaceae | *Hypolytrum paraense* M.Alves & W.W.Thomas | CANGI143-17 | Gil A. 502 | Yes | Yes | Yes | rbcL, ITS2, rpoC1 |
| Poales | Cyperaceae | *Rhynchospora acanthoma* A.C.Araújo & Longhi-Wagner | CANGI151-17 | Harley R.M. 57499 | Yes | Yes | Yes | rbcL, ITS2 |
| Poales | Cyperaceae | *Rhynchospora barbata* (Vahl) Kunth | CANGA031-17 | Vasconcelos L.V. 1096 | Yes | No | Yes | rbcL, ITS2 |
| Poales | Cyperaceae | *Rhynchospora candida* (Nees) Boeckeler | CANGA050-17 | Trindade J.R. 227 | Yes | Yes | Yes | rbcL, ITS2, rpoB, rpoC1, psbK-psbI |
| Poales | Cyperaceae | *Rhynchospora candida* (Nees) Boeckeler | CANGA051-17 | Gil A. 524 | Yes | Yes | Yes | rbcL, rpoC1, psbK-psbI |
| Poales | Cyperaceae | *Rhynchospora cephalotes* (L.) Vahl | CANGA440-17 | s.n. | No | Yes | Yes | rbcL, matK |
| Poales | Cyperaceae | *Rhynchospora cephalotes* (L.) Vahl | CGII627-20 | Zappi D.C. 4342 | No | No | Yes | rbcL |
| Poales | Cyperaceae | *Rhynchospora comata* (Link) Roem. & Schult. | CANGA446-20 | Mota N.F.O. 3448 | No | Yes | Yes | rbcL, ITS2, matK, rpoB, psbK-psbI, trnH-psbA |
| Poales | Cyperaceae | *Rhynchospora eximia* (Nees) Boeckeler | CANGA120-17 | Santos F. E2FLO.13 | Yes | Yes | No | rbcL, ITS2 |
| Poales | Cyperaceae | *Rhynchospora eximia* (Nees) Boeckeler | CANGA052-17 | Falcão B.F. 256 | Yes | Yes | No | rbcL, ITS2, rpoC1, atpF-atpH, psbK-psbI |
| Poales | Cyperaceae | *Rhynchospora filiformis* Vahl | CANGI148-17 | Viana P.L. 6159 | Yes | Yes | Yes | rbcL, ITS2 |
| Poales | Cyperaceae | *Rhynchospora* sp. | CANGI150-17 | Pastore M. 643 | No | No | Yes | rbcL, ITS2 |
| Poales | Cyperaceae | *Rhynchospora trichochaeta* C.B.Clarke | CANGA424-17 | Santos F. E1TZ01MAR | No | Yes | Yes | rbcL, ITS2, rpoB, rpoC1, psbK-psbI |
| Poales | Cyperaceae | *Scleria gaertneri* Raddi | CANGI159-17 | Pastore M. 537 | No | No | Yes | rbcL, ITS2 |
| Poales | Cyperaceae | *Scleria latifolia* Sw. | CANGA253-17 | Nunes C.S. 92 | No | Yes | No | rbcL, atpF-atpH |
| Poales | Cyperaceae | *Scleria latifolia* Sw. | CANGI157-17 | Harley R.M. 57938 | No | No | No | rbcL |
| Poales | Cyperaceae | *Scleria secans* (L.) Urb. | CANGI155-17 | Gil A. 520 | Yes | Yes | Yes | rbcL, ITS2, atpF-atpH |
| Poales | Cyperaceae | *Scleria secans* (L.) Urb. | CANGA386-17 | Praia T.S. 26 | Yes | Yes | Yes | rbcL, rpoC1 |
| Poales | Cyperaceae | *Scleria verticillata* Muhl. ex Willd. | CGII609-20 | Zappi D.C. 4279 | Yes | No | No | rbcL, ITS2 |
| Poales | Eriocaulaceae | *Eriocaulon* aff. *setaceum* L. | CANGI180-17 | Harley R.M. 57314 | Yes | Yes | Yes | rbcL, ITS2, rpoC1, atpF-atpH, psbK-psbI |
| Poales | Eriocaulaceae | *Eriocaulon* aff. *setaceum* L. | CANGI183-17 | Vasconcelos L.V. 785 | Yes | Yes | Yes | rbcL, ITS2 |
| Poales | Eriocaulaceae | *Eriocaulon carajense* Moldenke | CANGA085-17 | Harley R.M. 57269 | Yes | Yes | Yes | rbcL, ITS2, rpoC1, atpF-atpH, psbK-psbI |
| Poales | Eriocaulaceae | *Eriocaulon carajense* Moldenke | CANGA086-17 | Mota N.F.O. 3416 | Yes | Yes | Yes | rbcL, rpoB, rpoC1, psbK-psbI |
| Poales | Eriocaulaceae | *Eriocaulon carajense* Moldenke | CANGA090-17 | Viana P.L. 6164 | Yes | Yes | Yes | rbcL, ITS2 |
| Poales | Eriocaulaceae | *Eriocaulon carajense* Moldenke | CANGA088-17 | Pastore M. 648 | Yes | No | Yes | rbcL, ITS2 |
| Poales | Eriocaulaceae | *Eriocaulon carajense* Moldenke | CANGA089-17 | Pastore M. 620 | Yes | No | Yes | rbcL, ITS2 |
| Poales | Eriocaulaceae | *Eriocaulon cinereum* R.Br. | CANGA140-17 | Viana P.L. 5633 | Yes | Yes | Yes | rbcL, ITS2, atpF-atpH, trnH-psbA |
| Poales | Eriocaulaceae | *Eriocaulon cinereum* R.Br. | CANGA141-17 | Harley R.M. 57268 | Yes | Yes | Yes | rbcL, ITS2, rpoB, rpoC1, atpF-atpH, psbK-psbI |
| Poales | Eriocaulaceae | *Eriocaulon cinereum* R.Br. | CANGI181-17 | Harley R.M. 57303 | Yes | Yes | Yes | rbcL, ITS2, matK, rpoB, rpoC1, atpF-atpH, psbK-psbI |
| Poales | Eriocaulaceae | *Eriocaulon cinereum* R.Br. | CANGA132-17 | Harley R.M. 57283 | Yes | Yes | Yes | rbcL, ITS2, matK, rpoB, rpoC1, atpF-atpH, psbK-psbI |
| Poales | Eriocaulaceae | *Eriocaulon cinereum* R.Br. | CANGA145-17 | Vasconcelos L.V. 808 | Yes | Yes | Yes | rbcL, ITS2, trnH-psbA |
| Poales | Eriocaulaceae | *Eriocaulon cinereum* R.Br. | CANGA146-17 | Vasconcelos L.V. 814 | Yes | Yes | Yes | rbcL, ITS2 |
| Poales | Eriocaulaceae | *Eriocaulon cinereum* R.Br. | CANGA147-17 | Harley R.M. 58107 | Yes | No | Yes | rbcL, ITS2 |
| Poales | Eriocaulaceae | *Paepalanthus* cf. *bifidus* (Schrad.) Kunth | CGII612-20 | Zappi D.C. 4291 | No | No | Yes | rbcL |
| Poales | Eriocaulaceae | *Paepalanthus fasciculatus* (Rottb.) Kunth | CGII635-20 | Zappi D.C. 4366 | No | No | No | rbcL, ITS2 |
| Poales | Eriocaulaceae | *Paepalanthus fasciculoides* Hensold | CANGA180-17 | Viana P.L. 5563 | Yes | Yes | Yes | rbcL, ITS2 |
| Poales | Eriocaulaceae | *Paepalanthus fasciculoides* Hensold | CANGA181-17 | Cardoso A. 1940 | Yes | Yes | Yes | rbcL, ITS2, rpoB, rpoC1, atpF-atpH, trnH-psbA |
| Poales | Eriocaulaceae | *Paepalanthus fasciculoides* Hensold | CANGA182-17 | Cardoso A. 2006 | Yes | Yes | Yes | rbcL, ITS2, rpoB, rpoC1, atpF-atpH, trnH-psbA |
| Poales | Eriocaulaceae | *Paepalanthus fasciculoides* Hensold | CANGA183-17 | Cardoso A. 2018 | Yes | Yes | Yes | rbcL, atpF-atpH, trnH-psbA |
| Poales | Eriocaulaceae | *Paepalanthus fasciculoides* Hensold | CANGA185-17 | Harley R.M. 57439 | Yes | Yes | Yes | rbcL, ITS2 |
| Poales | Eriocaulaceae | *Paepalanthus fasciculoides* Hensold | CANGA189-17 | Viana P.L. 6173 | Yes | Yes | Yes | rbcL, ITS2 |
| Poales | Eriocaulaceae | *Paepalanthus fasciculoides* Hensold | CANGA186-17 | Pastore M. 663 | Yes | No | Yes | rbcL, ITS2 |
| Poales | Eriocaulaceae | *Paepalanthus fasciculoides* Hensold | CANGA190-17 | Viana P.L. 6104 | Yes | Yes | Yes | rbcL, ITS2 |
| Poales | Eriocaulaceae | *Paepalanthus fasciculoides* Hensold | CANGA187-17 | Vasconcelos L.V. 1085 | Yes | No | Yes | rbcL, ITS2 |
| Poales | Eriocaulaceae | *Paepalanthus fasciculoides* Hensold | CANGA188-17 | Vasconcelos L.V. 1132 | Yes | No | Yes | rbcL, ITS2 |
| Poales | Eriocaulaceae | *Paepalanthus fasciculoides* Hensold | CANGA179-17 | Harley R.M. 58084 | Yes | No | Yes | rbcL |
| Poales | Eriocaulaceae | *Paepalanthus polytrichoides* Kunth | CGII622-20 | Zappi D.C. 4322 | No | No | Yes | rbcL, ITS2 |
| Poales | Eriocaulaceae | *Paepalanthus subtilis* Miq. | CGII618-20 | Zappi D.C. 4309 | No | No | Yes | rbcL |
| Poales | Eriocaulaceae | *Syngonanthus* aff. *saxicola* (Körn.) Trovó & Stützel | CANGI185-17 | Mota N.F.O. 3415 | Yes | Yes | Yes | rbcL, ITS2, matK, rpoC1, atpF-atpH, psbK-psbI |
| Poales | Eriocaulaceae | *Syngonanthus caulescens* (Poir.) Ruhland | CANGA126-17 | Harley R.M. 57267 | Yes | Yes | Yes | rbcL, ITS2, matK, rpoC1, atpF-atpH, psbK-psbI |
| Poales | Eriocaulaceae | *Syngonanthus caulescens* (Poir.) Ruhland | CANGA116-17 | Pastore M. 463 | Yes | Yes | Yes | rbcL, ITS2 |
| Poales | Eriocaulaceae | *Syngonanthus caulescens* (Poir.) Ruhland | CGII182-20 | Zappi D.C. 4442 | Yes | No | Yes | rbcL |
| Poales | Eriocaulaceae | *Syngonanthus davidsei* Huft | CGII621-20 | Zappi D.C. 4321 | No | No | Yes | rbcL, ITS2 |
| Poales | Eriocaulaceae | *Syngonanthus discretifolius* (Moldenke) M.T.C.Watan. | CANGA439-17 | Viana P.L. 5685 | Yes | Yes | Yes | rbcL, ITS2, rpoC1, atpF-atpH, trnH-psbA |
| Poales | Eriocaulaceae | *Syngonanthus discretifolius* (Moldenke) M.T.C.Watan. | CANGA115-17 | Cardoso A. 1955 | Yes | Yes | Yes | rbcL, ITS2, rpoC1, trnH-psbA |
| Poales | Eriocaulaceae | *Syngonanthus discretifolius* (Moldenke) M.T.C.Watan. | CANGI259-17 | Vasconcelos L.V. 787 | Yes | Yes | Yes | rbcL, ITS2, trnH-psbA |
| Poales | Eriocaulaceae | *Syngonanthus discretifolius* (Moldenke) M.T.C.Watan. | CANGA160-17 | Vasconcelos L.V. 801 | Yes | Yes | Yes | rbcL, ITS2 |
| Poales | Eriocaulaceae | *Syngonanthus discretifolius* (Moldenke) M.T.C.Watan. | CANGA161-17 | Viana P.L. 6119 | Yes | Yes | Yes | rbcL, ITS2 |
| Poales | Eriocaulaceae | *Syngonanthus discretifolius* (Moldenke) M.T.C.Watan. | CANGA162-17 | Viana P.L. 6168 | Yes | Yes | Yes | rbcL, ITS2 |
| Poales | Eriocaulaceae | *Syngonanthus discretifolius* (Moldenke) M.T.C.Watan. | CANGA163-17 | Pastore M. 642 | Yes | No | Yes | rbcL |
| Poales | Eriocaulaceae | *Syngonanthus discretifolius* (Moldenke) M.T.C.Watan. | CANGI187-17 | Harley R.M. 57910 | Yes | Yes | Yes | rbcL, ITS2 |
| Poales | Eriocaulaceae | *Syngonanthus discretifolius* (Moldenke) M.T.C.Watan. | CANGI188-17 | Harley R.M. 58128 | Yes | No | Yes | rbcL, ITS2 |
| Poales | Eriocaulaceae | *Syngonanthus heteropeplus* (Koern.) Ruhland | CANGA227-17 | Pastore M. 583 | Yes | No | Yes | rbcL |
| Poales | Eriocaulaceae | *Syngonanthus heteropeplus* (Koern.) Ruhland | CGII623-20 | Zappi D.C. 4323 | Yes | No | Yes | rbcL |
| Poales | Eriocaulaceae | *Syngonanthus humboldtii* (Kunth) Ruhland | CGII639-20 | Zappi D.C. 4386 | No | No | Yes | rbcL, ITS2 |
| Poales | Eriocaulaceae | *Syngonanthus nitens* Ruhland | CANGA317-17 | Pastore M. 462 | No | Yes | Yes | rbcL, ITS2 |
| Poales | Eriocaulaceae | *Syngonanthus nitens* Ruhland | CGII179-20 | Zappi D.C. 4450 | No | No | Yes | rbcL |
| Poales | Eriocaulaceae | *Syngonanthus simplex* (Miq.) Ruhland | CANGA394-17 | Harley R.M. 58117 | Yes | No | Yes | rbcL, ITS2 |
| Poales | Eriocaulaceae | *Syngonanthus tenuis* (Kunth) Ruhland | CGII641-20 | Zappi D.C. 4397 | No | No | Yes | rbcL |
| Poales | Eriocaulaceae | *Syngonanthus umbellatus* (Lam.) Ruhland | CGII634-20 | Zappi D.C. 4363 | No | No | Yes | rbcL, ITS2 |
| Poales | Mayacaceae | *Mayaca fluviatilis* Aubl. | CANGA202-17 | Mota N.F.O. 3408 | Yes | Yes | No | rbcL, ITS2, matK, rpoB, rpoC1, atpF-atpH |
| Poales | Mayacaceae | *Mayaca fluviatilis* Aubl. | CANGI344-17 | Vasconcelos L.V. 828 | Yes | Yes | No | rbcL, ITS2 |
| Poales | Mayacaceae | *Mayaca fluviatilis* Aubl. | CANGI345-17 | Rocha K. 91 | Yes | Yes | No | rbcL, ITS2 |
| Poales | Mayacaceae | *Mayaca kunthii* Seub. | CANGI341-17 | Cardoso A. 2005 | Yes | Yes | Yes | rpoB, atpF-atpH, trnH-psbA |
| Poales | Mayacaceae | *Mayaca kunthii* Seub. | CANGA247-17 | Mota N.F.O. 3410 | Yes | Yes | Yes | rbcL, ITS2, matK, atpF-atpH |
| Poales | Mayacaceae | *Mayaca longipes* Gand. | CANGI346-17 | Harley R.M. 57482 | Yes | Yes | Yes | rbcL, ITS2 |
| Poales | Poaceae | *Actinocladum verticillatum* (Nees) McClure ex Soderstr. | CANGA433-17 | Pereira S.S. 2 | No | Yes | Yes | rbcL, ITS2, matK, atpF-atpH, psbK-psbI |
| Poales | Poaceae | *Anthaenantia lanata* (Kunth) Benth. | CANGA248-17 | Vasconcelos L.V. 1059 | Yes | No | No | ITS2 |
| Poales | Poaceae | *Arthrostylidium scandens* McClure | CANGA384-17 | Viana P.L. s.n. | No | Yes | Yes | rbcL, matK, atpF-atpH, psbK-psbI |
| Poales | Poaceae | *Axonopus aureus* P. Beauv. | CGII628-20 | Zappi D.C. 4343 | Yes | No | No | rbcL, ITS2 |
| Poales | Poaceae | *Axonopus leptostachyus* (Flüggé) Hitchc. | CANGA257-17 | Lopes C.S.A. 8 | No | Yes | No | rbcL |
| Poales | Poaceae | *Axonopus leptostachyus* (Flüggé) Hitchc. | CANGI447-17 | Praia T.S. 28 | No | Yes | No | ITS2, rpoB, atpF-atpH |
| Poales | Poaceae | *Axonopus longispicus* (Döll) Kuhlm. | CANGA256-17 | Afonso E.A.L 109 | Yes | Yes | Yes | ITS2, atpF-atpH |
| Poales | Poaceae | *Axonopus longispicus* (Döll) Kuhlm. | CANGI448-17 | Viana P.L. 6197 | Yes | Yes | Yes | ITS2 |
| Poales | Poaceae | *Axonopus rupestris* Davidse | CANGA379-17 | Viana P.L. 5607 | Yes | Yes | Yes | rbcL, ITS2, matK, rpoB, atpF-atpH, psbK-psbI, trnH-psbA |
| Poales | Poaceae | *Axonopus rupestris* Davidse | CANGA138-17 | Viana P.L. 6224 | Yes | Yes | Yes | rbcL |
| Poales | Poaceae | *Axonopus rupestris* Davidse | CANGA380-17 | Harley R.M. 57500 | Yes | Yes | Yes | rbcL, ITS2 |
| Poales | Poaceae | *Axonopus rupestris* Davidse | CANGI481-17 | Harley R.M. 58125 | Yes | No | Yes | rbcL, ITS2 |
| Poales | Poaceae | *Eragrostis maypurensis* (Kunth) Steud. | CANGI449-17 | Viana P.L. 6150 | Yes | Yes | Yes | rbcL, ITS2 |
| Poales | Poaceae | *Guadua paniculata* Munro | CANGA328-17 | Viana P.L. s.n. | No | Yes | No | rbcL, ITS2, rpoB, atpF-atpH, psbK-psbI, trnH-psbA |
| Poales | Poaceae | *Hildaea breviscrobs* (Döll) C.Silva & R.P. Oliveira | CANGI275-17 | Trindade J.R. 360 | Yes | Yes | Yes | ITS2, matK, rpoB, atpF-atpH, psbK-psbI |
| Poales | Poaceae | *Hildaea* sp. | CANGI454-17 | Viana P.L. 6124 | Yes | Yes | Yes | rbcL, ITS2 |
| Poales | Poaceae | *Hildaea tenuis* (J. Presl & C.Presl) C.Silva & R.P.Oliveira | CANGI475-17 | Harley R.M. 58057 | Yes | No | Yes | rbcL, ITS2 |
| Poales | Poaceae | *Hildaea tenuis* (J. Presl & C.Presl) C.Silva & R.P.Oliveira | CANGI478-17 | Harley R.M. 58090 | Yes | No | Yes | rbcL, ITS2 |
| Poales | Poaceae | *Ichnanthus calvescens* (Nees ex Trin.) Döll | CANGA047-17 | Viana P.L. 6195 | Yes | Yes | No | rbcL, ITS2 |
| Poales | Poaceae | *Luziola peruviana* Juss. ex J.F.Gmel. | CANGI457-17 | Viana P.L. 6156 | Yes | Yes | No | rbcL, ITS2 |
| Poales | Poaceae | *Mesosetum cayennense* Steud. | CANGI466-17 | Viana P.L. 6117 | Yes | Yes | Yes | rbcL, ITS2 |
| Poales | Poaceae | *Mesosetum cayennense* Steud. | CANGI477-17 | Harley R.M. 58089 | Yes | No | Yes | rbcL, ITS2 |
| Poales | Poaceae | *Olyra caudata* Trin. | CANGA113-17 | Pereira S.S. 6 | No | Yes | No | rbcL, matK, rpoC1, psbK-psbI, trnH-psbA |
| Poales | Poaceae | *Olyra caudata* Trin. | CANGA114-17 | Pereira S.S. 6 | No | Yes | No | rbcL, ITS2, atpF-atpH, psbK-psbI |
| Poales | Poaceae | *Olyra latifolia* L. | CANGA254-17 | Pereira S.S. 4 | Yes | Yes | No | rbcL, matK, atpF-atpH, psbK-psbI, trnH-psbA |
| Poales | Poaceae | *Olyra latifolia* L. | CANGI458-17 | Afonso E.A.L 135 | Yes | Yes | No | rbcL, ITS2, matK, atpF-atpH, psbK-psbI |
| Poales | Poaceae | *Olyra latifolia* L. | CANGI472-17 | Pastore M. 516 | Yes | No | No | rbcL, ITS2 |
| Poales | Poaceae | *Olyra latifolia* L. | CANGI473-17 | Pastore M. 533 | Yes | No | No | rbcL |
| Poales | Poaceae | *Oryza rufipogon* Griff. | CANGI459-17 | Pastore M. 626 | No | No | No | rbcL, ITS2 |
| Poales | Poaceae | *Panicum* aff. *arctum* | CANGI462-17 | Harley R.M. 58122 | No | No | Yes | rbcL, ITS2 |
| Poales | Poaceae | *Panicum millegrana* Poir. | CANGI460-17 | Costa J.L.C. 4 | Yes | Yes | No | rbcL, matK, rpoB, atpF-atpH, psbK-psbI |
| Poales | Poaceae | *Panicum millegrana* Poir. | CANGI461-17 | Costa J.L.C. 4 | Yes | Yes | No | ITS2, atpF-atpH, psbK-psbI, trnH-psbA |
| Poales | Poaceae | *Paratheria prostrata* Griseb. | CANGA359-17 | Trindade J.R. 372 | Yes | Yes | Yes | ITS2, matK, atpF-atpH, psbK-psbI |
| Poales | Poaceae | *Pariana intermedia* Döll | CANGI464-17 | Harley R.M. 58056 | No | No | Yes | rbcL, ITS2 |
| Poales | Poaceae | *Parodiolyra luetzelburgii* (Pilg.) Soderstr. & Zuloaga | CANGA289-17 | Pereira S.S. 1 | No | Yes | Yes | rbcL, ITS2, matK, atpF-atpH, psbK-psbI, trnH-psbA |
| Poales | Poaceae | *Parodiolyra luetzelburgii* (Pilg.) Soderstr. & Zuloaga | CANGA266-17 | Pereira S.S. 1 | No | Yes | Yes | rbcL, ITS2, matK, atpF-atpH, psbK-psbI |
| Poales | Poaceae | *Paspalum axillare* Swallen | CANGA028-17 | Viana P.L. 6130 | Yes | Yes | Yes | rbcL, ITS2 |
| Poales | Poaceae | *Paspalum foliiforme* S.Denham | CANGA203-17 | Viana P.L. 6128 | Yes | Yes | Yes | rbcL |
| Poales | Poaceae | *Paspalum foliiforme* S.Denham | CANGI467-17 | Viana P.L. 6165 | Yes | Yes | Yes | rbcL, ITS2 |
| Poales | Poaceae | *Paspalum reticulinerve* Renvoize | CANGI468-17 | Viana P.L. 6166 | Yes | Yes | Yes | rbcL, ITS2 |
| Poales | Poaceae | *Paspalum reticulinerve* Renvoize | CANGI469-17 | Harley R.M. 58099 | Yes | No | Yes | rbcL, ITS2 |
| Poales | Poaceae | *Raddiella esenbeckii* (Steud.) C.E.Calderón & Soderstr. | CANGA176-17 | Pastore M. 458 | No | Yes | Yes | rbcL, ITS2, trnH-psbA |
| Poales | Poaceae | *Rhytachne gonzalezii* Davidse | CANGA218-17 | Harley R.M. 57452 | Yes | Yes | Yes | rbcL, ITS2 |
| Poales | Poaceae | *Rhytachne gonzalezii* Davidse | CANGA216-17 | Vasconcelos L.V. 823 | Yes | Yes | Yes | rbcL, ITS2 |
| Poales | Poaceae | *Rhytachne gonzalezii* Davidse | CANGA217-17 | Viana P.L. 6127 | Yes | Yes | Yes | rbcL, ITS2 |
| Poales | Poaceae | *Rhytachne gonzalezii* Davidse | CANGI470-17 | Viana P.L. 6233 | Yes | Yes | Yes | rbcL, ITS2 |
| Poales | Poaceae | *Sacciolepis myuros* (Lam.) Chase | CGII617-20 | Zappi D.C. 4308 | Yes | No | Yes | rbcL, ITS2 |
| Poales | Poaceae | *Schizachyrium* sp. | CGII629-20 | Zappi D.C. 4352 | No | No | Yes | rbcL, ITS2 |
| Poales | Poaceae | *Sporobolus multiramosus* Longhi-Wagner & Boechat | CANGA302-17 | Viana P.L. 5576 | Yes | Yes | Yes | matK, atpF-atpH, trnH-psbA |
| Poales | Poaceae | *Sporobolus multiramosus* Longhi-Wagner & Boechat | CANGA305-17 | Viana P.L. 6225 | Yes | Yes | Yes | rbcL, ITS2 |
| Poales | Poaceae | *Trachypogon spicatus* (L.f.) Kuntze | CANGI476-17 | Harley R.M. 58080 | Yes | No | No | rbcL, ITS2 |
| Poales | Poaceae | *Trichanthecium nervosum* (Lam.) Zuloaga & Morrone | CGII642-20 | Zappi D.C. 4401 | No | No | Yes | rbcL, ITS2 |
| Poales | Poaceae | *Tristachya chrysothrix* Nees | CANGI480-17 | Harley R.M. 58100 | Yes | No | Yes | rbcL, ITS2 |
| Poales | Xyridaceae | *Xyris brachysepala* Kral | CANGA036-17 | Mota N.F.O. 3409 | Yes | Yes | Yes | rbcL, ITS2 |
| Poales | Xyridaceae | *Xyris brachysepala* Kral | CANGA037-17 | Mota N.F.O. 2966 | Yes | Yes | Yes | ITS2, rpoC1, atpF-atpH |
| Poales | Xyridaceae | *Xyris brachysepala* Kral | CANGA039-17 | Vasconcelos L.V. 765 | Yes | Yes | Yes | rbcL, ITS2 |
| Poales | Xyridaceae | *Xyris brachysepala* Kral | CANGA038-17 | Viana P.L. 6137 | Yes | Yes | Yes | rbcL, ITS2 |
| Poales | Xyridaceae | *Xyris brachysepala* Kral | CANGI618-17 | Harley R.M. 58114 | Yes | No | Yes | rbcL, ITS2 |
| Poales | Xyridaceae | *Xyris fallax* Malme | CANGA177-17 | Pastore M. 460 | No | Yes | Yes | rbcL |
| Poales | Xyridaceae | *Xyris macrocephala* Vahl | CANGA269-17 | Harley R.M. 57878 | Yes | No | No | rbcL |
| Polypodiales | Dryopteridaceae | *Cyclodium inerme* (Fée) A.R.Sm. | CANGI176-17 | Harley R.M. 57947 | Yes | No | Yes | rbcL |
| Polypodiales | Dryopteridaceae | *Dryopteris huberi* (Christ) C.Chr. | CANGI178-17 | Harley R.M. 57947 | Yes | No | No | ITS2 |
| Polypodiales | Dryopteridaceae | *Triplophyllum glabrum* J.Prado & R.C.Moran | CANGI598-17 | Harley R.M. 57889 | No | No | Yes | rbcL |
| Polypodiales | Polypodiaceae | *Microgramma nana* (Liebm.) T.E.Almeida | CANGI491-17 | Viana P.L. 6167 | No | Yes | Yes | rbcL |
| Polypodiales | Polypodiaceae | *Serpocaulon triseriale* (Sw.) A.R.Sm. | CANGI492-17 | Harley R.M. 57451 | Yes | Yes | No | rbcL |
| Polypodiales | Polypodiaceae | *Serpocaulon triseriale* (Sw.) A.R.Sm. | CANGI493-17 | Praia T.S. 13 | Yes | Yes | No | rbcL |
| Polypodiales | Pteridaceae | *Ceratopteris thalictroides* (L.) Brongn. | CANGI508-17 | Trindade J.R. 235 | Yes | Yes | No | ITS2 |
| Polypodiales | Pteridaceae | *Ceratopteris thalictroides* (L.) Brongn. | CANGI509-17 | Pastore M. 558 | Yes | No | No | ITS2 |
| Polypodiales | Pteridaceae | *Hemionitis palmata* L. | CANGI510-17 | Harley R.M. 57408 | Yes | Yes | No | trnH-psbA |
| Proteales | Proteaceae | *Roupala montana* Aubl. | CANGA133-17 | Trindade J.R. 231 | Yes | Yes | No | rbcL, ITS2, matK, rpoB, rpoC1, atpF-atpH, psbK-psbI |
| Proteales | Proteaceae | *Roupala montana* Aubl. | CANGA449-20 | Harley R.M. 57957 | Yes | Yes | No | rbcL |
| Ranunculales | Menispermaceae | *Abuta grandifolia* (Mart.) Sandwith | CANGI355-17 | Gil A. 540 | Yes | Yes | No | rbcL, matK, rpoB, rpoC1, psbK-psbI |
| Ranunculales | Menispermaceae | *Abuta grandifolia* (Mart.) Sandwith | CANGA221-17 | Lima M.E.L. 2574 | Yes | No | No | rbcL |
| Ranunculales | Menispermaceae | *Cissampelos laxiflora* Moldenke | CANGI354-17 | Rocha K. 59 | Yes | Yes | Yes | rbcL, ITS2, matK, rpoB, rpoC1, atpF-atpH, psbK-psbI |
| Ranunculales | Ranunculaceae | *Clematis brasiliana* DC. | CANGA156-17 | Harley R.M. 57422 | Yes | Yes | Yes | ITS2, matK, rpoB, rpoC1, trnH-psbA |
| Rosales | Cannabaceae | *Trema micrantha* (L.) Blume | CANGA287-17 | Prado M.L 370 | Yes | Yes | No | rbcL, rpoB, atpF-atpH, psbK-psbI |
| Rosales | Moraceae | *Brosimum gaudichaudii* Trécul | CGII657-20 | Zappi D.C. 4537 | No | No | Yes | rbcL, ITS2 |
| Rosales | Moraceae | *Ficus americana* subsp. *guianensis* (Desv.) C.C. Berg | CANGI358-17 | Reis A.S. 28 | Yes | Yes | No | ITS2, trnH-psbA |
| Rosales | Rhamnaceae | *Gouania pyrifolia* Reissek | CANGI513-17 | Viana P.L. 6172 | No | Yes | Yes | rbcL, ITS2 |
| Rosales | Urticaceae | *Laportea aestuans* (L.) Chew | CANGI601-17 | Pastore M. 530 | Yes | No | No | ITS2 |
| Rosales | Urticaceae | *Pilea microphylla* (L.) Liebm. | CANGI599-17 | Harley R.M. 57508 | No | Yes | No | rbcL, ITS2 |
| Santalales | Loranthaceae | *Passovia pedunculata* (Jacq.) Kuijt | CANGI303-17 | N. Mota 3420 | Yes | Yes | Yes | ITS2, matK, rpoB, rpoC1, psbK-psbI |
| Santalales | Loranthaceae | *Passovia pedunculata* (Jacq.) Kuijt | CANGI302-17 | Harley R.M. 57971 | Yes | No | Yes | rbcL, ITS2 |
| Santalales | Loranthaceae | *Passovia pyrifolia* (Kunth) Tiegh. | CANGA454-20 | Harley R.M. 57851 | Yes | No | Yes | rbcL, ITS2 |
| Santalales | Loranthaceae | *Struthanthus marginatus* (Desr.) G.Don | CANGA212-17 | Pastore M. 361 | Yes | Yes | Yes | rbcL, ITS2, trnH-psbA |
| Santalales | Loranthaceae | *Struthanthus polyrhizus* (Mart.) Mart. | CANGI562-17 | Harley R.M. 57908 | Yes | Yes | Yes | ITS2 |
| Santalales | Olacaceae | *Heisteria ovata* Benth. | CANGI392-17 | Viana P.L. 6232 | Yes | Yes | Yes | rbcL, ITS2 |
| Santalales | Olacaceae | *Heisteria ovata* Benth. | CGII640-20 | Zappi D.C. 4392 | Yes | No | Yes | rbcL, ITS2 |
| Santalales | Olacaceae | *Heisteria ovata* Benth. | CGII184-20 | Zappi D.C. 4444 | Yes | No | Yes | rbcL, ITS2 |
| Santalales | Olacaceae | *Ximenia americana* L. | CANGI194-17 | Vasconcelos L.V. 883 | Yes | Yes | No | rbcL, ITS2, trnH-psbA |
| Santalales | Opiliaceae | *Agonandra silvatica* Ducke | CANGA391-17 | Harley R.M. 57895 | Yes | No | No | rbcL, ITS2 |
| Santalales | Opiliaceae | *Agonandra silvatica* Ducke | CANGA392-17 | Vasconcelos L.V. 924 | Yes | No | No | rbcL, ITS2 |
| Santalales | Opiliaceae | *Agonandra silvatica* Ducke | CANGA393-17 | Vasconcelos L.V. 1040 | Yes | No | No | rbcL, ITS2 |
| Santalales | Santalaceae | *Phoradendron* cf. *obtusissimum* (Miq.) Eichler | CANGA472-20 | Giulietti A.M. 2655 | Yes | No | Yes | rbcL |
| Santalales | Santalaceae | *Phoradendron crassifolium* (Pohl ex DC.) Eichler | CANGI563-17 | Harley R.M. 57880 | Yes | No | Yes | rbcL |
| Santalales | Santalaceae | *Phoradendron quadrangulare* (Kunth) Griseb. | CANGI556-17 | Harley R.M. 57332 | Yes | Yes | No | rbcL, matK |
| Santalales | Santalaceae | *Phoradendron quadrangulare* (Kunth) Griseb. | CANGI555-17 | Reis A.S. 26 | Yes | Yes | No | rbcL, rpoB, rpoC1, atpF-atpH |
| Santalales | Santalaceae | *Phoradendron quadrangulare* (Kunth) Griseb. | CANGI558-17 | Lopes C.S.A. 4 | Yes | Yes | No | rbcL, atpF-atpH |
| Santalales | Santalaceae | *Phoradendron quadrangulare* (Kunth) Griseb. | CANGI559-17 | Harley R.M. 57890 | Yes | No | No | rbcL |
| Santalales | Santalaceae | *Phoradendron tunaeforme* (DC.) Eichler | CANGI560-17 | Reis A.S. 21 | Yes | Yes | Yes | rbcL |
| Santalales | Santalaceae | *Phoradendron tunaeforme* (DC.) Eichler | CANGI564-17 | Vasconcelos L.V. 1022 | Yes | No | Yes | rbcL, ITS2 |
| Sapindales | Anacardiaceae | *Anacardium occidentale* L. | CANGI014-17 | Harley R.M. 57328 | Yes | Yes | No | rbcL, ITS2, matK, rpoB, psbK-psbI |
| Sapindales | Burseraceae | *Protium aracouchini* (Aubl.) Marchand | CANGI090-17 | Harley R.M. 57923 | No | No | Yes | rbcL |
| Sapindales | Burseraceae | *Protium heptaphyllum* (Aubl.) Marchand | CANGI092-17 | Giulietti A.M. 2611 | No | No | Yes | rbcL |
| Sapindales | Burseraceae | *Protium sagotianum* Marchand | CANGI091-17 | Harley R.M. 57937 | No | No | No | rbcL, ITS2 |
| Sapindales | Meliaceae | *Trichilia micrantha* Benth. | CANGA288-17 | Harley R.M. 57884 | Yes | No | No | rbcL |
| Sapindales | Meliaceae | *Trichilia rubra* C.DC. | CANGI353-17 | Harley R.M. 57965 | No | Yes | Yes | rbcL, ITS2 |
| Sapindales | Meliaceae | *Trichilia rubra* C.DC. | CANGI112-17 | Vasconcelos L.V. 936 | No | No | Yes | ITS2 |
| Sapindales | Rutaceae | *Dictyoloma vandellianum* A.Juss. | CANGI547-17 | Viana P.L. 6148 | Yes | Yes | No | rbcL, ITS2 |
| Sapindales | Rutaceae | *Ertela trifolia* (L.) Kuntze | CANGA427-17 | Viana P.L. 6200 | Yes | Yes | Yes | rbcL, ITS2 |
| Sapindales | Rutaceae | *Ertela trifolia* (L.) Kuntze | CANGI548-17 | Harley R.M. 58070 | Yes | No | Yes | rbcL, ITS2 |
| Sapindales | Rutaceae | *Esenbeckia cowanii* Kaastra | CANGA450-20 | Harley R.M. 57995 | Yes | Yes | Yes | rbcL, ITS2 |
| Sapindales | Rutaceae | *Esenbeckia grandiflora* Mart. | CANGI549-17 | Meirelles J. 957 | No | Yes | Yes | rbcL, ITS2 |
| Sapindales | Rutaceae | *Esenbeckia grandiflora* Mart. | CANGI552-17 | Rocha K. 60 | No | Yes | Yes | rbcL |
| Sapindales | Rutaceae | *Metrodorea flavida* K.Krause | CANGI553-17 | Nunes C.S. 101 | No | Yes | Yes | ITS2, rpoB |
| Sapindales | Rutaceae | *Pilocarpus carajaensis* Skorupa | CANGA057-17 | Viana P.L. 5669 | Yes | Yes | Yes | rbcL, matK, rpoB, rpoC1, atpF-atpH, psbK-psbI, trnH-psbA |
| Sapindales | Rutaceae | *Pilocarpus carajaensis* Skorupa | CANGA082-17 | Harley R.M. 57258 | Yes | Yes | Yes | rbcL, ITS2, matK, rpoB, rpoC1, atpF-atpH, psbK-psbI |
| Sapindales | Rutaceae | Pilocarpus carajaensis Skorupa | CANGA083-17 | Harley R.M. 57952 | Yes | Yes | Yes | rbcL, ITS2 |
| Sapindales | Rutaceae | *Pilocarpus microphyllus* Stapf ex Wardlew. | CANGA293-17 | Harley R.M. 57999 | Yes | Yes | Yes | rbcL, ITS2 |
| Sapindales | Rutaceae | *Pilocarpus microphyllus* Stapf ex Wardlew. | CANGA294-17 | Vasconcelos L.V. 1117 | Yes | No | Yes | rbcL, ITS2 |
| Sapindales | Rutaceae | *Rauia* cf. *prancei* W.A.Rodrigues & M.F.Silva | CANGI551-17 | Afonso E.A.L 138 | No | Yes | Yes | rbcL, ITS2, rpoB, rpoC1, psbK-psbI |
| Sapindales | Rutaceae | *Zanthoxylum rhoifolium* Lam. | CANGI550-17 | Nogueira M.G.C. 670 | No | No | Yes | rbcL |
| Sapindales | Sapindaceae | *Matayba guianensis* Aubl. | CANGI567-17 | Costa J.L.C. 11 | Yes | Yes | No | rbcL, ITS2, matK, rpoB, rpoC1, atpF-atpH, psbK-psbI |
| Sapindales | Sapindaceae | *Matayba guianensis* Aubl. | ITVRT018-17 | Vasconcelos L.V. 926 | Yes | No | No | rbcL, ITS2 |
| Sapindales | Sapindaceae | *Pseudima frutescens* (Aubl.) Radlk. | CANGI569-17 | Viana P.L. 6151 | No | Yes | Yes | rbcL, ITS2 |
| Sapindales | Sapindaceae | *Pseudima frutescens* (Aubl.) Radlk. | CANGI566-17 | Harley R.M. 57920 | No | No | Yes | rbcL |
| Sapindales | Sapindaceae | *Pseudima frutescens* (Aubl.) Radlk. | CANGI571-17 | Harley R.M. 58050 | No | No | Yes | rbcL, ITS2 |
| Sapindales | Sapindaceae | *Serjania caracasana* (Jacq.) Willd. | CANGA467-20 | Harley R.M. 57881 | Yes | No | Yes | rbcL, ITS2 |
| Sapindales | Sapindaceae | *Serjania caracasana* (Jacq.) Willd. | CANGI570-17 | Harley R.M. 57918 | Yes | No | Yes | rbcL |
| Sapindales | Sapindaceae | *Serjania caracasana* (Jacq.) Willd. | ITVRT016-17 | Harley R.M. 57926 | Yes | No | Yes | rbcL |
| Sapindales | Sapindaceae | *Serjania caracasana* (Jacq.) Willd. | ITVRT017-17 | Harley R.M. 57927 | Yes | No | Yes | rbcL |
| Sapindales | Sapindaceae | *Serjania confertiflora* Radlk. | CANGI568-17 | Rocha K. 54 | Yes | Yes | Yes | rbcL, ITS2, rpoB, rpoC1, atpF-atpH |
| Sapindales | Simaroubaceae | *Simaba guianensis* Aubl. | CGII611-20 | Zappi D.C. 4282 | Yes | No | No | rbcL |
| Sapindales | Simaroubaceae | *Simarouba amara* Aubl. | CANGA011-17 | Harley R.M. 57894 | Yes | No | No | rbcL, ITS2 |
| Sapindales | Simaroubaceae | *Simarouba amara* Aubl. | CANGA012-17 | Giulietti A.M. 2637 | Yes | No | No | rbcL |
| Sapindales | Simaroubaceae | *Simarouba amara* Aubl. | CANGA013-17 | Vasconcelos L.V. 1030 | Yes | No | No | rbcL |
| Solanales | Convolvulaceae | *Aniseia martinicensis* (Jacq.) Choisy | CANGA123-17 | Mota N.F.O. 2987 | Yes | Yes | No | ITS2, rpoC1, atpF-atpH |
| Solanales | Convolvulaceae | *Aniseia martinicensis* (Jacq.) Choisy | CANGA122-17 | Pastore M. 371 | Yes | Yes | No | rbcL, ITS2, trnH-psbA |
| Solanales | Convolvulaceae | *Aniseia martinicensis* (Jacq.) Choisy | CANGI613-17 | Harley R.M. 57973 | Yes | Yes | No | rbcL, ITS2 |
| Solanales | Convolvulaceae | *Camonea umbellata* (L.) A.R. Simões & Staples | CANGA429-17 | Vasconcelos L.V. 900 | No | No | Yes | rbcL |
| Solanales | Convolvulaceae | *Cuscuta insquamata* Yunck. | CANGA240-17 | Mota N.F.O. 3434 | Yes | Yes | Yes | rbcL, ITS2, atpF-atpH |
| Solanales | Convolvulaceae | *Distimake macrocalyx* (Ruiz & Pav.) A.R. Simões & Staples | CANGA268-17 | Vasconcelos L.V. 882 | Yes | Yes | Yes | rbcL, ITS2 |
| Solanales | Convolvulaceae | *Distimake macrocalyx* (Ruiz & Pav.) A.R. Simões & Staples | CANGA463-20 | Harley R.M. 57866 | Yes | No | Yes | rbcL, ITS2 |
| Solanales | Convolvulaceae | *Evolvulus filipes* Mart. | CANGA196-17 | Vasconcelos L.V. 810 | Yes | Yes | Yes | rbcL, ITS2, trnH-psbA |
| Solanales | Convolvulaceae | *Evolvulus filipes* Mart. | CANGI625-17 | Pastore M. 357 | Yes | No | Yes | ITS2 |
| Solanales | Convolvulaceae | *Ipomoea asplundii* O'Donell | CANGA026-17 | Vasconcelos L.V. 1102 | Yes | No | Yes | rbcL, ITS2 |
| Solanales | Convolvulaceae | *Ipomoea cavalcantei* D.F.Austin | ITVPL305-17 | Harley R.M. 57491 | Yes | Yes | Yes | rbcL, ITS2 |
| Solanales | Convolvulaceae | *Ipomoea cavalcantei* D.F.Austin | CANGA119-17 | Vasconcelos L.V. 1121 | Yes | No | Yes | rbcL, ITS2 |
| Solanales | Convolvulaceae | *Ipomoea goyazensis* Gardner | CANGA219-17 | Vasconcelos L.V. 1116 | Yes | No | Yes | rbcL, ITS2 |
| Solanales | Convolvulaceae | *Ipomoea goyazensis* Gardner | CGII630-20 | Zappi D.C. 4354 | Yes | No | Yes | rbcL |
| Solanales | Convolvulaceae | *Ipomoea marabaensis* D.F.Austin & Secco | CANGA271-17 | Viana P.L. 5642 | Yes | Yes | Yes | ITS2 |
| Solanales | Convolvulaceae | *Ipomoea marabaensis* D.F.Austin & Secco | CANGA272-17 | Viana P.L. 5642 | Yes | Yes | Yes | ITS2 |
| Solanales | Convolvulaceae | *Ipomoea marabaensis* D.F.Austin & Secco | ITVPL296-17 | Harley R.M. 57434 | Yes | Yes | Yes | rbcL, ITS2, trnH-psbA |
| Solanales | Convolvulaceae | *Ipomoea marabaensis* D.F.Austin & Secco | ITVPL297-17 | Harley R.M. 57447 | Yes | Yes | Yes | rbcL, ITS2, trnH-psbA |
| Solanales | Convolvulaceae | *Ipomoea marabaensis* D.F.Austin & Secco | ITVPL298-17 | Harley R.M. 57462 | Yes | Yes | Yes | rbcL, ITS2, trnH-psbA |
| Solanales | Convolvulaceae | *Ipomoea marabaensis* D.F.Austin & Secco | ITVPL301-17 | Viana P.L. 6188 | Yes | Yes | Yes | rbcL, ITS2 |
| Solanales | Convolvulaceae | *Ipomoea marabaensis* D.F.Austin & Secco | ITVPL302-17 | Viana P.L. 6210 | Yes | Yes | Yes | rbcL, ITS2 |
| Solanales | Convolvulaceae | *Ipomoea marabaensis* D.F.Austin & Secco | ITVPL303-17 | Viana P.L. 6234 | Yes | Yes | Yes | rbcL, ITS2 |
| Solanales | Convolvulaceae | *Ipomoea marabaensis* D.F.Austin & Secco | CANGA273-17 | Harley R.M. 58166 | Yes | No | Yes | rbcL, ITS2 |
| Solanales | Convolvulaceae | *Ipomoea marabaensis* D.F.Austin & Secco | ITVPL304-17 | Viana P.L. 6110 | Yes | Yes | Yes | rbcL, ITS2 |
| Solanales | Convolvulaceae | *Ipomoea marabaensis* D.F.Austin & Secco | ITVPL306-17 | Harley R.M. 57493 | Yes | Yes | Yes | rbcL, ITS2 |
| Solanales | Convolvulaceae | *Ipomoea marabaensis* D.F.Austin & Secco | ITVPL307-17 | Harley R.M. 57503 | Yes | Yes | Yes | rbcL, ITS2 |
| Solanales | Convolvulaceae | *Ipomoea marabaensis* D.F.Austin & Secco | CANGA277-17 | Vasconcelos L.V. 1079 | Yes | No | Yes | rbcL, ITS2 |
| Solanales | Convolvulaceae | *Ipomoea marabaensis* D.F.Austin & Secco | CANGA274-17 | Vasconcelos L.V. 1120 | Yes | No | Yes | rbcL, ITS2 |
| Solanales | Convolvulaceae | *Ipomoea marabaensis* D.F.Austin & Secco | CANGA275-17 | Vasconcelos L.V. 1125 | Yes | No | Yes | rbcL, ITS2 |
| Solanales | Convolvulaceae | *Ipomoea marabaensis* D.F.Austin & Secco | CANGA276-17 | Vasconcelos L.V. 1150 | Yes | No | Yes | rbcL, ITS2 |
| Solanales | Convolvulaceae | *Ipomoea marabaensis* D.F.Austin & Secco | CANGI114-17 | Harley R.M. 58132 | Yes | No | Yes | rbcL, ITS2 |
| Solanales | Convolvulaceae | *Ipomoea maurandioides* Meisn. | ITVPL282-17 | Cardoso A. 2007 | Yes | Yes | Yes | rbcL, ITS2, matK, rpoC1, atpF-atpH, trnH-psbA |
| Solanales | Convolvulaceae | *Ipomoea maurandioides* Meisn. | ITVPL299-17 | Vasconcelos L.V. 777 | Yes | Yes | Yes | rbcL, ITS2, trnH-psbA |
| Solanales | Convolvulaceae | *Ipomoea maurandioides* Meisn. | ITVPL300-17 | Vasconcelos L.V. 871 | Yes | Yes | Yes | rbcL, ITS2, rpoC1 |
| Solanales | Convolvulaceae | *Ipomoea maurandioides* Meisn. | CGII665-20 | Zappi D.C. 4501 | Yes | No | Yes | rbcL, ITS2 |
| Solanales | Convolvulaceae | *Ipomoea procumbens* Mart. ex Choisy | CANGA357-17 | Vasconcelos L.V. 1088 | Yes | No | No | rbcL, ITS2 |
| Solanales | Convolvulaceae | *Ipomoea quamoclit* L. | CANGA367-17 | Nogueira M.G.C. 665 | No | No | No | rbcL, ITS2 |
| Solanales | Convolvulaceae | *Ipomoea regnelli* Meisn. | ITVPL310-17 | Pastore M. 429 | No | Yes | Yes | rbcL, ITS2 |
| Solanales | Convolvulaceae | *Ipomoea setifera* Poir. | ITVPL311-17 | Pastore M. 431 | Yes | Yes | Yes | rbcL, ITS2 |
| Solanales | Convolvulaceae | *Ipomoea squamosa* Choisy | ITVPL308-17 | Vasconcelos L.V. 899 | No | No | Yes | rbcL, ITS2 |
| Solanales | Convolvulaceae | *Ipomoea triloba* L. | CANGA428-17 | Nogueira M.G.C. 651 | No | No | No | rbcL, ITS2 |
| Solanales | Convolvulaceae | *Ipomoea triloba* L. | CANGA220-17 | Nogueira M.G.C. 676 | No | No | No | rbcL, ITS2 |
| Solanales | Convolvulaceae | *Jacquemontia tamnifolia* (L.) Griseb. | CANGA417-17 | Harley R.M. 57430 | Yes | Yes | No | rbcL, ITS2, matK, rpoB, rpoC1, atpF-atpH, psbK-psbI, trnH-psbA |
| Solanales | Convolvulaceae | *Jacquemontia tamnifolia* (L.) Griseb. | CANGA415-17 | Harley R.M. 57460 | Yes | Yes | No | rbcL, ITS2, trnH-psbA |
| Solanales | Convolvulaceae | *Jacquemontia tamnifolia* (L.) Griseb. | CANGI113-17 | Praia T.S. 2 | Yes | Yes | No | rbcL, ITS2, rpoB, rpoC1, atpF-atpH |
| Solanales | Convolvulaceae | *Jacquemontia tamnifolia* (L.) Griseb. | CANGA416-17 | Pastore M. 303 | Yes | Yes | No | rbcL, ITS2 |
| Solanales | Convolvulaceae | *Odonellia hirtiflora* (M.Martens & Galeotti) K.R.Robertson | CANGA229-17 | Pastore M. 488 | No | Yes | No | ITS2, trnH-psbA |
| Solanales | Hydroleaceae | *Hydrolea spinosa* L. | CANGA407-17 | Vasconcelos L.V. 901 | No | No | No | rbcL, ITS2 |
| Solanales | Solanaceae | *Solanum campaniforme* Roem. & Schult. | CANGI593-17 | Harley R.M. 57312 | Yes | Yes | Yes | rbcL, ITS2, rpoB, rpoC1 |
| Solanales | Solanaceae | *Solanum rugosum* Dunal | CANGI594-17 | Harley R.M. 57424 | Yes | Yes | No | rbcL, ITS2, matK, trnH-psbA |
| Solanales | Solanaceae | *Solanum semotum* M.Nee | CANGI117-17 | Pastore M. 563 | Yes | No | Yes | rbcL, ITS2 |
| Solanales | Solanaceae | *Solanum sisymbriifolium* Lam. | CANGI597-17 | Pastore M. 574 | Yes | No | No | rbcL, ITS2 |
| Solanales | Solanaceae | *Solanum subinerme* Jacq. | CANGA413-17 | Hiura A.L. 75 | Yes | Yes | No | rbcL, ITS2 |
| Solanales | Solanaceae | *Solanum velutinum* Dunal | CANGI595-17 | Trindade J.R. 343 | Yes | Yes | Yes | ITS2, matK, rpoB |
| Solanales | Solanaceae | *Solanum velutinum* Dunal | CANGI596-17 | Afonso E.A.L 136 | Yes | Yes | Yes | rbcL, ITS2, rpoB, atpF-atpH, psbK-psbI |
| Vitales | Vitaceae | *Cissus erosa* Rich. | CANGA174-17 | Costa J.L.C. 6 | Yes | Yes | No | rbcL, ITS2, matK, rpoB, rpoC1, psbK-psbI |
| Vitales | Vitaceae | *Cissus erosa* Rich. | CANGA175-17 | Pastore M. 356 | Yes | Yes | No | ITS2 |
| Vitales | Vitaceae | *Cissus erosa* Rich. | CANGI617-17 | Harley R.M. 58078 | Yes | No | No | rbcL, ITS2 |
| Vitales | Vitaceae | *Cissus verticillata* (L.) Nicolson & C.E.Jarvis | CANGA445-20 | Mota N.F.O. 3445 | Yes | Yes | No | rbcL, ITS2, matK, rpoB, rpoC1, atpF-atpH, psbK-psbI |
| Vitales | Vitaceae | *Cissus verticillata* (L.) Nicolson & C.E.Jarvis | CANGI616-17 | Harley R.M. 58049 | Yes | No | No | rbcL, ITS2 |
| Vitales | Vitaceae | *Clematicissus simsiana* (Schult. & Schult.f.) Lombardi | CANGI615-17 | Pastore M. 503 | No | No | Yes | rbcL, ITS2 |
| Zingiberales | Costaceae | *Chamaecostus lanceolatus* subsp. *pulchriflorus* (Ducke) C.D.Specht & D.W.Stev. | CANGI118-17 | Prado M.L 368 | No | Yes | Yes | rbcL, ITS2, matK, rpoB, atpF-atpH, psbK-psbI |
| Zingiberales | Costaceae | *Chamaecostus lanceolatus* subsp. *pulchriflorus* (Ducke) C.D.Specht & D.W.Stev. | CANGI119-17 | Prado M.L 368 | No | Yes | Yes | rbcL, matK, atpF-atpH, psbK-psbI |
| Zingiberales | Costaceae | *Chamaecostus lanceolatus* subsp. *pulchriflorus* (Ducke) C.D.Specht & D.W.Stev. | CANGI120-17 | Afonso E.A.L 134 | No | Yes | Yes | matK, psbK-psbI |
| Zingiberales | Costaceae | *Chamaecostus lanceolatus* subsp. *pulchriflorus* (Ducke) C.D.Specht & D.W.Stev. | CANGI121-17 | Nunes C.S. 104 | No | Yes | Yes | rbcL, rpoC1, atpF-atpH |
| Zingiberales | Costaceae | *Costus arabicus* L. | CANGI126-17 | Pastore M. 541 | No | No | No | rbcL, ITS2 |
| Zingiberales | Costaceae | *Costus scaber* Ruiz & Pav. | CANGI123-17 | Prado M.L 369 | Yes | Yes | No | rbcL, matK |
| Zingiberales | Costaceae | *Costus scaber* Ruiz & Pav. | CANGI124-17 | Nunes C.S. 90 | Yes | Yes | No | rbcL, atpF-atpH |
| Zingiberales | Costaceae | *Costus spiralis* (Jacq.) Roscoe | CANGI125-17 | Nunes C.S. 93 | Yes | Yes | No | rbcL, rpoC1, atpF-atpH |
| Zingiberales | Heliconiaceae | *Heliconia acuminata* L.C.Rich. | CANGI270-17 | Pastore M. 542 | No | No | Yes | rbcL, ITS2 |
| Zingiberales | Heliconiaceae | *Heliconia acuminata* L.C.Rich. | CANGI272-17 | Nogueira M.G.C. 675 | No | No | Yes | rbcL, ITS2 |
| Zingiberales | Heliconiaceae | *Heliconia acuminata* L.C.Rich. | CANGI271-17 | Pastore M. 579 | No | No | Yes | rbcL |
| Zingiberales | Heliconiaceae | *Heliconia densiflora* Verl. | CANGI269-17 | Harley R.M. 57954 | No | Yes | Yes | rbcL, ITS2 |
| Zingiberales | Marantaceae | *Calathea lutea* (Aubl.) Schult. | CANGI336-17 | Nogueira M.G.C. 663 | No | No | No | rbcL, ITS2 |
| Zingiberales | Marantaceae | *Ctenanthe ericae* C.L.Andersson | CANGI455-17 | Prado M.L 373 | No | Yes | Yes | rbcL, ITS2, matK, rpoB, atpF-atpH, psbK-psbI, trnH-psbA |
| Zingiberales | Marantaceae | *Ctenanthe ericae* C.L.Andersson | CANGI456-17 | Prado M.L 373 | No | Yes | Yes | rbcL, ITS2, matK, rpoC1, atpF-atpH, psbK-psbI |
| Zingiberales | Marantaceae | *Goeppertia allouia* (Aubl.) Borchs. & S. Suárez | CANGI338-17 | Nogueira M.G.C. 677 | No | No | Yes | ITS2 |
| Zingiberales | Marantaceae | *Goeppertia barbata* (Petersen) Borchs. & S.Suárez | CANGI339-17 | Harley R.M. 58053 | No | No | Yes | rbcL, ITS2 |
| Zingiberales | Marantaceae | *Ischnosiphon arouma* (Aubl.) Körn. | CANGI335-17 | Harley R.M. 57986 | No | Yes | No | rbcL, ITS2 |
| Zingiberales | Marantaceae | *Monotagma plurispicatum* (Körn.) K.Schum. | CANGI337-17 | Pastore M. 543 | Yes | No | Yes | rbcL |
| Zingiberales | Zingiberaceae | *Renealmia alpinia* (Rottb.) Maas | CANGI619-17 | Prado M.L 374 | No | Yes | No | rbcL, ITS2, matK, trnH-psbA |
| Zingiberales | Zingiberaceae | *Renealmia alpinia* (Rottb.) Maas | CANGI620-17 | Prado M.L 374 | No | Yes | No | rbcL, matK, rpoB, trnH-psbA |

**Table A2.** Primers used to generate DNA barcodes for the plant species of the canga of Serra dos Carajás and related regions in the Brazilian state of Pará, Eastern Amazon, as used and referred by Babiychuk et al. (2017).

| Region | Primer | Sequence (5’-3’) |
| --- | --- | --- |
| *rbc*L | rbcLb-Sf | AGACCTTTTTGAAGAAGGTTCTGT |
|  | rbcLb-Sr | TCGGTCAGAGCAGGCATATGCCA |
| ITS2 | S2F | ATGCGATACTTGGTGTGAAT |
|  | S3R | GACGCTTCTCCAGACTACAAT |
| *mat*K | 1R-KIM-f | ACCCAGTCCATCTGGAAATCTTGGTTC |
|  | 3F-KIM-r | CGTACAGTACTTTTGTGTTTACGAG |
| *rpo*B | rpoB-1f | AAGTGCATTGTTGGAACTGG |
|  | rpoB-4r | GATCCCAGCATCACAATTCC |
| *rpo*C1 | rpoC1-2f | GGCAAAGAGGGAAGATTTCG |
|  | rpoC1 4r | CCATAAGCATATCTTGAGTTGG |
| *atp*F_*atp*H | atpF | ACTCGCACACACTCCCTTTCC |
|  | atpH | GCTTTTATGGAAGCTTTAACAAT |
| *psb*K-*psb*I | psbK | TTAGCCTTTGTTTGGCAAG |
|  | psbI | AGAGTTTGAGAGTAAGCAT |
| *trn*H-*psb*A | trnH | CGCGCATGGTGGATTCACAATCC |
|  | psbA | GTTATGCATGAACGTAATGCTC |

**Table A3.** Samples collected for the DNA metabarcoding analysis using bulk samples of collected leaves, indicating vegetation characteristics and the coordinates of the sampling spots.

| Sampling spots | Vegetation | Coordinates |
| --- | --- | --- |
| FG1 | Forest grove, N1, Serra Norte, Parauapebas, Pará | 5°59'38.8"S 50°18'57.0"W |
| FG2 | Forest grove, N1, Serra Norte, Parauapebas, Pará | 5°58'20.6"S 50°19'20.0"W |
| FG3 | Forest grove, S11D, Serra Sul, Canaã dos Carajás, Pará | 6°23'35.1"S 50°22'08.4"W |
| OV1 | Open rupestrian vegetation, surroundings of the Marco Zero Lake (temporary), N1, Serra Norte, Parauapebas, Pará | 6°00'45.5"S 50°17'49.3"W |
| OV2 | Open rupestrian vegetation, surroundings of a temporary river trough, S11C, Serra Sul, Canaã dos Carajás, Pará | 6°22'57.7"S 50°22'53.6"W |
| OV3 | Open rupestrian vegetation, surroundings of the Amendoim Lake (perennial), S11D, Serra Sul, Canaã dos Carajás, Pará | 6°23'43.1"S 50°22'23.7"W |

**Table A4.** Genera without previous DNA barcode records in the BOLD database, indicating their respective families and species sampled in the present work.

| **Family** | **Genus** | **Species** |
| --- | --- | --- |
| Asteraceae | *Cavalcantia* | *Cavalcantia percymosa* |
| Asteraceae | *Monogereion* | *Monogereion carajensis* |
| Asteraceae | *Parapiqueria* | *Parapiqueria cavalcantei* |
| Asteraceae | *Praxelis* | *Praxelis asperulacea* |
| Bignoniaceae | *Lundia* | *Lundia corymbifera* |
| Bignoniaceae | *Pachyptera* | *Pachyptera incarnata* |
| Bignoniaceae | *Pleonotoma* | *Pleonotoma melioides* |
| Bignoniaceae | *Pleonotoma* | *Pleonotoma orientalis* |
| Celastraceae | *Anthodon* | *Anthodon decussatus* |
| Chrysobalanaceae | *Moquilea* | *Moquilea egleri* |
| Convolvulaceae | *Camonea* | *Camonea umbellata* |
| Convolvulaceae | *Distimake* | *Distimake macrocalyx* |
| Costaceae | *Chamaecostus* | *Chamaecostus lanceolatus* subsp. *pulchriflorus* |
| Fabaceae | *Periandra* | *Periandra mediterranea* |
| Lamiaceae | *Cantinoa* | *Cantinoa mutabilis* |
| Lamiaceae | *Mesosphaerum* | *Mesosphaerum pectinatum* |
| Loranthaceae | *Passovia* | *Passovia pedunculata* |
| Loranthaceae | *Passovia* | *Passovia pyrifolia* |
| Lycopodiaceae | *Palhinhaea* | *Palhinhaea cernua* |
| Melastomataceae | *Noterophila* | *Noterophila crassipes* |
| Poaceae | *Actinocladum* | *Actinocladum verticillatum* |
| Poaceae | *Hildaea* | *Hildaea breviscrobs* |
| Poaceae | *Hildaea* | *Hildaea* sp. |
| Poaceae | *Hildaea* | *Hildaea tenuis* |
| Poaceae | *Paratheria* | *Paratheria prostrata* |
| Poaceae | *Parodiolyra* | *Parodiolyra luetzelburgii* |
| Poaceae | *Raddiella* | *Raddiella esenbeckii* |
| Poaceae | *Rhytachne* | *Rhytachne gonzalezii* |
| Poaceae | *Trichanthecium* | *Trichanthecium nervosum* |
| Polygalaceae | *Caamembeca* | *Caamembeca spectabilis* |
| Rubiaceae | *Borreria* | *Borreria alata* |
| Rubiaceae | *Borreria* | *Borreria carajasensis* |
| Rubiaceae | *Borreria* | *Borreria heteranthera* |
| Rubiaceae | *Borreria* | *Borreria hispida* |
| Rubiaceae | *Borreria* | *Borreria ocymifolia* |
| Rubiaceae | *Borreria* | *Borreria paraensis* |
| Rubiaceae | *Borreria* | *Borreria semiamplexicaulis* |
| Rubiaceae | *Borreria* | *Borreria tenella* |
| Rubiaceae | *Borreria* | *Borreria verticillata* |
| Rubiaceae | *Carajasia* | *Carajasia cangae* |
| Rubiaceae | *Cordiera* | *Cordiera myrciifolia* |
| Rubiaceae | *Cordiera* | *Cordiera sessilis* |
| Rubiaceae | *Perama* | *Perama carajensis* |
| Rubiaceae | *Perama* | *Perama hirsuta* |
| Rutaceae | *Ertela* | *Ertela trifolia* |
| Rutaceae | *Metrodorea* | *Metrodorea flavida* |
| Rutaceae | *Rauia* | *Rauia* cf. *prancei* |
